# *Operando* Electron Microscopy of Catalysts: The Missing Cornerstone in Heterogeneous Catalysis Research?

**DOI:** 10.1021/acs.chemrev.3c00352

**Published:** 2023-11-15

**Authors:** See Wee Chee, Thomas Lunkenbein, Robert Schlögl, Beatriz Roldán Cuenya

**Affiliations:** † Department of Interface Science, Fritz-Haber Institute of the Max-Planck Society, 14195 Berlin, Germany; ‡ Department of Inorganic Chemistry, Fritz-Haber Institute of the Max-Planck Society, 14195 Berlin, Germany

## Abstract

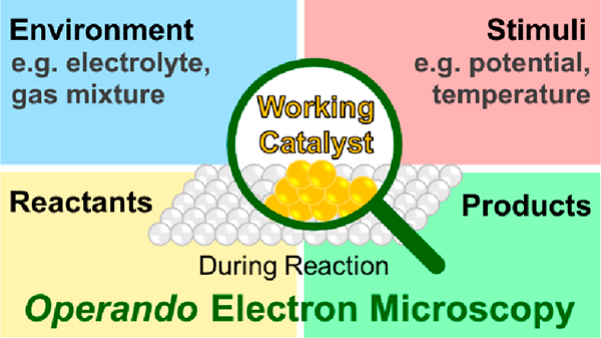

Heterogeneous catalysis in thermal gas-phase and electrochemical liquid-phase chemical conversion plays an important role in our modern energy landscape. However, many of the structural features that drive efficient chemical energy conversion are still unknown. These features are, in general, highly distinct on the local scale and lack translational symmetry, and thus, they are difficult to capture without the required spatial and temporal resolution. Correlating these structures to their function will, conversely, allow us to disentangle irrelevant and relevant features, explore the entanglement of different local structures, and provide us with the necessary understanding to tailor novel catalyst systems with improved productivity. This critical review provides a summary of the still immature field of *operando* electron microscopy for thermal gas-phase and electrochemical liquid-phase reactions. It focuses on the complexity of investigating catalytic reactions and catalysts, progress in the field, and analysis. The forthcoming advances are discussed in view of correlative techniques, artificial intelligence in analysis, and novel reactor designs.

## 1 Introduction

The chemical conversion of small molecules on the surface of heterogeneous catalysts forms the backbone of many processes in the modern chemical industry.^1^ In fact, almost every organic molecule that is synthesized to simplify our modern daily lives has interacted with the surface of a catalyst in at least one of its synthesis steps.^2^ In heterogeneous catalysis, solids interacting with reactants in the gas or liquid phase are used to accelerate chemical reactions by providing alternative and energetically more efficient reaction pathways.^3^ With the current need to find viable alternatives to fossils fuels, heterogeneous catalysts are further emerging as promising energy converters that can efficiently and reversibly convert electrical energy into chemical energy and back.^4−6^ Examples of such chemical fuels include methanol or ammonia—molecules that store the energy in chemical bonds which can be released subsequently upon decomposition in fuel cells or with reforming catalysts. Interestingly, almost all catalyst systems currently used in industry were developed by empirical optimization.^7^ However, the practicality of this approach is increasingly challenged by the rising demand for even more efficient catalysts, especially as we look toward an energy infrastructure that is based on renewable energy sources. It is therefore necessary to accelerate catalyst discovery by focusing our efforts on tailored catalysts that are designed based on a detailed knowledge of the relevant working structures.

Pioneering work from Gerhart Ertl^8^ using photoemission electron microscopy has shown that the catalyst surface is not static and instead changes constantly during a chemical reaction. Nowadays, it is also generally accepted that catalysts restructure in response to changes in their reaction environment, which can lead to metastable, high-energy structures that are only stable under the applied conditions.^9,10^ Hence, unless these structures are kinetically trapped, they may not be preserved after the sample is removed from the reaction environment. On the other hand, irreversible transformations,^11,12^ such as deactivation, tend to lead to thermodynamically stable phases that are robust enough to endure subsequent inspection. This uncertainty in the preservation of operating catalyst structures complicates efforts to use the samples obtained after the reaction and to interpret their performance trends. If we are to understand how the morphology of a catalyst is associated with its relevant performance metrics (i.e., activity, selectivity, and productivity^13^), it is crucial that we reveal the structure and composition of a working catalyst under reaction conditions.^14−16^

**Figure 1:**
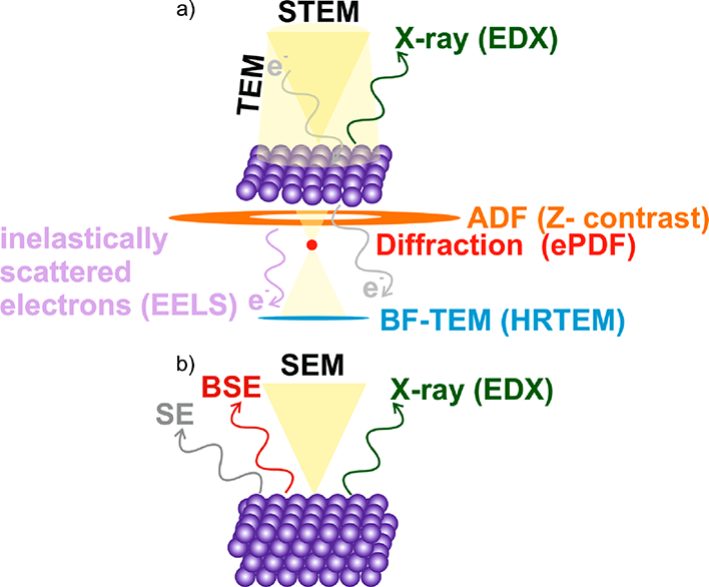
Extracting local chemical information from nanomaterials by electron microscopy. (a) Scanning (S)TEM allows not only recording the electron diffraction pattern which can be used to establish pair distribution functions (ePDF), bright-field (BF)-TEM, phase contrast (high-resolution (HR)TEM), and annular dark-field (ADF) images that are based on elemental (*Z*) contrast but also X-rays and inelastically scattered electrons using dedicated hardware for energy-dispersive X-ray spectrometry (EDX) and electron energy loss spectroscopy (EELS). (b) SEM imaging of the surface of a catalyst. Secondary electrons (SEs) and backscattered electrons (BSEs) in combination with EDX analysis are mainly detected.

It is, however, often difficult to determine the features of industrially applied catalysts that are important for catalytic turnover because these materials are usually inhomogeneous on the nanoscale. Insight into these complicated structures and how they influence the catalytic outcome, such as the interplay of atomic scale defects with macroscopic transport processes, is key for rational catalyst design.^7^ Electron microscopy (EM), particularly transmission electron microscopy (TEM) and scanning electron microscopy (SEM), has been an indispensable tool for elucidating the structure, composition, and chemistry of solid catalysts from the macro down to the atomic scale (Figure 1).^17^ EM plays a unique role among the various analytical techniques that can be used to study catalysts with its ability to resolve local structures on the nanoscale and in real space. In fact, it is quite difficult nowadays to find any study on heterogeneous catalysts that does not include at least one SEM or TEM image (often only of the as-synthesized catalyst or “pre-catalyst”). The resolving power of top-of-the-line TEMs has also reached a point where we can now perform atomic-level structural and chemical analysis of solid materials.^18−21^ Nonetheless, it is not always straightforward to use conventional EM to understand how the structure and morphology of a catalyst determines its catalytic performance. The critical question lies in whether the structure characteristics captured within the vacuum environment of an EM are representative of the working states of the catalysts under reaction conditions, which are different from their pristine states.^22^

**Figure 2:**
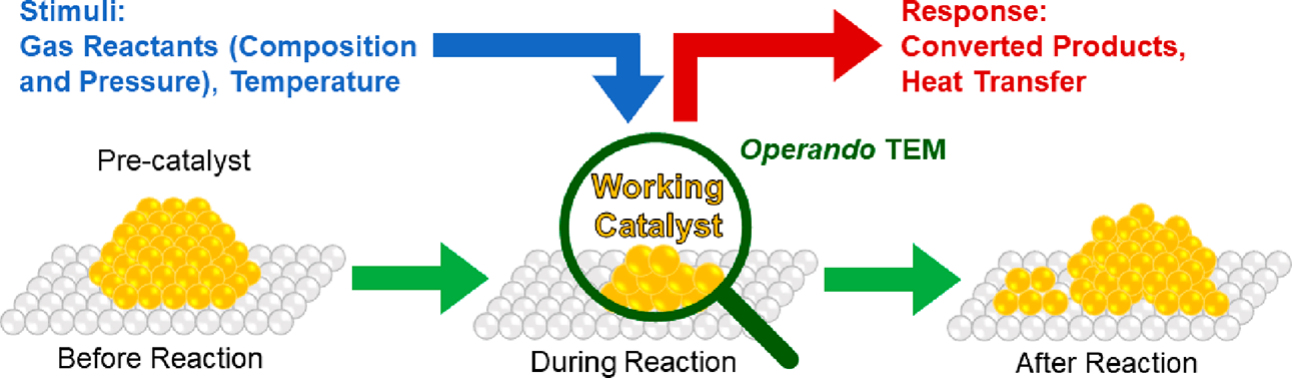
Schematic showing an *operando* electron microscopy experiment where the changes in catalyst morphology during a heterogeneous, gas phase, thermal catalysis reaction are probed. Changing the chemical potential by applying different temperatures and partial or total pressures alters the catalyst and its performance, rendering the detection of conversion mandatory.

Recently, the development and commercialization of EMs and TEM holders with environmental capabilities have made *in situ* EM more accessible to the general research community. These tools allow us to use the immense resolving power of a TEM to visualize particulate catalysts under reaction conditions at nanometer to subnanometer scales, thereby providing insight into their structural and compositional response to changes in those conditions.^23,24^ More relevant for heterogeneous catalysis is what is known as *operando* studies. We emphasized here that there is an explicit difference between *operando* studies and the more general class of *in situ* work. For an *in situ* experiment to be considered *operando*,^25,26^ the catalysts need to be studied under working conditions and coupled to simultaneous measurements of the catalytic properties (e.g., catalytic turnover) (Figure 2). We will discuss this distinction further later, but it is important to be clear here that because the aim of such work is to tie the observed morphologies to their catalytic function specific conditions need to be fulfilled in these experiments. These considerations include the minimization of artifacts due to the electron beam or reaction cell design, determining the significance of observations from only a few imaged particles, how to prove that these particles or structures are active, and ascertaining whether the observed chemical kinetics or dynamics are indeed responsible for the changes in catalytic properties.

In this review, we discuss the latest results and progress made in the *operando* EM of heterogeneous catalysts, where we include SEM and TEM work. Specifically, we will focus on research that incorporates catalyst property measurements, rather than present a broad overview of *in situ* EM work on heterogeneous catalysts. Our purpose is to allow the reader to place the content of this review article in the broader perspective of catalysis and to serve as a bridge between the interested chemists and electron microscopists working on catalyst development. For a general treatment, we refer interested readers to recent review articles on the topic.^27−31^ A discussion on the fundamentals of SEM and TEM is also beyond the scope of this review, and the reader is referred to textbooks dealing with the two techniques.^32,33^ We describe generally the principles behind *operando* studies in catalysis and establish the scientific case for such work in Section 2. In Section 3, we present the current state-of-the-art in *operando* EM of thermal and electrocatalytic processes, the types of reactions we can study, and the limitations. In Section 4, we touch on the more technical aspects of imaging, diffraction, and spectroscopy in *operando* EM studies, such as achievable spatial and temporal resolution. Lastly, we provide our perspectives on the future developments in the field in Section 5, and concluding remarks are given in Sections 6.

## 2 Scientific Case for *Operando* EM Studies

Before discussing the principles of *operando* TEM measurements and analyses in detail, we believe that it is appropriate to provide a general overview of heterogeneous catalysis to help the reader place the subsequent discussion on the possibilities and perspectives of *operando* TEM in the context. Briefly, this chapter will highlight the gap between academic catalysis research and industrial application (2.1 and 2.2), the properties of a catalyst when it is placed in a reactive medium (2.3 and 2.4), and the particular challenges in the structural characterization of heterogeneous catalysts (2.5 and 2.6). Lastly, we conclude with a discussion on the possibility of visualizing active sites while at working conditions (2.7).

### 2.1 General Remarks on Heterogeneous Catalysis

**Figure 3:**
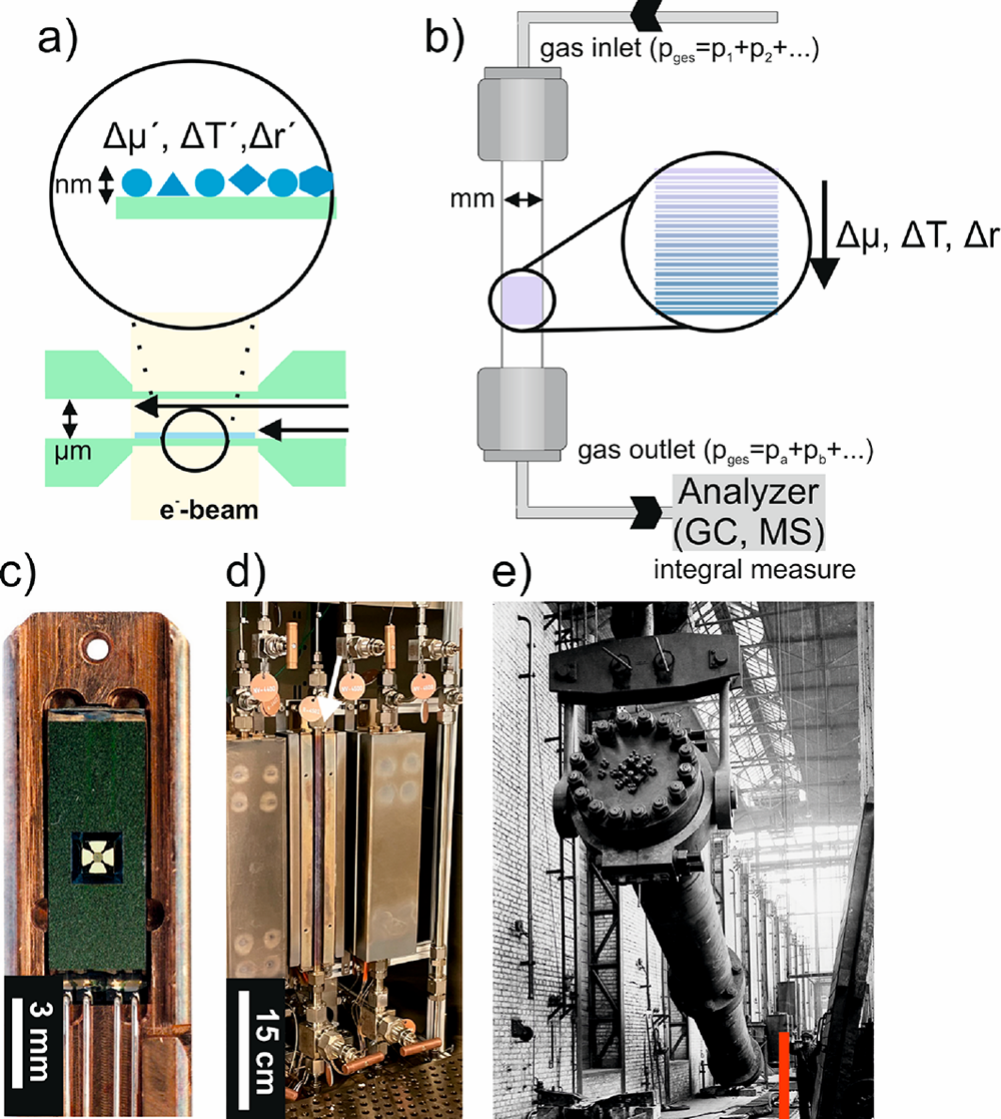
Difference in scale and sampling of a MEMS-based reactor used for *in situ* TEM studies and an illustrative example of a plug flow reactor. (a) Schematics of MEM-based closed-cell nanoreactor for *in situ* TEM analysis and (b) typically used plug flow reactor for gas-phase reactions. Δμ, Δ*T*, and Δ*r* denote the gradients in the chemical potential, temperature, and reactivity along the catalyst bed, respectively, while Δμ́, Δ*T́*, and Δ*ŕ* are the corresponding local gradients. Reproduced in part with permission from ref (30). Copyright 2021 IOP Publishing. (c) Image of the tip of a gas-phase *in situ* TEM holder in which a MEMS chip has been positioned. Reproduced in part with permission from ref (23). Copyright 2015 Elsevier. (d) Photograph of a laboratory tubular reactor setup. The white arrow points to a reactor tube that is placed in between the furnace and connected to the gas inlet and outlet. (e) Installation of a high-pressure reactor in an ammonia plant. Copyright BASF. From ref (41). Used under CC BY-NC-ND 2.0. The red bar was added to highlight the height of a human being.

Obtaining insights into catalytic processes is not trivial. In its full complexity, catalysis encompasses the different disciplines of physics, chemistry, and engineering, where the parameter space, as typical for a kinetic phenomenon, spans many factors, such as reactor and material design, catalyst bed type and packing, space–time velocity, temperature, partial total pressure, time span of the experiment, electrolyte, and applied electrical potential stability. More inconveniently, these parameters are not necessarily independent of each other. In addition, with the laboratory-scale setups used for fundamental academic research, it can be difficult to access the more extreme reaction conditions required in a practical setting (Figure 3). For example, industrial catalytic converters consist of meter-high reactors filled with tons of specially prepared shaped structures to optimize gas transport and thermal conductivity.^34^ Along the catalyst bed, the gas composition changes from top to bottom, and an analyzer at the exit of the reactor detects the summed total gas composition at the end of the reactor tube.^35−37^ The flowing gas interacts with billions of active nanoparticles (NPs), and it is not uncommon that pressures of 10 to 100 bar are applied to make the desired reaction feasible. The lifetime of a reactor filling is also often set for several years. During this time, the catalyst is exposed to constant (where energy is provided by fossil fuels) or transient (where energy is provided by renewable sources^38^) reaction conditions. It is also common to have activation protocols that require several weeks to transform the precatalyst into the most active solid and to reach a steady state.^39^ As the catalyst is exposed to extended reaction conditions, it further loses performance due to thermodynamic aging. Therefore, while it is recommended to apply identical parameters/conditions used in industrial practice due to the kinetic nature of catalysis in basic science studies, fulfilling this requirement is not always feasible, which leads to “gaps” of our understanding of the relevant catalytic processes under operating conditions.^11,40^

Today, we also know that a heterogeneous catalyst is, in fact, a complex entity that structurally responds to the presence of reactants, (partial) pressure, gas composition, temperature, applied electrical potential, pH of the electrolyte, etc.^38^ (The specific parameters depend on whether it is a thermal gas-phase or electrocatalytic liquid-phase reaction). These chemical driving forces lead to catalyst restructuring as well as transformations to metastable and reversible structures under reaction conditions, which render the working state unpredictable from thermodynamic-phase diagrams. This structural pluralism between working and as-synthesized structures may have been invisible to Ostwald when he completed his definition of the catalytic process, but he was already aware that “the dependence of this [acceleration and deceleration] on the nature and concentration of the catalysts, the temperature, the presence of other substances, etc.”^42^ affects the catalytic outcome. In general, these *in situ* generated structures are considered to be reversible under ideal conditions,^43^ but any deviation from ideality, i.e., reflecting the real world, leads to a change in performance and structure or to irreversible transformations that cause deactivation.^44^ For gas-phase thermal catalysis, reversibility holds true only for idealized adiabatic conditions, at which all parameters are kept constant, which is almost never the case in real reactions. In electrocatalysis, the applied potential is the key controlling parameter, which in turn influences the catalyst’s oxidation state. These oxidation state transitions are typically rationalized using a Pourbaix diagram that describes the stable state as a function of the applied potential and pH. The Pourbaix diagram is, nonetheless, still an idealized thermodynamic construct that does not consider the kinetic limitations of such transformations and their consequent impact on the morphology during the reaction.

As we scale up into industrial reactors, the catalytic conditions also become increasingly nonideal. Reaction gradients within a reactor system (Figure 3b) or different flow dynamics between two or more reactor systems are examples of such nonideal conditions. In addition, local conversions cause the reaction front to change its composition, resulting in locally different chemical potentials, which further downstream lead to different surface processes that change the catalytic performance along the catalyst bed.^35−37^ Hence, the reactor itself becomes part of the parameter field. Such arbitrariness is, however, not conducive to systematic studies. To gain detailed understanding into catalytic processes, it is necessary to study catalytically active particles under more limited but homogeneous reaction conditions and during operation, ideally over the entire length scale down to the atomic level. All of this requires strict adherence to empirically found reaction conditions to approximate reality in an actual reactor. The interaction between gas mixtures or electrolytes and the surface of inorganic NPs or thin films can, however, lead to structural changes via stoichiometric solid-state reactions that have nothing to do with a catalytic reaction. Therefore, measurements of the catalytic performance to ensure self-consistency are critical. Another reason large-scale catalyst systems can be difficult to reproduce pertains to how different aliquots taken from the same sample batch can differ in their intrinsic structure. These subtly different structures are then exposed to slightly different chemical potentials during reaction, which, in combination with reaction gradients that exist within the reactor, lead to different working structures and performances. The presence of the structural sensitivity of a reaction and intrinsic metastability can further complicate the situation. The difficulties in gaining knowledge in the field of heterogeneous catalysis were already anticipated by Ostwald, who stated in 1902: “It is obvious, and it must be emphasized, that all attempts to establish theories about the cause of catalytic phenomena remain useless until quantitative measurements of the kind mentioned have been made.”^42^ The prescience of this statement is now reflected in the rapid rate at which the modification of scientific instruments, including EMs, to enable *operando* investigations is taking place. Even though quantitative and integral analysis of such catalytic systems and correlation with their function has become an important aspect in the field, it is far from sufficient due to the nanoscopic heterogeneity and complexity of industrial catalyst systems.

### 2.2 General Considerations for *Operando* Studies of Heterogeneous Catalysts

While *operando* studies are a way to probe the activated catalyst, it is important that we consider two questions when we assess *operando* work. (1) Are we looking at relevant catalytic processes in our experiments, i.e., active structures versus spectator species, and (2) on which length and time scales do the working structures need to be understood to rationalize the catalytic behavior? In addition, one would also need to ensure that the measurement itself does not affect the obtained results (beam-induced artifacts).

First, we define a relevant catalytic process as one that contributes in a significant manner to changes in the function of the catalyst (i.e., participant versus spectator). Conversely, if a given structural transformation does not perturb the balance and number of fluctuating phases and processes, it will not result in a change in catalytic function and is, thus, irrelevant. Therefore, the key to differentiating whether a solid-state process is relevant to catalysis is to identify whether it contributes to a change in the active site configuration of a catalyst and, thus, its catalytic properties.

*Operando* experiment coupled conversion measurements are crucial to ensure that we are capturing relevant catalyst transformations. While there has been rapid growth of *in situ*/*operando* techniques that allow us to probe a working catalyst’s characteristics under increasingly realistic conditions, it should also be obvious that one cannot simply place an industrial reactor inside an analytical instrument. *In situ* cells are often adapted and reflect a compromise to account for the instrument’s particular geometry (see Figure 3).^30^ For example, most surface science instruments can be modified to only accommodate an upper operating pressure of a few millibars to at most a few bars during the introduction of reactant gases or volatile electrolytes. On the other hand, with more bulk sensitive techniques such as X-ray diffraction (XRD) or X-ray absorption spectroscopy (XAS) in the hard X-ray range, it is possible to build reactors, high pressure, or flow cells that more closely mimic industrial conditions. Even so, these modified reactor designs still come with altered flow or electrochemical conditions, which in turn influence the transport properties (such as diffusion, thermal conductivity, electrical potential distributions) and kinetic barriers, and always deviate from real reactor conditions to some degree. Furthermore, there are academic limitations to how far we can study the activation and lifetime of catalysts, which are often coupled to the booking specifics of the user facilities. For instance, beam times at synchrotrons are often limited to a maximum of 1 week, whereas EMs can be reserved for at best a single day in most facilities. Long-term measurements also pose a challenge to the safety infrastructure and require automatization of data acquisition.

Therefore, there will be an inevitable gap between the model studies or simplified systems in academia and real-world systems since our model studies cannot fully capture the structural complexity and also the interplay of parameters that are present in an industrial setting. In terms of structural complexity, the gap lies in comparing model systems that are typically characterized by one reactive interface, such as the case of single crystals, whereas standard multicomponent industrial catalysts encompass several additional interfaces, including a functional interface with a support of a material different from the top reactive interface. In terms of reaction parameter space, we must consider that model studies even with product detection only capture a subset of real-world working conditions as illustrated in Figures 3a and 3b. On the other hand, the analysis performed at the outlet of an industrial reactor averages over the contributions of all the material in the reactor, encompassing too all the differences caused by the variations in the local reaction conditions. Nonetheless, the model studies play an important role in current catalysis research, which is to reduce the overall complexity of the problem such that we can obtain meaningful, interpretable results about some specific aspects of the underlying catalytic processes. To ensure that our observations of model systems are relevant, it is not enough that we capture the structural transformations, but we must also be able to tie such transformations with their impact on the overall catalytic performance. This aspect can only be achieved by tracing how both the structure and catalytic performance change as a function of the externally applied parameters.

**Figure 4:**
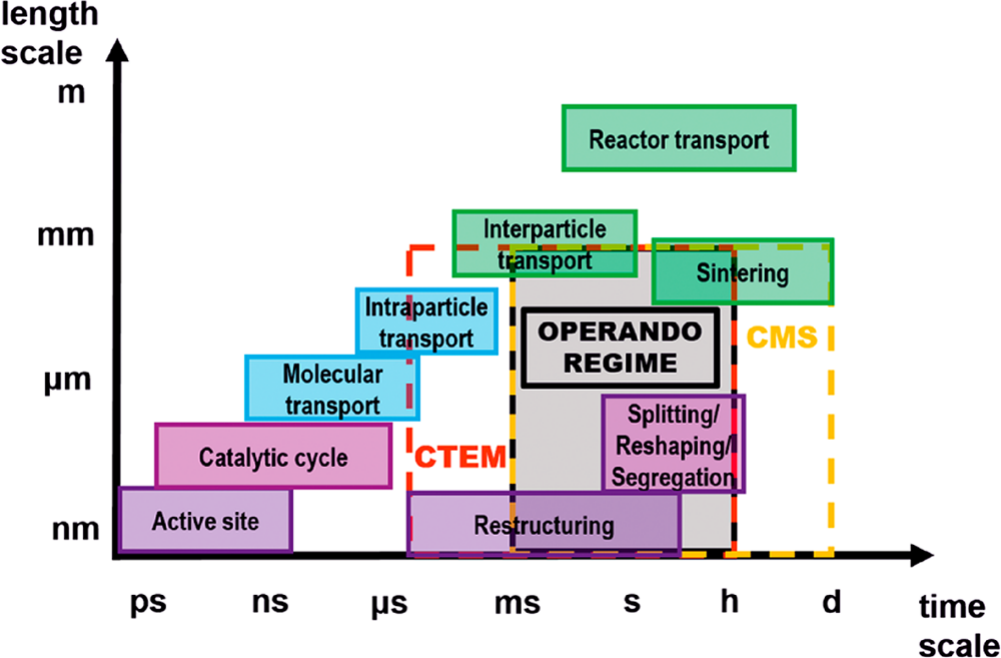
Space–time scale of different dynamic processes occurring in catalysis. Catalytic processes in their specific space and time scale ranges using the applied techniques: conventional TEM (CTEM, red dashed box) and conventional mass spectrometry (CMS, yellow dashed box). Only in the intersection of CTEM and CMS, *operando* measurements are possible (gray box). Reproduced from ref (46). Copyright 2020 Oxford University Press. CC BY.

In terms of length and time scales, the catalytic processes we can probe and interrogate are determined by the capabilities of the instruments used. The relevant length and time scales of different catalytic processes and their ranges relative to scales accessible to conventional TEM are summarized in Figure 4. The spatiotemporal quality of the sampling further determines how robustly we can associate the findings on the structure of the catalyst with its function. For example, the completion of the catalytic cycle and thus the lifetime of the active site is in the femto- to nanosecond regime,^38^ which is not resolvable by most *operando* methods. Currently, only methods based on ultrafast pump–probe spectroscopy have the requisite temporal resolution to probe charge transfer dynamics and the nature of molecular species on a catalyst’s surface on the correct time scales, although their application in catalysis studies has largely been limited to photoactive materials and photocatalytic reactions due to the physical nature of the “pump” process. It should be mentioned that there have also been attempts to resolve the active state for photocatalytic reactions using ultrafast TEM.^45^

**Figure 5:**
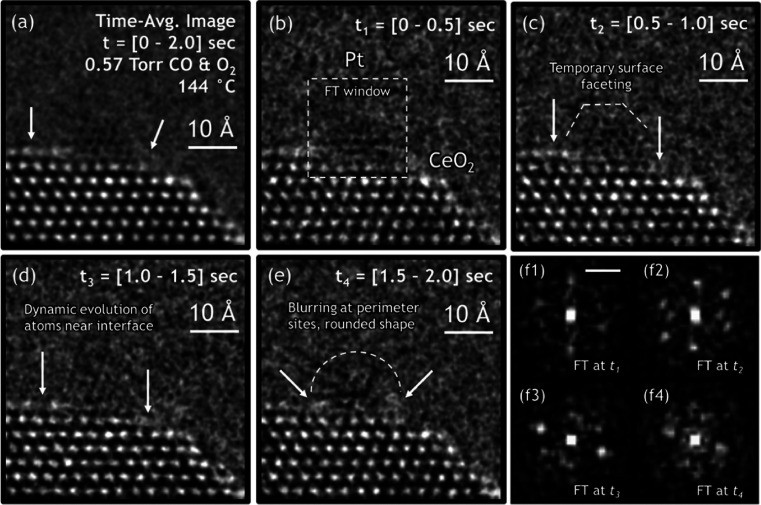
*In situ* ETEM image of a time series of a CeO_2_-supported Pt NP at 144 °C in 0.57 Torr of CO and O_2_. (a) Time-averaged image of the catalyst, obtained by summing together the individual 0.5 s exposure frames over the entire 0–2 s acquisition period. (b),(c) Atomic-scale structural dynamics that evolve over 0.5 s intervals from *t* = 0 s to *t* = 2.0 s. f1–f4 FT taken at each time interval from the windowed region around the Pt NP, as denoted in (b). The scale bar in (f1) is 5.0 nm^–1^. Reproduced from ref (47). Copyright 2021 Springer Nature. CC BY.

This issue of temporal resolution is nicely illustrated by the work of Vincent and Crozier where they looked at the behavior of small Pt NPs supported on ceria under CO oxidation reaction conditions.^47^ While the images (Figure 5) do capture the changes in a Pt NP over 0.5 s time frames, notice that only in certain frames can the lattice fringes in the NP be fully resolved. It is also important to remember here that these images are the sum of images acquired at shorter time scales, and so they only show the most stable averaged structure over a 0.5 s time frame. The features relevant for catalytic activity, i.e., the short-lived transient states of the NP as it goes from one structure to another, are, however, not captured. Furthermore, we cannot guarantee that the stable or slowly evolving phases we observe via *in situ*/*operando* EM studies (under conditions where beam-related artifacts are already minimized) are, in fact, active and participate in the reaction. The latter would require that we ascertain the physical conversion of a reactant molecule into a product molecule on the observed catalyst. Therefore, even when we incorporate product analysis, we cannot exclude the possibility that the ensemble activity is conferred by structures that exist on length scales beyond our reach or at time scales outside the temporal resolution.

The sampling rate of the online conversion measurements further comes into play (CMS, Figure 4) because for a true *operando* experiment the catalytic function of the structural changes can only be determined if both structure and conversion are tracked at similar time scales. At present, the measurements that determine catalyst function typically lag behind the structural measurements.^46^ Activity measurements in sample quantities normally used for EM studies are also difficult to achieve.

### 2.3 Chemical Potential in Catalytic Systems

Under reaction conditions, the structure of a catalyst differs from the structure of a functional solid after reaction conditions. The extent of structural change depends on the chemical potential. First, we start off by defining the term chemical potential and then describing how the local chemical potentials present during the reaction affect a catalytic system.

The chemical potential reflects the partial molar Gibbs free energy of a given system,^10,44,48,49^ and in multicomponent systems, such as heterogeneous catalysts, each component has its own chemical potential. In equilibrium, the sum of the products of the chemical potential and stoichiometric coefficients is zero. A change in the environmental conditions (e.g., temperature, pressure, or applied potential) creates a gradient in chemical potential that leads to changes in constituent components to restore equilibrium conditions. The difference of the chemical potential further depends on the number of components present in the solid and in the gas/liquid phases, including impurities and dopants. Moreover, the surface and the bulk of a catalyst can differ in structure and composition.

**Figure 6:**
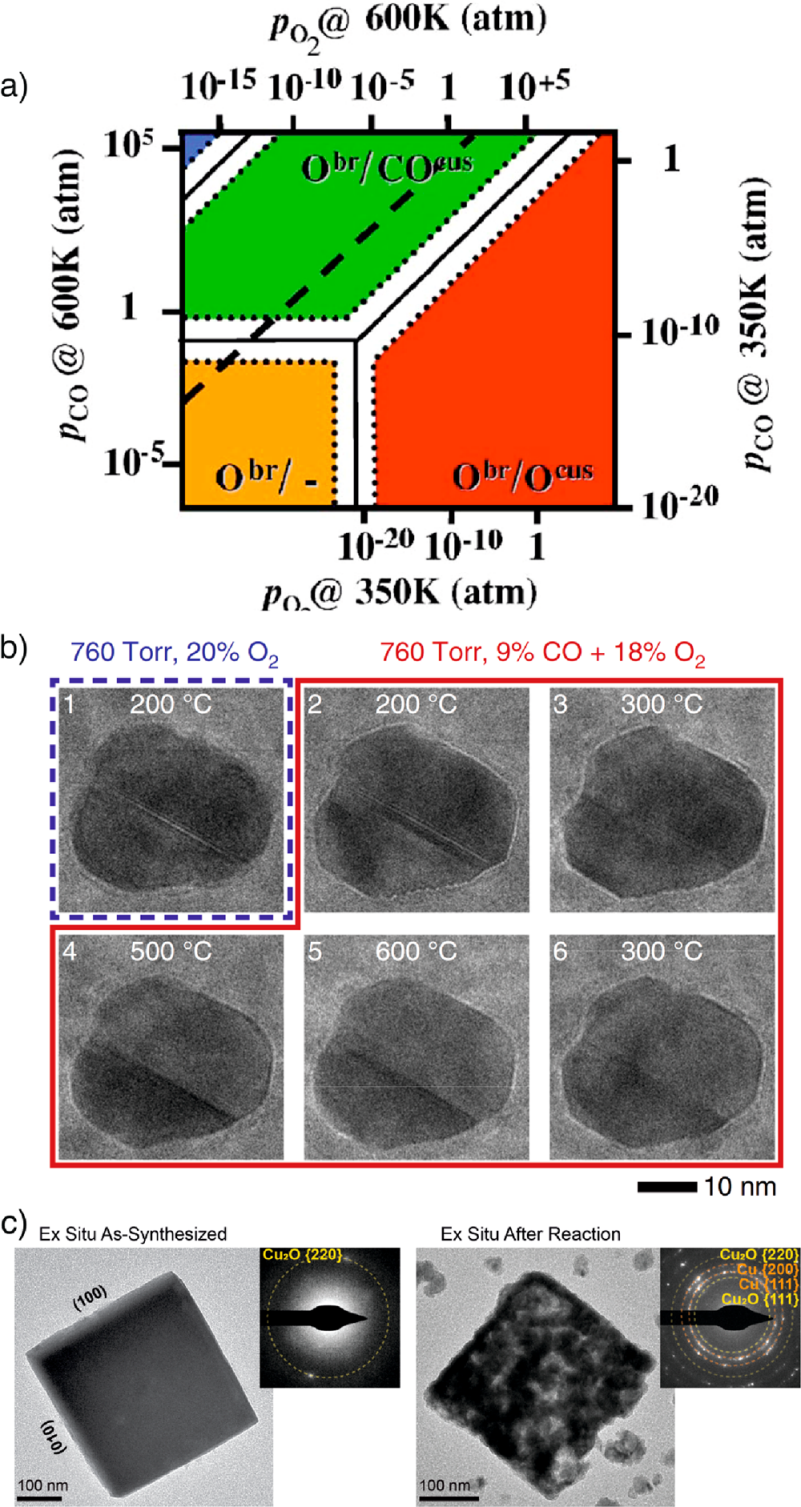
(a) First-principles calculated surface-phase diagram of CO oxidation. Regions of the lowest-energy structures in (μO, μCO) space of RuO_2_ (110). The labels note whether bridge (br) or undercoordinated sites (cus) are occupied by O or CO or are empty (−). The additional axes give the corresponding pressure scales at *T* = 300 and 600 K. In the blue-hatched region, gas-phase CO is transformed into graphite. Regions that are particularly strongly affected by kinetics are marked by white hatching. Reproduced with permission from ref (10). Copyright 2003 American Physical Society. (b) *Operando* TEM investigation showing the morphological changes of Pd NPs in gaseous environments and reversal to a faceted morphology when the reaction temperature is lowered from the higher temperatures where CO oxidation takes place. Reproduced in part from ref (50). Copyright 2020 Springer Nature. CC BY 4.0 (c) TEM images comparing cubic Cu_2_O electrocatalysts for CO_2_RR before and after reaction at −1.1 V_RHE_ for 1 h in CO_2_-saturated 0.1 M KHCO_3_. The Cu_2_O cubes are electrodeposited directly on the C-coated working electrode of a liquid cell TEM chip. Selected area electron diffraction pattern (inset) indicates that the as-synthesized cube is single-crystalline and terminated by {100} facets. During reaction, the cube became fragmented, and redeposited particles can be seen in the support background. Selected area electron diffraction pattern (inset) indicates that the cube had transformed into a fragmented structure made of polycrystalline metallic and oxidic domains. Reproduced in part from ref (51). Copyright 2021 Springer Nature. CC BY 4.0.

Structural changes during the reaction are always based on the gradient from a high chemical potential to a low chemical potential. In thermal catalysis, we can assume that the surface of a catalyst activated by thermal pretreatment is equilibrated with the bulk, and subsequent changes during the reaction are based off this structure. The working structures during the reaction are thus usually the consequence of steady-state kinetics at elevated temperatures. In electrocatalysis, the precatalysts tend to exist in a nonequilibrated state after synthesis. The precatalysts are then activated by the application of different potential protocols that will alter the catalyst morphology accordingly but not necessarily into equilibrated structures because of the slow mass transport of atoms and molecules at the ambient or relatively low temperatures of the reaction. Therefore, the restructuring of electrocatalysts often results in kinetically trapped morphologies. Regardless of the reaction’s nature, the solid catalyst will continue to change its surface shape, composition, or structure under reaction conditions until a new steady-state equilibrium is reached. *Ex situ* analysis can track these catalysis-induced changes to a certain extent, but the approach has its limits. First, it is difficult to determine whether the structural alterations are beneficial or detrimental to the catalytic conversion. Second, the quenched structures may not be the true structures present during operation but instead correspond to transitionary structures that are kinetically trapped by a comparably high energy barrier after the sample’s removal from reaction conditions. Figure 6 illustrates how some surface states can only be stable under reaction conditions of thermal catalysis and how electrolysis can create highly defective, nonequilibrated structures.

### 2.4 Chemical Dynamics during Catalysis

The chemical potential of the environment raises the catalyst material from its ground state (after activation) to an excited state, which is valid only for the specifically applied reaction conditions and thus can be discussed only for these conditions. The metastable material may be chemically very different from its parent material. Here, we must further explain the term “chemical dynamics” before we delve into the active state of a catalyst. In physical chemistry, “dynamics” refers specifically to the behavior of a system that oscillates around an average state. Within this context, an active catalyst is a dynamic system where the reactive interface is in local chemical equilibrium with a gas or liquid phase that consists of oxidizing and reducing agents. During conversion, it oscillates reversibly between two kinetically stabilized phases that continuously interconvert into each other, where the structural change induced by an oxidation wave is counteracted by a reduction wave and *vice versa*.

The challenge faced by catalyst researchers is how to excite the catalyst material energetically in such a way that one comes close to a phase transition, and the catalyst fluctuates reversibly between two phases on a time scale of picoseconds but remains active over years. It is important to highlight that at the level of highest activity or selectivity the catalyst is, in fact, thermodynamically frustrated and can be described as a frustrated phase transition. Examples include the epoxidation of ethene over Cu, where near ambient pressure X-ray photoelectron spectroscopy (NAP-XPS) experiments showed that the selectivity is highest just before the phase transition to cuprite is completed.^52^ In addition, *operando* TEM showed that while Pt catalysts are in their highest active state oxygen diffuses through the bulk, allowing the stabilization of a frustrated phase transition between Pt and PtO_*x*_.^53^ This is similar to the dry reforming of methane to syngas, where the oxide–metal phase transition is essential, as shown by ESEM experiments.^54^ These examples demonstrate the potential of *operando* EM in catalysis research to capture frustrated phase transitions, to disentangle irreversible from reversible changes, and to conclude on the importance of fluctuating dynamics, albeit occurring on much faster time scales than those that can be imaged with the EM. Consequently, the captured structural changes at the temporal resolution of TEMs represent more likely an image of the initial and final states of the frustrated phase transition, rather than the active component per se as we had discussed earlier.

**Figure 7:**
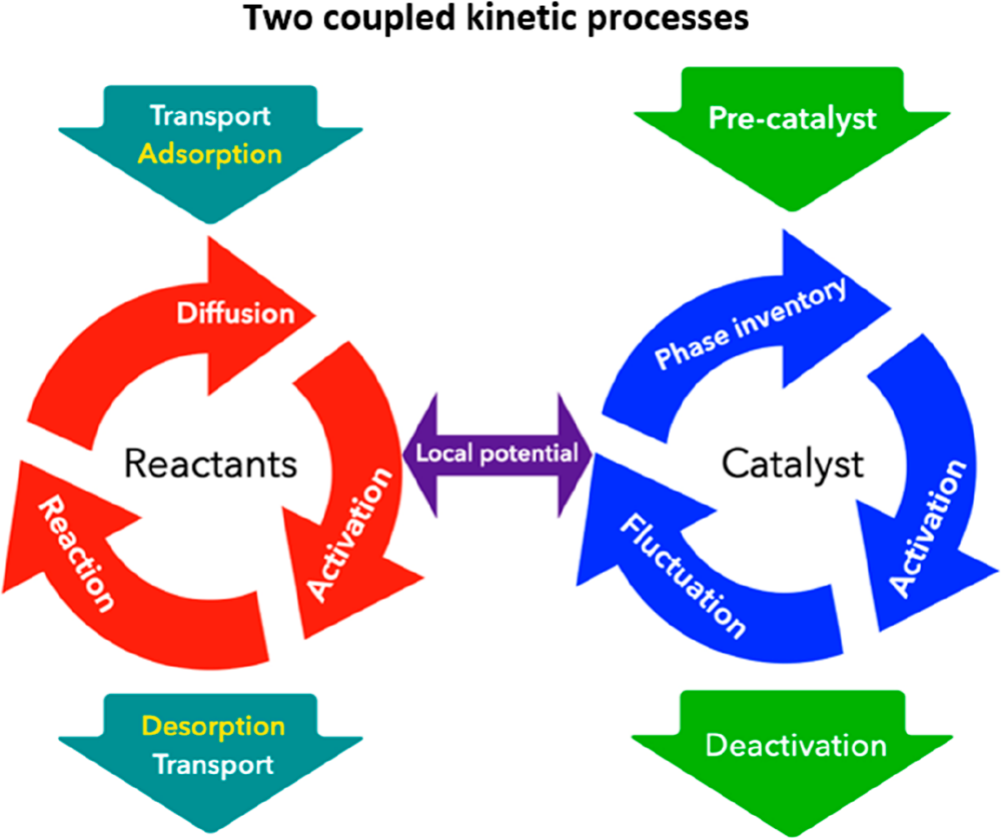
Schematic illustrating the coupled kinetic processes between the catalyst and the reactant during catalysis and how the two processes are linked via the local chemical potential.

To increase the lifetime of the catalyst, care must be taken to maintain this fluctuating process as long as possible. However, such dynamic stabilization of the active components can be removed by minute changes in the reaction parameters, such as pressure, temperature, or partial pressure/concentration variations of the reactants. In response to these changes, the catalysts also undergo a kinetic process that may lead to the completion of the phase transition and poorer performance. The global changes that arise due to chemical potential gradients and their characteristics are classified under the “chemical kinetics” of the broader system, thereby differentiating it from the “chemical dynamics” of a fluctuating active catalyst.^11,12^ We can also think of the processes involving the reactants and the catalysts as coupled kinetic processes that are linked via the local potential (see Figure 7 for a schematic describing the coupling). In a reactor, transport phenomena, which strongly influence the internal energy of a system, are decisive for these changes in the catalyst materials, which then lead to changes at the interfaces along the catalyst bed. As conversion occurs during catalysis under flow conditions, the local chemical potential is constantly changing, and thus, the catalyst evolves in response to the change. The strength of the change at the reactive interface depends on the local gradient of the chemical potential and impacts the catalytic performance, particularly if dynamic processes are replaced by irreversible transformations (i.e., the system does not recover entirely to its previous state even when identical local conditions are established again). While a dynamic catalyst can subsequently arrive at other transiently equilibrated states with different activities, these transformations will eventually result in deactivation.^12^

Due to the importance of metastable and dynamic solids during catalysis and their impact on the catalytic performance, there has been increasing experimental and theoretical work looking to map out possible polymorphs that can exist under catalytically relevant conditions. An example of such work can be found in small cluster catalysts where the term fluxionality^9,55−58^ has been coined to describe the increased availability of metastable polymorphs (versus the global minimum) and the ease of transitioning between these states under elevated temperature of a thermal catalytic reaction.

The potential energy of known metastable polymorphs can be as high as 250 meV/atom above the ground state.^59^ They are separated by energy barriers that prevent rapid transformation. The commonly applied thermal energies (about 100 meV at 900 °C) and chemical potentials (>500 meV^60^) in thermal catalysis are, however, energetically strong enough to overcome these energy barriers and to promote (surface) polymorphism or thermodynamic aging in the absence of kinetical hindrance.^39^ It should be noted that the probability of synthesizing metastable compounds increases for multinary compounds.^59^ Metastability in connection with the applied chemical potential can further modulate the extent of chemical dynamics involved, increasing complexity and altering catalytic activity.^11,12,38^

The occurrence of metastable compounds is not limited to the surface and can also affect the bulk of the nanoparticle. Carbon, for instance, can diffuse into the bulk of NPs and tune the performance of the catalytic systems. It has been observed to be dominated by hydrogenation and dehydrogenation reactions of hydrocarbons over different catalyst systems, including noble metals such as Pd, Au, and Pt.^61−64^ Therefore, essential to any interpretation is understanding the involved chemistry of carbon with the host–metal, which is imperative to catalytic research, and we have to differentiate between carbide, solid solution, or interstitial compounds. All of them have different properties. The differentiation of carbide or solid solution will also be essential for, for example, the understanding of the growth of carbon nanotubes on metallic NPs. Both phases are often not distinguishable by phase analysis using the fast Fourier transform (FFT) method or spectroscopic methods (with all the carbon in the neighborhood). Chemically, carbide and carbon alloys have different properties: a carbide cannot be easily converted into another carbide polymorph. Therefore, the carbide is considered to be a result of irreversible transformation, the formation of which is to be avoided, since it would not allow fluctuations and can cause deactivation. The carbide-carbon alloy problem is not new in materials science and occurs, for example, in the production of austenitic steel. Carbon, as an alloying component, stabilizes fcc-iron and prevents phase transformation to bcc-iron. However, care must be taken to prevent the formation of Fe_3_C (carbide, cementite); otherwise, a material with different properties will result.

Additional processes such as reduction of an oxide to its metallic phase, corrosion of the catalyst material, or deposition of solvated species in the electrolyte can further create new structural motifs. Diffusion or mass transport limitations in the reactor and species adsorption, migration, and desorption can also create local chemical gradients that lead to local compositional and structural gradients at the interface, which then renders a macroscopically homogeneous sample phase segregated and heterogeneous on the nanoscale.

### 2.5 Structural Heterogeneity in Heterogenous Catalysts

Our limited ability to resolve and characterize the structural heterogeneity of working catalysts significantly impedes our attempts to correlate catalyst structure and function as we will elaborate in the following.

**Figure 8:**
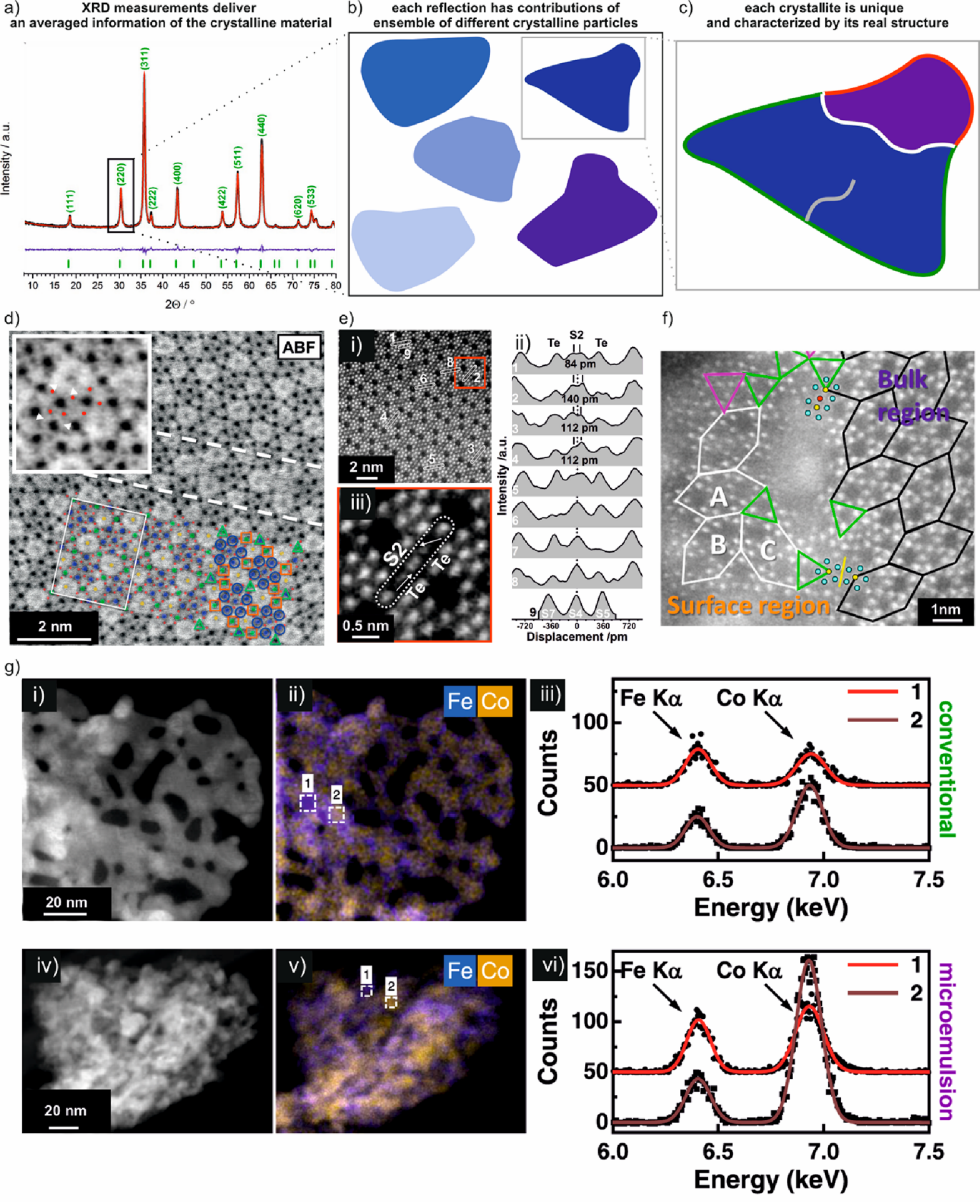
Uniqueness of a heterogeneous catalyst. (a–c) Schematic describing how integral characterization techniques average structural information from the entire catalyst ensemble, whereas individual crystallites possess local structural differences. This local information is crucial, as they influence the local chemistry and thus the formation of the active phases. (d)–(g) Examples of local structures. (a) is reproduced from ref (67). Copyright 2017 Wiley. (d) Annular bright-field (ABF) STEM image of orthorhombic (Mo,V)O_*x*_ highlighting defects in the bulk (dashed line) and polyhedral distortion (inset). Metal sites with high, intermediate, and no distortions are indicated by blue circles, orange squares, and green triangles, respectively. Mo-,V-dominating metal, channel, and oxygen sites are highlighted in blue, green, yellow, and red, respectively. The arrows in the inset denote the shift of the oxygen positions of the polyhedral. Reproduced with permission from ref (68). Copyright 2015 Wiley. (e) Displacement of the S2 sites in orthorhombic (Mo,V,Te,Nb)O_*x*_ at high resolution (i). The line profiles in (ii) correspond to the regions of interests in (i). The dotted line highlights the expected center of the S2 site. (iii) Magnified ADF-STEM image around an S2 metal site. The arrows denote the shift vectors of the Te centers with respect to the center of the hexagonal channels. Reproduced in part from ref (69). Copyright 2020 The Royal Society of Chemistry. CC BY 3.0. (f) Difference of surface versus bulk structure of orthorhombic (Mo,V)O_*x*_. Different structural motifs and orientations of the motifs in surface and bulk regions are observed as highlighted by the various tiles. Reproduced in part from ref (70). Copyright 2017 American Chemical Society. (g) STEM-EDX comparison of differently prepared Co_2_FeO_4_ samples showing nanoscale compositional inhomogeneities. (i, ii) STEM dark-field images and (ii, v) EDX maps, comparing the elemental distribution of Fe (blue) and Co (yellow) of the conventionally and microemulsion prepared Co_2_FeO_4_. The white dashed rectangles highlight 6 × 6 nm^2^ areas with increased Fe (1) or slightly increased Co (2) content with respect to the nominal atomic ratio of Co:Fe = 2. (iii, vi) EDX spectra extracted from the two regions 1 and 2 shown. The Co enrichment in the microemulsion sample is much stronger compared to the conventional Co_2_FeO_4_ sample. Reproduced in part from ref (65). Copyright 2022 American Chemical Society. For further examples we refer to Section 2.5.

Although we generally assume that we are investigating samples that are phase pure and homogeneous during heterogeneous catalysis studies, at least to the extent shown by X-ray diffraction (XRD) studies or to the microscale captured by SEM (Figure 8), this apparent homogeneity is a measure that integrates over the entire sample and underrepresents the contributions of minority and amorphous phases or defects. In the view of a catalytic process, each crystallite within a catalyst system is unique (Figure 8a–c). This uniqueness is related to a catalyst particle’s chemical composition, geometrical and electronic structure, or morphology. Examples are presented in Figure 8d–f. These qualities are then modified during the reaction due to catalyst restructuring. Restructuring is a phenomenon that occurs on multiple scales and can manifest itself through surface reconstruction, segregation, sintering, or (frustrated) phase transitions. These structures generated *in situ* may only be stable under operating conditions and need to be added to our portfolio of known metastable structures. Furthermore, transport processes, heat of reaction, or nonuniform heat (electric field) distributions lead to temperature (applied potential) and chemical potential gradients within the thermal catalytic (electrochemical) reactor and vary the appearance of catalytic particles on longer length scales.

Recent findings comparing the performance of two spinel-type Co_2_FeO_4_ catalysts toward oxygen evolution^65^ (OER) serve as another example of how two catalysts can exhibit the same average structure and composition, but local differences in the Co and Fe distribution strongly affect the activation of these samples (Figure 8g). The real electronic properties of any catalyst are triggered by the delicate interplay of the energy levels of the defects with the corresponding analogues of the ideal structure. The cumulative magnitude of these effects is strongly affected by the local chemical composition and influences the catalytic conversion as a macroscopic and averaged measure.^66^

Moreover, as opposed to single-crystal model catalysts, real industrial catalysts are extremely rich in diverse structures, which gives rise to a heterogeneity of the samples during a reaction that exists on different scales. Here, the individual contributions of, for instance, surface defects and local nonstoichiometry to activity and selectivity are not yet fully understood. These inhomogeneities can induce complex electronic effects which can modulate electron transfer in semiconducting catalysts. For instance, antisite defects can introduce a single energy level inside the forbidden zone.^71^ The frequent occurrence of antisite defects may cause an internal coupling. As a consequence, individual energy levels split according to the Pauli exclusion principle which leads to the formation of impurity bands.^71,72^ In addition, extended defects can be treated as heterostructures which can feature quantum walls or act as additional electron barriers.^73^

Hence, when we look at relevant scales for active site formation, which are the nano and atomic scales, we must consider that there is a specific fingerprint for each crystallite. This fingerprint is expressed by different, nonidentical local structures and include different atomic scale compositions and defects in the bulk phase or a surface that is structurally (electronic and chemical) and compositionally decoupled from the bulk and can vary locally. Even the smallest deviations from ideality, such as distortions in the metal–oxygen polyhedra, can often greatly affect catalytic performance. All these deviations on the nanoscale and atomic level from the ideal crystal structure are summarized under the term the “real structure” of a catalyst. This real structure affects the local chemistry of the bulk and surface, determines the formation of active sites and the precatalyst-active phase transformation, and must thus be determined to understand the important structural features in catalysis and the transformation of the precatalyst into the active component. It also means that it is impossible to determine from one selected catalyst particle whether it is relevant to the catalytic process or whether the phase found is the one with the lowest, medium, or highest activity. To meet this complexity challenge given by the real structures and to understand their individual contributions on the catalytic performance, the only reasonable approach available to us is to capture, categorize, and quantify at least a subset of this diversity.

### 2.6 Transmission Electron Microscopy and Its Role in Heterogeneous Catalysis

If various motifs exist under reaction conditions, how do we identify which motifs are participants in the catalytic process and which are spectators, especially when our tools for studying catalysts are primarily ensemble averaging methods that are not sensitive to such diversity? Particularly, the impact of minority species or trace impurities will be buried under the response of the dominant species in the system, unless they produce an exceptionally strong response. One of the distinguishing features of *operando* EM is that we can directly visualize the structural changes that arise when we adjust the chemical potential of systems (via temperature, pressure, gas/electrolyte composition, applied electric potential etc.). Therefore, we can effectively sample the emergence of unique or metastable motifs in a subset of the catalyst particles under specific conditions. Particularly, an often-neglected aspect is how dedicated *operando* SEM can also help with this issue by providing a larger field of view at the micro- to macroscale that provides an overview of transport processes and the influence of local gradients in the chemical potential.

**Figure 9:**
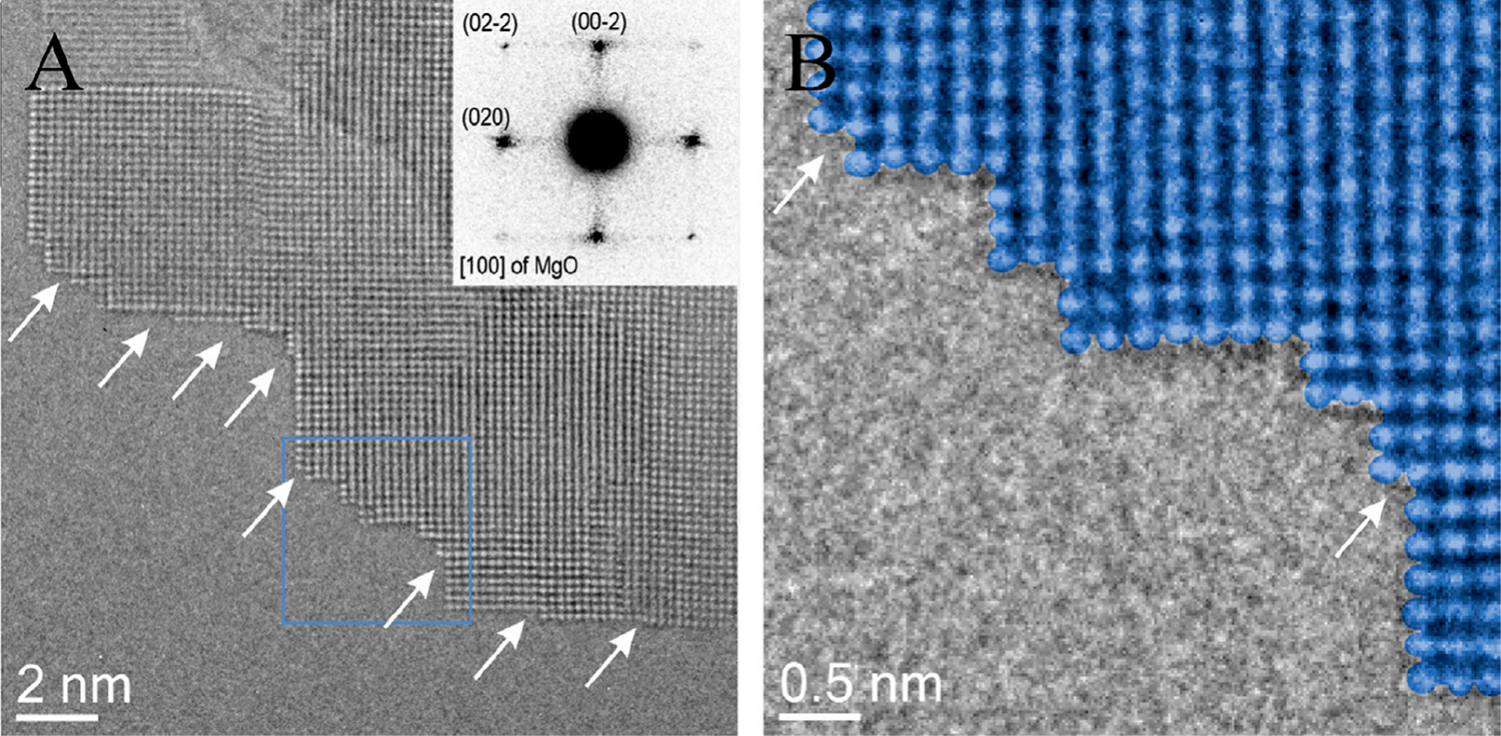
High-resolution TEM images of MgO. The inset in (A) shows a power spectrum, which allows identification of the orientation of the MgO crystal. (B) Higher magnified micrograph of (A) taken at the marked region of interest. The monatomic steps at the surface are clearly visible and marked by arrows. Reproduced with permission from ref (76). Copyright 2015 Elsevier.

However, how do we determine when complexity starts and to which degree local structures influence the catalytic performance? These questions may be answered using binary MgO nanocrystals as an example. MgO resembles one of the structurally simplest oxides with a high ionic bonding character and is composed of Mg^2+^ cations and O^2–^ anions. At ambient conditions, bulk MgO crystallizes in the thermodynamic stable rock salt structure, while small clusters can also form metastable polymorphs that have hexagonal tube-like character.^74^ This material is active in the oxidative coupling of methane (OCM) at elevated temperatures (*T* = 1073 K).^75^ Under the applied reaction conditions, MgO stabilizes electrophilic oxygen at the surface. While XRD analysis and TEM imaging (Figure 9a) suggest the absence of lattice defects in the bulk, high-resolution TEM images (Figure 9b) indicate the presence of monatomic surface steps, which have been discussed as active sites.^76^ The question whether these monatomic surface steps are already an indication for the presence of surface defects or whether they still correspond to the ideal bulk termination remains challenging to answer. A more detailed analysis of the TEM images (Figure 9b, arrows), however, suggests that surface atoms located at the monatomic steps are slightly displaced from their ideal equilibrium position, indicating that charges are slightly redistributed at the surface.

Although deviations from the ideal structure are small, this example shows that local structures and structural complexity already need to be considered even for simple binary systems. Determining the role of these small changes from the ideal is one of the key challenges on our quest to understand the working principles of heterogeneous catalysts, and clearly, EM is our primary experimental weapon on this quest.

Therefore, capturing heterogeneity at the atomic level should be an integral part of any catalyst characterization prior to *operando* experiments, even if they are based only on *ex situ* studies in vacuum. The insights obtained from these measurements are useful pieces of the puzzle, which allow us to build more realistic theoretical models for improved predictions about the performance of these multivariant systems. From the extent of heterogeneity, the degree of versatility of the working structure can be estimated, allowing a qualitative and rough estimation of the local reactivity differences. Furthermore, a detailed knowledge of the real structure and working structure also provides retrospective insight into the transformation mechanism of the precatalyst, which is important for design concepts aimed at introducing beneficial heterogeneities into the solid catalysts. Here, as a precursor to the problems that we can address with *operando* microscopy, we highlight additional examples where the impact of structural complexity on catalyst performance is elucidated by conventional TEM measurements.

#### 2.6.1 Complexity in Multinary Oxides

Nanostructured multinary bulk oxides are frequently used in catalytic applications and are applied particularly to the selective oxidation of hydrocarbons. The multinary nature of such catalysts is often a result of tuning the selectivity–activity ratio by dissolving an active or selective component in a crystalline matrix of elements with different catalytic properties. It should be noted that the synthesis of phase-pure oxides (based on Rietveld refined X-ray diffraction data) is a prerequisite for an in-depth study of their structure–function relationship. For multinary compounds, this is not a trivial task. In addition, their crystal lattices are penetrated by defects that are difficult to capture solely by XRD analysis. These defects modulate not only the geometric and electronic structures but also the catalytic performance as they alter the microstructure and surface termination.

##### 2.6.1.1 Decoupling of Bulk and Surface Structures

The surface is the most important part of any catalytic system. Small factors, such as atom displacements or monatomic steps, are essential ingredients in tuning the electronic surface structure and, thus, the catalytic performance. This tuning of the electronic surface structure is essential, for instance, for the oxygen reduction reaction (ORR) over LaMnO_3_ and was accomplished by realizing Mn^2+^/Mn^3+^ redox couple surface sites during synthesis.^77^ Density functional theory (DFT) calculations and scanning TEM–electron energy loss spectroscopy (STEM-EELS) measurements demonstrated that this situation led to La-deficient surfaces compared to the bulk, and the La vacancies at the surface were filled by Mn^2+^ cations.^77^ Furthermore, the electronic surface state of perovskites can be adjusted by the applied reaction conditions, while staying compositionally decoupled from the bulk.^78^ In addition, a disordered transport layer can exist in multinary oxides that separate the bulk and surface and regulate the oxygen exchange.^79^ This self-regulating layer is tunable, and the addition of water steam to the feed can crucially alter the structure of the surface and hence change the selectivity distribution toward the desired product.^80^

**Figure 10:**
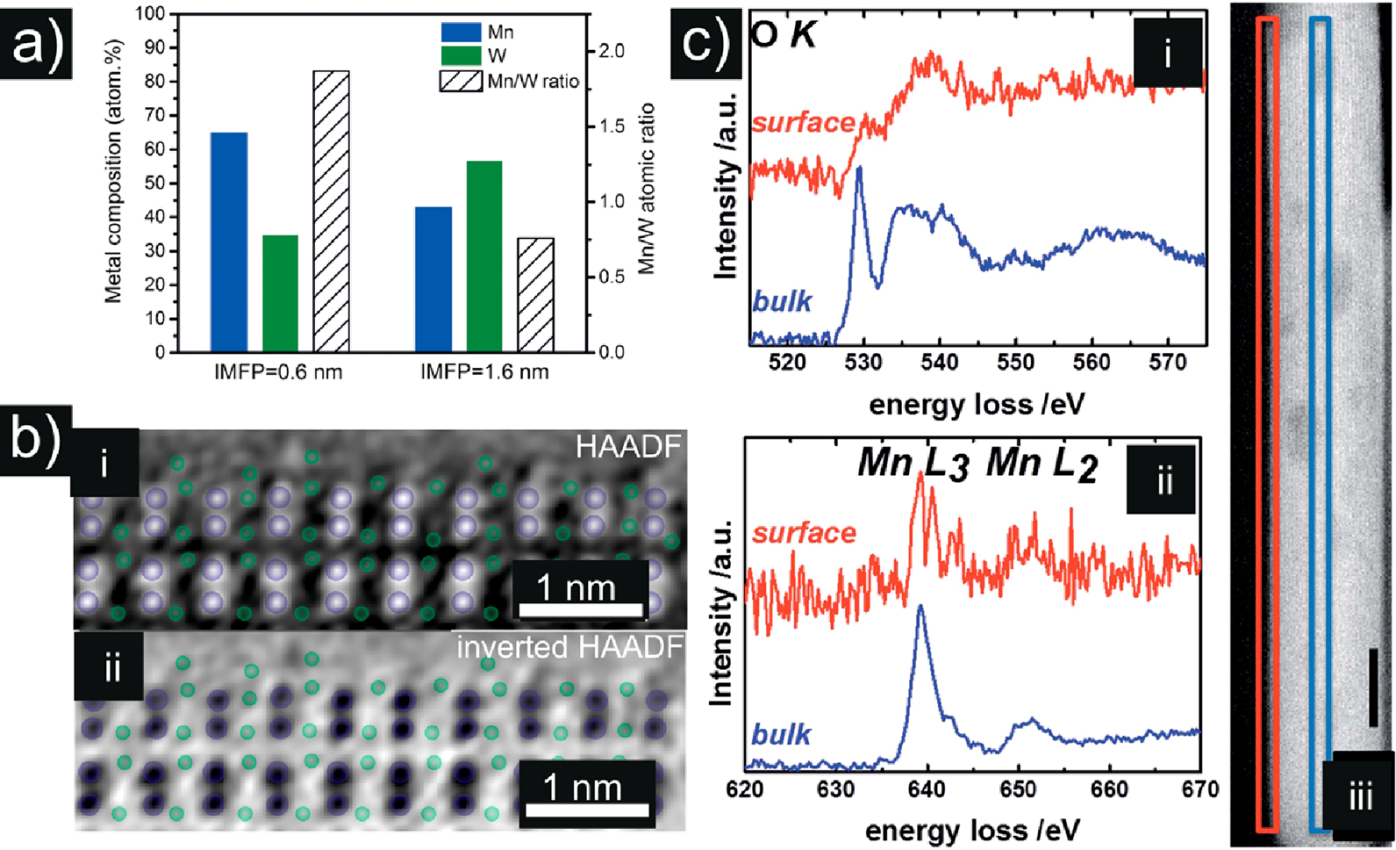
Complementary NAP-XPS and EM analysis of MnWO_4_. (a) Depth profile of the elemental composition of MnWO_4_ nanorods in terms of the inelastic mean free path (IMFP) of electrons measured by synchrotron-based NAP-XPS at *T* = 300 °C applying a total pressure of 0.25 mbar O_2_ and He at flows of 2 and 2.2 mL min^–1^, respectively. Reproduced in part with permission from ref (95). Copyright 2016 Wiley. (b) Surface termination of the *b* plane viewed along the growth direction [001] by FFT-filtered atomic-resolution STEM images. (i) HAADF and (ii) inverted HAADF image. Mn, green; W, violet. Reproduced in part with permission from ref (95). Copyright 2016 Wiley. (c) STEM-EELS measurements of the surface (red) and bulk (blue) of MnWO_4_ showing (i) the O K- and (ii) the Mn L-edges. The squares in the STEM image of MnWO_4_ in (iii) indicate the region where EELS measurements were conducted. Red: surface; blue: bulk. The black scale bar in (iii) is 10 nm. Reproduced in part from ref (96). Copyright 2019 Royal Society of Chemistry. CC BY.

For oxides, a decoupled surface can be realized by site isolation. Site isolation has been identified as one of the ingredients in the seven pillars that define a selective oxidation catalyst.^81^ For selective vanadium-containing multinary oxides,^79,82−84^ for instance, surface-sensitive integral *in situ* experiments have unraveled two-dimensional (2D) oxide layers of vanadium oxides that terminate the bulk structure and differ in composition and electronic states from their bulk analogues (Figure 10a).^80,85−93^ This decoupling induces a gas-phase-dependent charge transfer from the bulk to the surface and dynamically modulates the work function, electron affinity, and surface potential barriers,^87,94^ which was not observed for less selective V_2_O_5_.^87^ Motivated by the concept of site isolation, manganese oxides that are usually total combustion catalysts in the oxidation of propane were converted into selective dehydrogenation catalysts by dissolving their atomic building units in a matrix of tungsten oxide, i.e., MnWO_4_.^95^ High-resolution HAADF-STEM surface imaging revealed the presence of a Mn termination layer that resembles chains of Mn_2_O_*y*_ dimers. These dimers are distorted compared to their bulk analogues (Figure 10b). Spatially resolved STEM-EELS measurements disclosed an additional compositional and electronic decoupling of the surface (Figure 10c).^96^ This example shows that knowledge-based synthesis combined with integral and local structural analysis can lead to successful tailoring of the precatalysts. However, to which extent isolated sites contribute to the catalytic conversion remains unclear, and its answer is complicated by the fact that they are one design feature out of seven^81^ (lattice oxygen, metal–oxygen bond strength, host structure, redox, multifunctionality of active sites, site isolation, and phase cooperation). It can only be answered if their dynamic interplay under reaction conditions with the other six pillars is understood.

Metal-oxide-based catalysts are similarly important for electrocatalysis applications, especially for facilitating the rate-limiting oxygen evolution half-cell reaction in water splitting. Ir and Ru oxides are benchmark catalysts for this reaction under acidic conditions,^97,98^ whereas transition metal oxides,^99,100^ especially those based on Ni and Co,^101^ are widely explored as earth-abundant, low-cost alternatives for alkaline water electrolysis. Multinary oxides, especially those where Fe is added to Ni- or Co-based oxides/hydroxides, have become interesting candidates for tailored design. In this case, it is well-established that trace Fe addition strongly improves the activity of these catalysts.^102^ There is also increasing evidence that compositional inhomogeneities and surface effects play an important role in determining the properties of these electrocatalysts. For example, it was found that nominally identical spinel Co_2_FeO_4_ catalysts prepared using two related synthesis methods, conventional coprecipitation versus microemulsion-assisted coprecipitation, exhibited different performance, where the latter possessed both higher activity and stability.^65^ Here, STEM-EDX mapping (Figure 8g) in combination with analysis with complementary *quasi in situ* X-ray photoelectron spectroscopy and *operando* X-ray adsorption spectroscopy revealed the presence of nanoscale Co^2+^-rich domains, accompanied by reducible Co^3+^ sites in the microemulsion prepared sample after reaction, features that are absent in the conventionally prepared samples.

**Figure 11:**
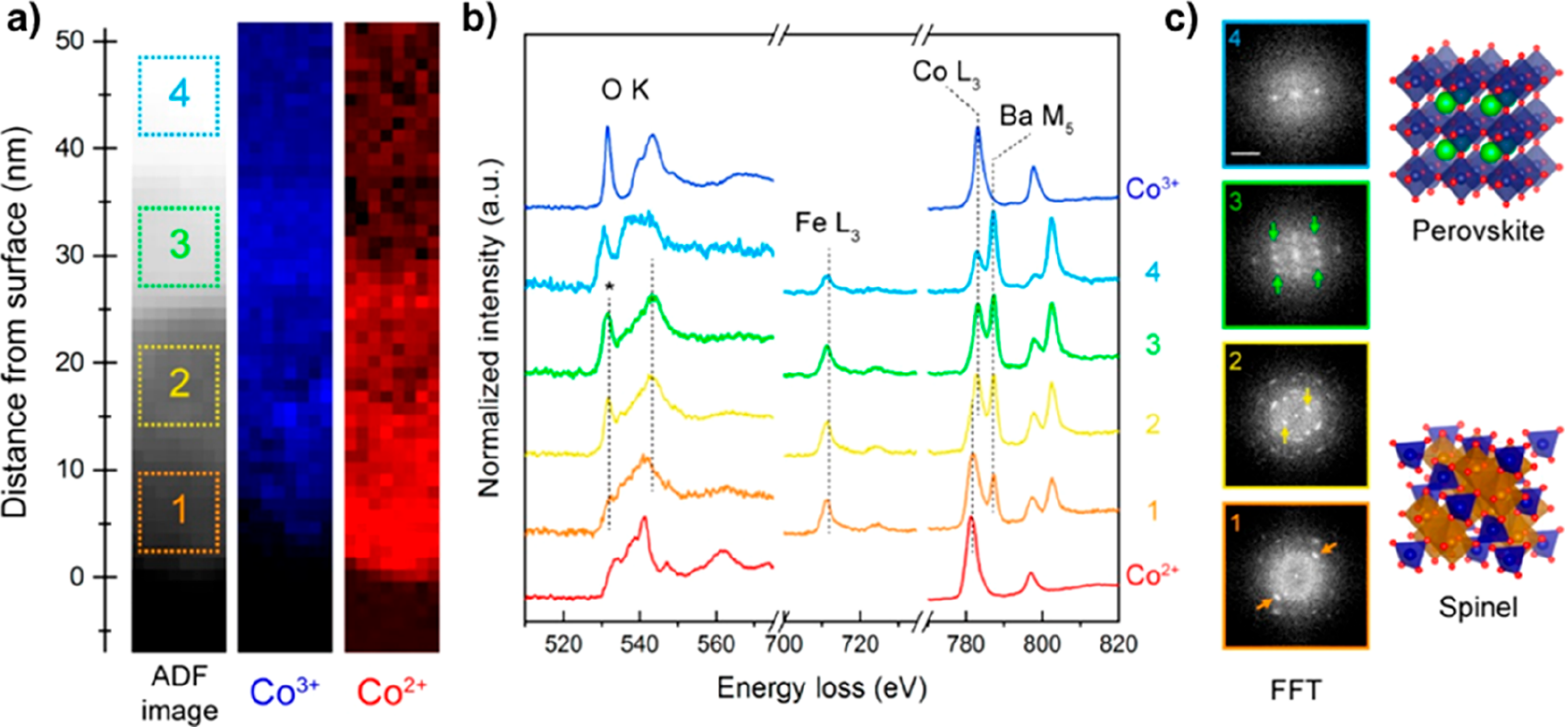
EELS and electron diffraction analysis of the BSCF surface revealing differences between the perovskite particle bulk and surface after KOH immersion for 3 h. (a) ADF image close to the BSCF surface and the corresponding MLLS fitting maps of Co^2+^ and Co^3+^. (b) EEL spectra of O K, Fe L_3,2_, Co L_3,2_, and Ba M_5,4_ edges with respect to the 4 subregions of interest in (a). CoO (Co^2+^) and LiCoO_2_ (Co^3+^) reference EEL spectra for MLLS fitting are also included. (c) Selected area FFTs with respect to the 4 subregions indicated on the ADF image. The green, yellow, and orange arrows indicate the reflections {113}, {111}, and {400} of the Co/Fe spinel structure, respectively (scale bar is 5 nm^–1^). Reproduced from ref (105). Copyright 2020 American Chemical Society.

Furthermore, perovskites have also been found to undergo surface structural transformations during electrocatalysis, such as amorphorization,^103,104^ which are robust enough to be identified using post-mortem TEM. Recently, the local structural and chemical changes in a complex Ba_0.5_Sr_0.5_Co_0.8_Fe_0.2_O_3−δ_ (BSCF) perovskite were investigated in greater detail with various TEM techniques, including STEM-EELS and identical location comparisons.^105^ These analyses reveal that the surface of the as-synthesized particles adopts a spinel-like structure that is Co- and Fe-rich upon immersion in the alkaline electrolyte where the surface Co ions have a reduced valence of 2+ compared to 3+ in the bulk. Post-mortem STEM-EELS (Figure 11) further revealed that the Co^2+^/Fe^3+^ oxidation state of the spinel surface is not altered during electrochemistry.

##### 2.6.1.2 Defect Chemistry and Their Associated Geometries

It is often tempting to believe that observations made from a small snapshot of the sample are representative for the entire ensemble of NPs. However, such complex multinary oxides exhibit a huge variety of synthesis inherent inhomogeneities that become apparent on the atomic scale.^106,107^ Using phase-pure orthorhombic and p-type semiconducting^108^ (Mo,V)O_*x*_ as a structural example, a quantitative defect analysis has been made by high-resolution HAADF-STEM imaging.^70^ In this study, 19 different structures (Table 1) were identified using the concept of tiling. The individual structures occur with different probabilities resulting in a STEM derived defect density of 3.3%.

**Table 1:**
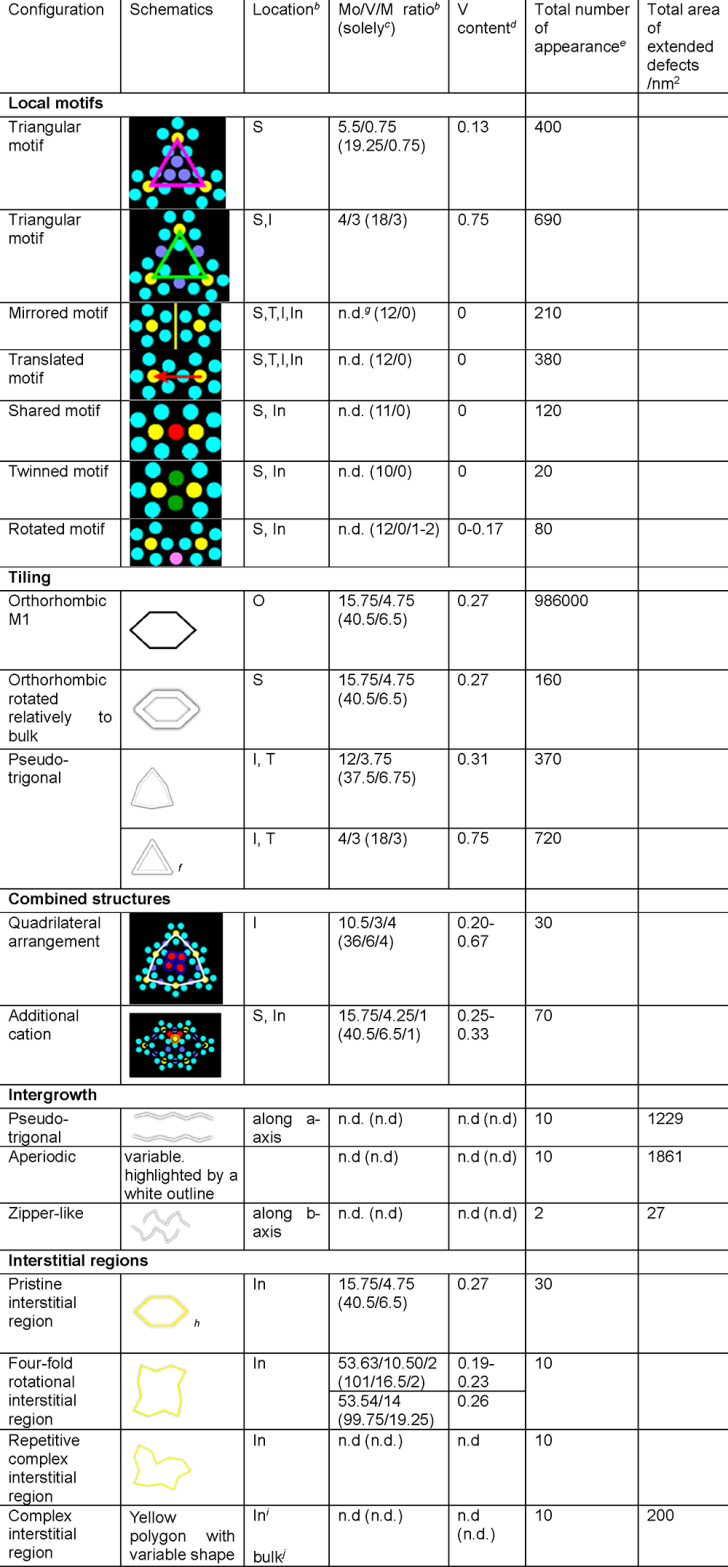
Catalog of Observed Structures and Their Compositions (Reproduced from Ref (109). Copyright 2020 American Chemical Society)

a S – surface region, I – intergrowth, T – trigonal phase, In – interstitial region, O – orthorhombic phase.

b Nominal content of all cations in the structure. M corresponds to unidentified sites. Here, Mo and V are not distinguishable.

c Nominal content taking the embedment of the local structure into the crystal into account, i.e., shared atoms at edges and vertexes. M corresponds to unidentified sites. Here, Mo and V are not distinguishable.

d V content normalized to Mo; the interval considers the borders between full occupancies of Mo or V of the unidentified sites.

e Counted number of appearances. The rounded values account for the error of counting and/or the uncertainty when the structure was not clear enough for counting.

f There is no difference between the triangular motif and the trigonal tile. Different coloring was only used to separate surface and intergrowth motifs.

g Not distinguishable.

h Adjacent to both orthorhombic bulk tiles A and B.

i Discontinues the pseudotrigonal intergrowth.

j Interstitial-like region, surrounded only by the bulk M1 structure and containing at least one motif.

The bulk structure can also fluctuate locally. Besides extended defects, distortions in the oxygen sublattice have been observed by annular bright-field (ABF) STEM imaging that directly affects metal–oxygen octahedra (Figure 8d).^68^ Not only the oxygen sublattice that appears distorted or displaced but also individual metal sites can be shifted from the ideal equilibrium position. For (Mo,V,Te,Nb)O_*x*_ such kinds of displacements were observed for metal sites that connect the hexagonal channels (Figure 8e).^69^ The metal centers are shifted toward the channel sites. The observed displacements add strain to the structure and give rise to the formation of local dipoles, site-specific stress, and strain that may locally change the potential energy.

#### 2.6.2 Complexity in Metal Nanoparticle Catalysts

**Figure 12:**
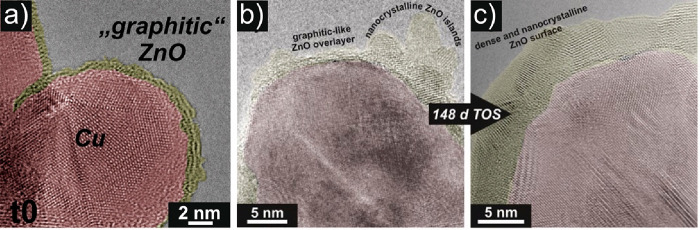
TEM investigation of SMSI states in heterogeneous catalysis. (a) Graphitic ZnO is decorating the surface of a Cu nanoparticle after reductive activation at 250 °C of industrially relevant Cu/ZnO/Al_2_O_3_. Reproduced in part with permission from ref (117). Copyright 2015 Wiley. (b) and (c) Representative TEM images after exposing the Cu/ZnO/Al_2_O_3_ to reaction conditions (230 °C, 60 bar, CO_2_/H_2_ mixture) and after 148 days time on stream (TOS), respectively. Reproduced in part with permission from ref (39). Copyright 2016 Wiley. In (a) to (c), Cu NPs are highlighted in red, and ZnO moieties are colored yellow.

Metal NPs supported on oxides are key catalyst systems in the chemical industry. At first glance, their description seems simple. Metallic NPs of a certain size and shape are homogeneously distributed on an oxide support, and the catalytic event takes place at their perimeter. Looking more closely at the situation, one finds that the particle size distribution of the metal NPs exhibits a certain dispersion.^110,111^ Although correlations to the macroscopic catalytic performance area aimed to be worked out from these data, a statement as to which size fraction within the dispersion is now the most representative can thus only be predicted to a limited extent. This is complicated by the fact that metal NPs also exhibit a certain shape distribution, if not fully equilibrated, which leads to the exposure of different crystallographic lattice planes and an unequal number of defects. This heterogeneity means that findings on structure-selective reactions must necessarily be subject to precise particle size and shape control. Many reactions occur on reducible oxidic supports, so that after reductive activation the support can embed the metal NPs, as seen during *ex situ* and *in situ* studies. This is the so-called strong metal support interaction (SMSI) effect.^112−116^ Examples of SMSI effects in catalysis are presented in Figure 12. This embedment of the NPs with the oxidic component often appears complete in 2D projected TEM images and would be in stark contrast to the perimeter effect described above, as no fraction of the NP is in contact with the gaseous atmosphere. Moreover, the oxidic overlayer is often characterized by different thicknesses and structures.

These examples highlight a small selection of the complexity we must face during our endeavor of correlating structures to activity. They also emphasize the importance of measuring conversion during the analysis in order to at least ensure a relevant chemical potential.

### 2.7 Seeing Active Sites?

Can we image the structure of active sites with the TEM? Based on the arguments we have presented so far, it should be clear that the answer is “no”. We cannot resolve the active sites of a catalyst that exist in a dynamic state with conventional TEM, regardless of the spatial resolution. Even with *operando* TEM, identifying active sites is still impossible at the moment because of the time resolution of our current instruments (microscope and mass spectrometers for reactivity analysis) and our inability to visualize the conversion of molecules on the catalyst surface.

The second point regarding capturing conversion exists as a deeper conceptual question with *operando* EM experiments. While we can image individual particles and their changes with very high spatial resolution, especially in the gas phase, we cannot determine whether those particles are participating in the catalysis because we do not capture at the same time the reactant and product molecules in the images. Even if we could get such data, they will likely be ensemble-averaged over large probe regions, which masks the real performance of specific locally resolved structures. This is important because in some systems less than 1% of the entire accessible surface is active (see ref (80) for calculations, which uses data from refs (118) and (119)). Since the particles selected for the *operando* EM investigation also exist within a larger and often inhomogeneous ensemble of catalyst particles, they create a unique conundrum regarding the significance of these studies compared to other *operando* techniques. Are we truly looking at the morphology of a working catalyst in our experiments? Does the particle we have selected to observe contribute to the overall performance? The limited statistics we typically have in *operando* EM experiments mean that it is not straightforward to establish whether the observations are relevant to the catalytic process and how the behavior of these few particles extrapolates to the behavior of the overall ensemble. Even if we can confirm that the particles are participating in the reaction and we are observing relevant catalyst behavior, we still need to determine whether those dynamics contribute to activation or deactivation!

These limitations of *operando* EM studies can be better understood using an example from gas-phase thermal catalysts where gas-phase holders have already been coupled to an online mass spectrometer. The periodic oscillations in conversion found during CO oxidation with noble metal catalysts^8^ have been a frequent subject of such EM studies where the repetitive restructuring of the catalysts is correlated with these oscillations.^53,120,121^ Yet, despite these studies, several key questions remain open since we cannot visualize the gaseous reactants, short-lived intermediates, and products. These outstanding questions include whether the structural changes are initiated by the conversion of gas molecules or if the restructuring is simply a response to changes in the gas composition and how a possible cooperative response between disconnected particles is developed.^122^

Conversely, we highlight here that the spatiotemporal resolution of today’s *operando* EM experiments allows for the detection of the formation of possible working phases or adsorption sites as well as aging processes, which are still important for understanding catalytic processes. The critical point here is that the structural information obtained from *operando* experiments must be acquired with sufficient statistics to allow for meaningful correlation to their catalytic performance, which can then be used to improve the theoretical models for elucidating the active sites of the catalysts.

## 3 *Operando* Electron Microscopy Studies of Heterogeneous Catalysts

In this section, we will discuss in general the concepts behind *in situ*/*operando* setups and then present EM setups and examples that allow the detection of catalytic conversion and related changes during the investigation of thermal gas-phase catalysis or electrocatalysis inside the electron microscope, which is the prerequisite of *operando* experiments and examples for the state-of-the-art of such studies.

### 3.1 Milestones in the Development of EM for *In Situ* Imaging in Liquids and Gases

Here, our discussions will be limited to the two most common forms of EMs, SEM and TEM. First, we start with a brief introduction to the working principles of these EMs (see Figure 1 for the common imaging modalities for SEM and TEM), followed by how they have been adapted for imaging in liquids and gases over the years.

In SEM, electrons are accelerated to a few keVs energy (typically between 1 and 30 kV) and focused into a fine probe using electromagnetic lenses. An image is then formed by scanning this beam over the surface of the sample and collecting the signals generated due to interactions between the primary beam and the sample using different detectors. Typical signals used for forming the image include secondary electrons (SEs) that provide topological information, backscattered electrons (BSEs) that provide compositional information, and characteristic X-rays that enable compositional analysis (EDX) (Figure 1b). For further details see ref (32).

In TEM, electrons are accelerated to a few hundred keVs (usually 200–300 kV for solid catalysts) and used to probe thin specimens (the optimal thickness is in the range of a few hundred nanometers or less). In general, the electrons used to form the image are those that have passed through the sample. TEM can also be operated in two modes, a conventional broad beam mode and a focused probe mode similar to the SEM (commonly known as STEM). By using different apertures or filters to select the type of electron that contributes to the image signal, one can choose between different contrast modes that highlight certain features of the samples which are commonly phase contrast, diffraction contrast, and *Z*-contrast.^33^ We can also perform chemical spectroscopy using EDX^33^ or EELS^33,123^ (Figure 1a).

It was already realized in the early days of EM development^124^ that samples can be denaturized when viewed in the vacuum of the electron microscope.^125^ Therefore, ways to study samples in air at elevated pressures and even under wet conditions have already been sought ever since the EM was invented. Note that the approaches used today remain conceptually similar to the main concepts developed back then. For example, TEM similar to modern environmental TEM that allowed for the introduction of different gases (air, hydrogen and chlorine) and pressures up to approximately 200 mbar was already developed by Ernst Ruska, the inventor of the TEM, in 1942.^126^ This system was used to observe the conversion of colloidal Ag to AgCl when 6.7 mbar chlorine gas was introduced into the microscope (Figures 12a and 12b). Such systems are commonly known as open-cell systems.

**Figure 13:**
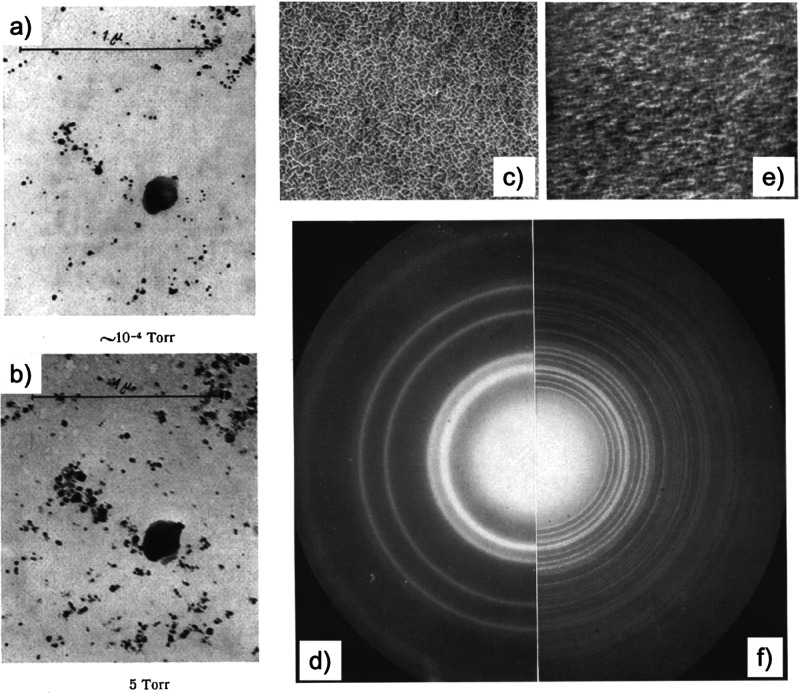
Early attempts at *in situ* TEM. Images (a) and (b) represent experiments in which Ag particles are exposed to a chlorine environment using the open-cell approach. The formation of AgCl can be observed in (b). Reproduced with permission from ref (126). Copyright 1942 Springer Nature. Experimental observations of the transformation of Ag to Ag_2_S in the presence of H_2_S gas are depicted in (c)–(f). (c) and (d) denote a TEM micrograph and SAED pattern of pristine Ag, respectively. (e) and (f) show a TEM image and the corresponding SAED pattern after the transformation to Ag_2_S, respectively. Reproduced with permission from ref (129). Copyright 1942 Springer Nature.

The second approach, generally known as closed-cell systems, involved encapsulating samples in liquids or gases between impermeable membranes. Such systems for observing liquid samples were developed as early as 1944 using 20 nm thick thin carbon films.^127,128^ These carbon sheets formed a dense chamber where the gas pressure could be raised up to approximately 900 mbar. After exposing Ag particles to a H_2_S:O_2_ = 3:1 gas mixture, the formation of Ag_2_S followed at room temperature (Figures 13c–13f). The solid-state chemistry was accompanied by a high mobility of the Ag particles, and the reaction stopped after 3–4 min. It was also found that the transformation toward Ag_2_S was heavily stimulated by the electron beam, as Ag_2_S was only observed at positions that have been exposed to the electron beam. In addition, it is known that under normal conditions Ag_2_S is only formed at about 100 °C and not at room temperature. The author concluded that the electron beam thermally initiated and accelerated the reaction—a first hint that *in situ* generated reactive radical species can severely influence the outcome of the observation.

**Figure 14:**
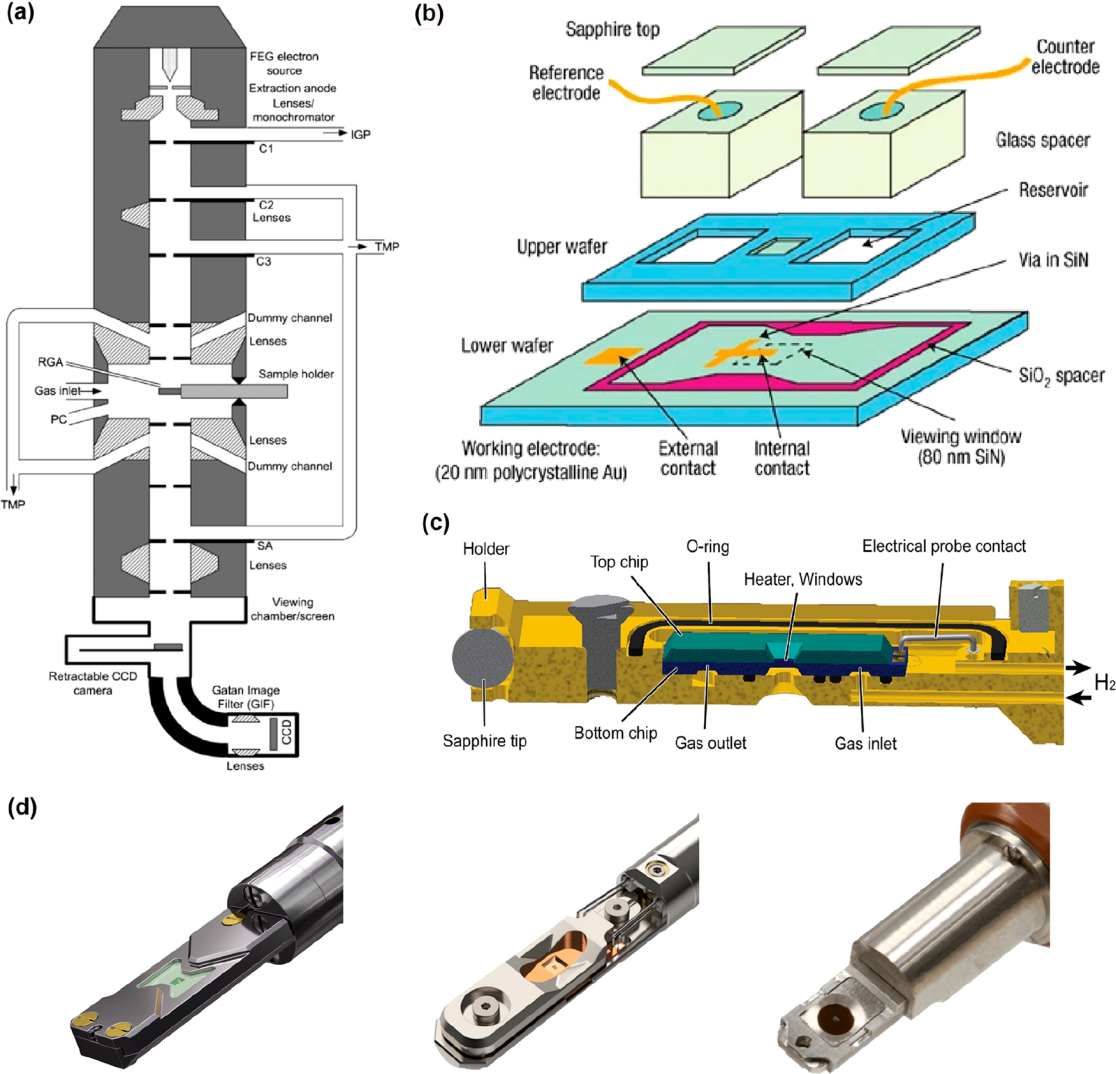
Historical developments in open- and closed-cell electron microscopy systems and contemporary *operando* TEM holders. (a) Schematic of a commercial environmental TEM. Reproduced with permission from ref (143). Copyright 2010 Taylor & Francis. (b) Schematic of the MEMS-based liquid electrochemical cell from Williamson et al. Reproduced with permission from ref (131). Copyright 2003 Springer Nature. (c) Schematic of an early MEMS gas holder system. Reproduced with permission from ref (144). Copyright 2008 Elsevier. (d) Images of contemporary *in situ* holders we have at the Fritz Haber Institute of the Max Planck Society. From left to right: A Protochips Atmosphere holder, a DENSsolutions Climate holder, and a Hummingbird Scientific bulk electrochemistry holder. Images courtesy of the respective holder manufacturers.

Based on these early experiments, a new subfield of EM for heterogeneous reactions under *operando* conditions developed over time. Modern open-cell TEMs and SEMs (Figure 14a) add differentially pumped apertures and vacuum pumps to the microscope column to allow higher gas pressures around the objective lens of the microscope.^126,130^ These modified EMs are also called environmental SEMs or TEMs (ESEM or ETEM). The major breakthrough for closed-cell systems (Figure 14b,c) came about when it was demonstrated that silicon nitride thin films microfabricated on silicon wafers with thicknesses thin enough for the energetic electrons to pass through are viable as encapsulating membranes^131^ (Figure 14b). Since these reaction cells are manufactured by standard semiconductor processes, they can be mass produced reliably in large quantities, and this concept opened the door to commercialization of the technology. Currently, most liquid and gas cells consist of two silicon supports (also known as “chips”) with etched windows that have a continuous silicon nitride film on top (commonly 50 nm thick).^29^ In most implementations, this sandwich of chips is then hermetically sealed in custom-built EM holders (Figure 14d) to isolate the gas or liquid environment. Other critical developments in the technology include the use of rubber O-rings for sealing of the reaction cells in the holders,^132^ the fabrication of thin-film electrodes for electrochemistry,^131,133,134^ or heating coils for sample heating,^23,135^ made through lithographic processing and the inclusion of tubing for gas^23^ and liquid^136^ flow. Currently, at least three commercial vendors provide such liquid and gas cell solutions. One can also make their own home-built system if one has access to precision machining and microfabrication capabilities. For liquid-phase studies, experiments can alternatively be performed in closed cells based on graphene and other 2D materials,^137^ where the ultrathin membranes and miniscule liquid pockets allow high-resolution imaging of chemical reactions under the electron beam. While such systems cannot be used for electrochemistry yet, there has been promising progress in the manufacture of multimembrane stacks for liquid mixing.^138^

In general, open-cell systems are only suitable for reactions involving gas pressures up to a few hundred millibars, whereas closed-cell systems are suitable for reactions involving gas pressures of 1–2 bar or liquid electrolytes. It is also possible to perform electrochemistry experiments using an open-cell approach without membrane enclosure,^139^ but the electrolyte is limited to low vapor pressure liquids, and since electrocatalytic systems often involve aqueous electrolytes, this approach is usually not employed for such studies. Currently, the closed-cell approach is more widely adopted for environmental EM because it does not require a specialized microscope. While the vast majority of *in situ*/*operando* experiments have been conducted in TEMs so far, there are fledging efforts to develop reaction cells for the SEM and exploit the flexibility that is afforded by its larger chamber size.^54,140−142^ We summarize the pros and cons of the commonly available *operando* systems for catalyst research in Table 2.

**Table 2:** Summary of Relative Strength and Limitations of the Most Important *Operando* EM Techniques

Technique	Relative Strengths	Limitations
ETEM	•High spatial resolution (subnanometer)	•Limited liquid-phase options
	•Moderately high temporal resolution (subsecond)	•Low pressure
	•Spectroscopy possible	•Beam damage
	•High temperature	
	•Conversion detection possible	
ESEM	•Transport process and macroscale phenomena	•Low pressure
	•Moderate spatial resolution (nanometers)	•Low temporal resolution (tens of seconds)
	•High temperature	
	•Conversion detection possible	
Closed cell		
Gas phase	•Ambient pressure	•Beam damage
	•High spatial resolution (subnanometer)	
	•Moderately high temporal resolution (subsecond)	
	•Spectroscopy possible	
	•High temperature	
	•Conversion detection possible	
Liquid phase	•Moderate spatial resolution (nanometer)	•Beam damage (very low electron fluxes and doses required)
	•Moderately high temporal resolution (subsecond)	•Limited spectroscopy options
	•Relevant environment of electrolyte and applied potentials	•No conversion detection
Quasi *in situ*		
Quasi *in situ* TEM (gas)	•Atomic resolution	•Possible artifact formation during cooling and change of environment
	•Spectroscopy possible	•No real-time information
	•Flexible devices for different application	
	•Only “limited” beam effect	
	•Conversion detection possible	

### 3.2 *In Situ* and *Operando*: What is the Difference?

Next, we address the question when is an EM experiment *in situ* and when is it *operando*? Here, we refer to the definition set out by Bañares for *operando* spectroscopy where “*Operando* spectroscopy is a methodology that combines the spectroscopic characterization of a catalytic material during reaction with the simultaneous measurement of catalytic activity/selectivity”.^26^ We specifically emphasize the need for simultaneous measurement of catalytic activity/selectivity because so far the term *operando* has been somewhat loosely used in EM experiments, where it had also been applied to experiments where the catalytic activity/selectivity were determined from the same samples in different reactors under supposedly identical conditions. An argument against this approach is that it is unlikely for the experimental conditions to be truly identical in the different setups as we had discussed earlier.

In electrocatalytic studies with liquid-phase EM holders (also known as electrochemical cell EM (EC-EM)) where electrochemical data, such as the current or the voltage, are concurrently acquired during the experiments, the line between *in situ* and *operando* studies tends to be more blurred. Although we can in principle obtain insight into the characteristics of the electrocatalysts from the concurrently acquired electrochemical measurements,^145,146^ it is not always clear that the microscopy data are meaningfully connected to the electrochemistry data given that the catalyst population we observe is again much smaller compared to the ensemble generating the response. So far, there has been a lack of established protocols that allow us to translate the restructuring we observe during electrochemistry into their responses in the voltage or current data. The path forward toward online product detection, which is required for selectivity determination, is even more difficult for such systems. At present, truly *operando* EC-TEM experiments can only be implemented for electrochemical reactions where one product is obtained, for instance, the OER. Addressing these issues warrants an extended discussion, and so we hold off on it until Section 3.4.3.

### 3.3 *Operando* Gas-Phase Thermal Catalysis Studies

**Figure 15:**
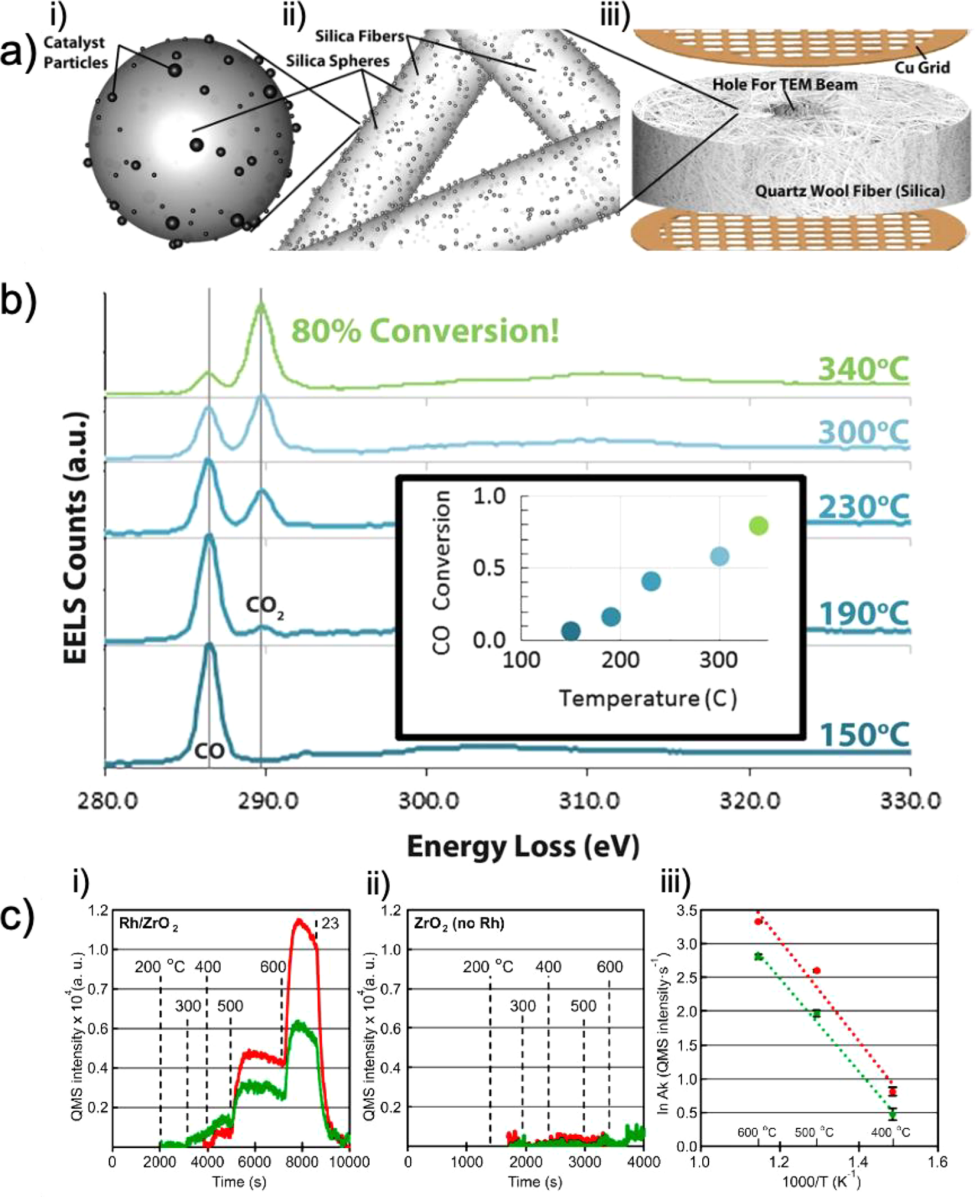
Detection of the catalytic function in modern open-cell TEMs. (a) Nonwoven silica fibers act as catalyst support and are sandwiched between two TEM grids. The hole in the center is used for imaging and spectroscopy. Reproduced with permission from ref (156). Copyright 2014 Oxford University Press. (b) Catalytic conversion during the CO oxidation on Ru NPs following the approach described in (a). Reproduced with permission from ref (156). Copyright 2014 Oxford University Press. (c) Catalytic data as obtained during HVTEM open-cell experiments during NO reduction on Rh/ZrO_2_ (i), reference measurements of the support (ii), and the corresponding Arrhenius plots (iii). Green: N_2_ production. Red: NO conversion. Reproduced with permission from ref (160). Copyright 2021 Elsevier.

In this section we focus on developments that enabled the detection of the catalytic conversion during thermal gas-phase reactions. In the gas phase, EM enables the tracking of atomic surface structures and their temporal changes (Figure 15).^23^ However, it will remain a mystery even with proof of function that these changes are part of the catalytic cycle or rather belong to a stoichiometric reaction of the solid with the local gas-phase composition. This is due to the difficulty in proving at the atomic level which change is from the catalytic reaction and which is from the inelastic interaction, i.e., radical chemistry, between the electron beam, gas phase, and solid. Furthermore, extrapolation of results from one particle to a billion-particle ensemble in the flow reactor will not always be possible because reactor gradients cannot be studied easily. The outcome from a catalytic test in the flow reactor is an integration over all catalyst particles. For these reasons, the greatest value of *operando* EM of heterogeneous gas-phase catalysts lies not in determining active sites or describing surface structures in detail but in describing working structures, morphological and structural changes, frustrated phase transitions, and testing for their reversibility, i.e., the presence of hysteresis, exsolution of nanoparticles, or evolution of strong metal–support interactions.

In principle, almost all catalytic gas-phase reactions can be observed in closed-cell systems, provided that the experiments follow local safety regulations and do not risk damage to the microscope and holder. For example, one needs to be very careful with reactions that either work with corrosive reactants or produce corrosive products such as Deacon reactions for the catalytic production of chlorine from oxygen and HCl gas. In addition, liquids can be dosed in the form of vapors^147^ and so one can also perform experiments looking at reactions such as methanol oxidation.^148^ The most pertinent consideration in *operando* gas-phase experiments is the pressure gap,^149^ particularly for reactions that need to performed at high pressures to obtain reasonable yields, such as the hydrogenation of CO_2_ to methanol (usually at 20–60 bar) and the Haber Bosch process for ammonia synthesis (∼200 bar). Here, one can adopt either of two approaches. The first is to carry out measurements in fixed-bed reactors at the atmospheric pressures found in the gas-phase holders to directly compare the catalytic data. The second is to perform control *ex situ* experiments to determine if a pressure gap exists and whether one can reasonably extrapolate the results of the kinetic model at atmospheric pressures.

In this aspect, quasi *in situ* TEM approaches combined with the imaging of identical locations are also a promising alternative to both check on the effect of the working pressure and to determine the influence of the electron beam during functional testing. Although the temporal resolution is lacking, catalysis-induced changes of identical particles can be imaged with maximum resolution inside the vacuum of the TEM. The reaction can be conducted in a dedicated TEM grid reactor at ambient^150^ and high pressure.^151^ The possibility of using this approach to measure catalytic turnover from a TEM grid has recently been demonstrated.^150^ Using identical particles, the morphological change of Pt particles during CO oxidation could be tracked, and the same morphological changes compared to the *operando* TEM experiment have been observed.

In terms of function determination, it should be emphasized here that the amount of sample for TEM studies is in the low to mid μg range, which complicates any online measurement of catalytic conversion.^46^ In addition, due to the low amount of catalyst, it is difficult to set relevant space–time velocities. The measurement of catalytic conversion is, however, essential for catalytic gas-phase reactions since the smallest changes in total pressure, flow, temperature, partial pressure, or the presence of the electron beam can change the structure of a catalyst in a way that it becomes irrelevant for the catalytic process.

#### 3.3.1 Environmental Transmission Electron Microscopes

The development of ETEM for the study of gas-phase processes interacting with solid-state catalysts advanced significantly in the late 1990s.^152,153^ In these progenitors of the commercial ETEM, changes of a solid in a gas atmosphere can be investigated in the pressure range up to 30 mbar,^154^ although function determination was largely absent in most reports. Nonetheless, the possibilities to circumvent this dilemma have already been considered. It was shown that one can use EELS to follow the progress of a catalytic reaction^47,155−157^ (CO oxidation on Ru and Pt NPs). Combined with residual gas analysis, the progress of the reaction could be tracked while simultaneously monitoring changes in the catalyst with atomic resolution. EELS analysis of the gas phase also allowed quantification of the conversion. The same authors further developed a so-called *operando* pellet, which consists of interwoven silica fibers that can be impregnated with catalyst particles (Figure 15a and 15b). This serves to increase the amount of catalyst in the TEM and to simplify the reaction product measurement. The center of the pellet is hollow, so that catalyst particles can be imaged and examined at the edge. Using this approach, the same authors showed that RuO_2_ formed on Ru during CO oxidation acts as a spectator rather than active species as widely believed.^157^

As mentioned above, the upper pressure limit is about 0.3 mbar in conventional ETEMs. The situation changes when electrons with an energy of >1 MeV are used.^158^ With such high voltage TEMs (HVTEMs), lattice planes can be imaged even in an environment of 100 mbar of nitrogen.^159^ Combined with a mass spectrometer, it allows the detection of combustion products and catalytic conversion. For example, the concept was demonstrated using the combustion of carbon nanotubes (CNTs) and the oxidation of Rh NPs in 0.15 mbar O_2_ gas.^159^ Building on this preliminary work, the same authors also pursued NO reduction on Rh NPs,^160^ where the sample was impregnated onto a heating coil. They found that the dynamic formation of a metastable oxide surface film regulated the oxygen content on the catalytic NPs during NO decomposition at 0.3 mbar. From the MS data, they were able to extract an apparent activation energy of 62.2 and 57.1 kJ/mol for NO decomposition and N_2_ formation, respectively (Figure 15c). It also shows the influence of the catalyst on the reaction, as the thermal noncatalytic NO decomposition has an activation energy of 266.9 kJ/mol. It is these catalytic data that allow a comparison with real catalytic systems.

#### 3.3.2 Closed-Cell Systems

As early as the 1960s, work was carried out at the Fritz Haber Institute of the Max Planck Society on thin metal foils which, on one hand, were transparent to the electron beam but on the other hand were stable enough to decouple a 1 bar gas atmosphere from the vacuum of the TEM.^161,162^ The gap between the two metal foils at that time was 5–20 μm. In comparison, modern gas-phase TEM holders have a gap of about 4 μm.^23^ In 2006, we also saw the development of a modified environmental TEM specimen holder using thin carbon windows.^163^ Between the carbon windows, a flowing and variable gas atmosphere with a pressure of up to 4 mbar could be built up. Lattice resolution was possible in this system, which allowed the investigation of morphological changes of, for example, Au NPs (e.g., while tracking the 111 lattice planes of Au) as a function of the gas phase.^164^ The same authors subsequently reported that they were able to detect conversion in CO oxidation using a Au/TiO_2_ catalyst system by attaching a mass spectrometer (MS) to the outlet of the TEM holder. The conversion was 1%, and the calculated turn over frequency (TOF) was comparable to complementary measurements inside a conventional flow reactor.^164^

**Figure 16:**
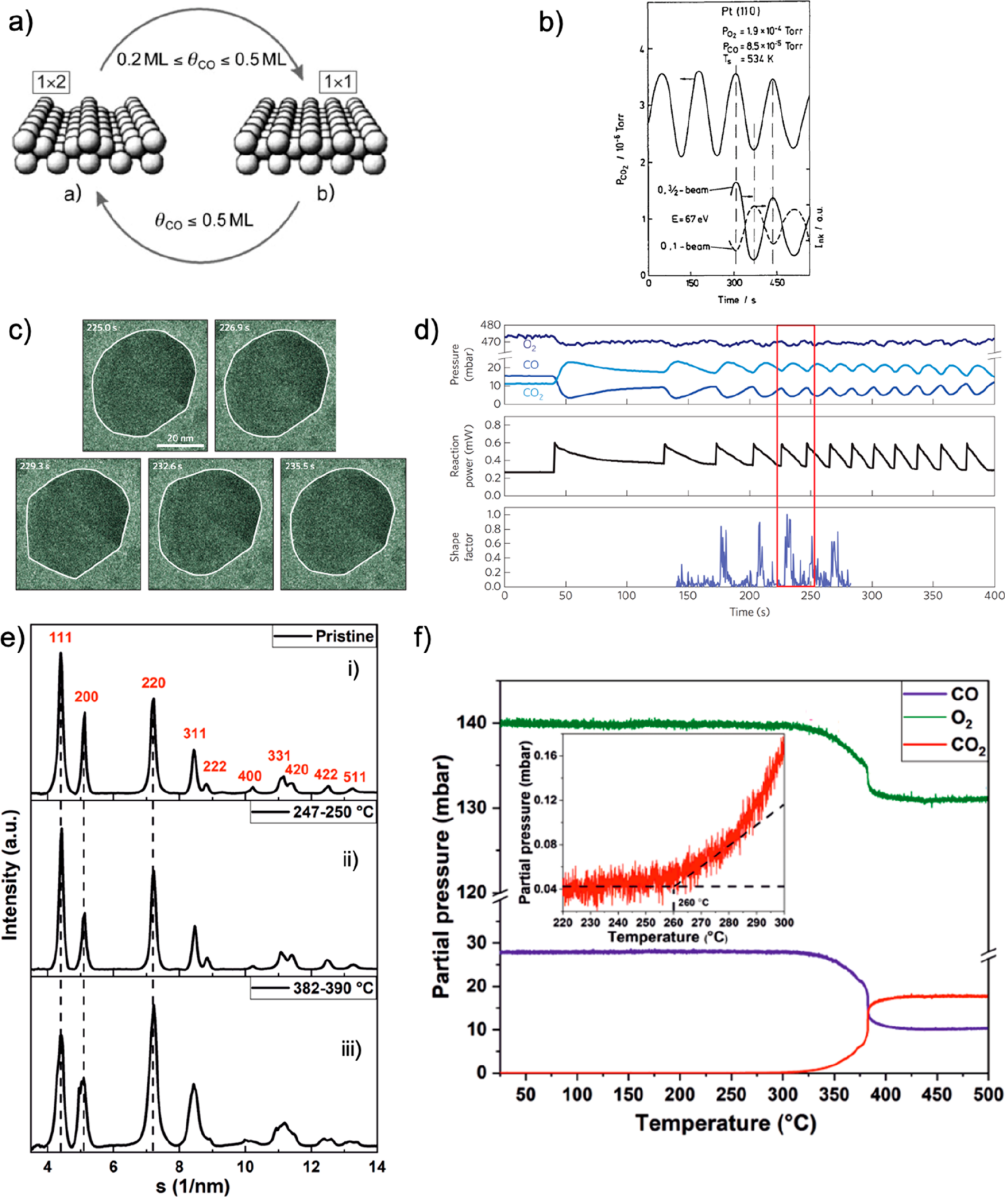
Behavior of Pt during CO oxidation–interplay of morphology and structure. (a) Schematics showing the surface reconstruction that occurred during CO oxidation over Pt single-crystal catalysts and related catalytic measurements in (b). (a) Reproduced with permission from ref (122). Copyright 2008 Wiley. (b) Reproduced with permission from ref (166). Copyright 1989 Wiley. (c) Morphological changes of the Pt NPs with (d) corresponding catalytic traces and (e) bulk structural changes found after the reaction has ignited (see catalytic data in (f)). (c and d) are adapted with permission from ref (120). Copyright 2014 Springer Nature. (e and f) Reproduced from ref (53). Copyright 2020 American Chemical Society.

Nowadays, closed-cell microelectromechanical systems (MEMS) based on Si_3_N_4_ windows^29^ see more frequent use. Their operation, properties, and history of development have already been briefly described earlier in Section 3.1 and covered in several reviews and so will not be further discussed here. Instead, we devote ourselves here to their coupling with online gas analytics. The first report of catalytic *operando* TEM studies using MEMS chips was described by Vendelbo et al.,^120^ where they focused on the dynamic behavior of Pt NPs in the CO oxidation reaction. Particularly, the authors observed a periodic refaceting of the Pt NPs that was synchronized with a periodic behavior of the online MS data. The difficulty of conducting *operando* TEM experiments for thermal gas-phase reactions is reflected by the fact that it took six years for this work to be reproduced by others. This recent effort implemented a home-built gas delivery and analysis system that allowed low flows^46^ which could be fed directly into the MS chamber. They also focused on CO oxidation over Pt NPs and showed that the bulk of the catalytic particles is not innocent in the reaction.^53^ Even in the comparatively simple reaction of CO oxidation, strain is formed inside the bulk of the Pt NPs which is reversible but needed to keep the catalysts in the highly active state. This suggests the occurrence of frustrated phase transitions. Hence, the Pt NPs exhibited chemical dynamics that were represented by the partially reversible structural dynamics needed for high activity and irreversible morphological transformation that led to faceting and deactivation. Together with the work of Ertl and co-workers,^122,165^ both *operando* TEM studies extended our view on CO oxidation over Pt NPs by showing that besides surface restructuring and morphological changes the structure of the bulk is also essential for the catalytic activity (Figure 16).

Meanwhile, the holder manufacturers are also developing commercial gas supply and analysis systems. Chemical dynamics have also been investigated for the oxidation of hydrogen over Cu NPs, in which the interaction between the catalyst and the gas drives structural transformations.^148^ These transformations depend on the chemical potential. In an intermediate temperature regime, bulk copper oxide and metallic copper can coexist and constantly interconvert. Naturally, the conversion of the oxide is accompanied by water formation. At higher temperatures, structural dynamics are expressed by surface reconstructions and redox processes involving only a monolayer of oxides. This would suggest the occurrence of different reaction mechanisms. The concept has been further extended for methanol and methane oxidation.

Besides hydrogenation reactions, mainly exothermic oxidation reactions have been investigated as shown by the examples mentioned above. These include CO oxidation, hydrogen oxidation, methane oxidation, or methanol oxidation. One of the first catalytic reactions to be investigated using modern *operando* TEM analytics and commercial gas delivery and analysis systems was the Fischer–Tropsch reaction.^167,168^ Under constant partial pressures, temperature dependencies of cobalt NPs could be reproduced, ranging from standard operation to the growth of carbon nanotubes under dynamic morphological changes as well as the formation of cobalt carbide. In the more recent study, the influence of the support on the stability of the active component was also investigated. An easier reduction of the Co-containing phase was found for silica as compared to alumina. Under reaction conditions, the shape of the NPs was preserved.

Using *operando* TEM, Pd NPs were found to behave reversibly upon heating and cooling in mixed gas environments containing O_2_ and CO.^50,121^ Below 400 °C, the Pd NPs form flat facets with a low index surface termination and are inactive toward CO oxidation. Above 400 °C, the NPs become rounder, and the conversion of CO to CO_2_ increases significantly. This behavior reverses when the temperature is subsequently lowered. Pt and Rh NPs investigated in the same study did not show this reversible restructuring.^50^ Moreover, the same NPs were observed to exhibit periodic changes from a round to a flat morphology and to change their facets during the CO oxidation reaction, leading to self-sustained oscillations in the conversion of CO to CO_2_ under constant reaction conditions.^121^ In addition, structural changes of bimetallic Ni–Rh NPs consisting of a Ni core decorated with smaller Rh NPs were followed during CO oxidation.^169^ At high oxygen partial pressure, the Ni core is partially oxidized to NiO, forming a hollow (Ni + NiO)-Rh catalyst that is highly active. Under O_2_-lean conditions, NiRh surface alloys have been formed which decrease the catalytic activity. Furthermore, it was observed that Pt–Ni alloys tend to segregate during CO oxidation.^170^

An interesting approach of a homemade *in situ* holder for TEM gas-phase experiments was described recently. Besides using small apertures for imaging with the electron beam, a miniature pressure gauge (1 × 1 × 0.3 mm) was also installed in this holder. This approach seems promising to measure the pressure directly in the sample environment in MEMS-based TEM gas holder systems.^171^

#### 3.3.3 Environmental Scanning Electron Microscopy

**Figure 17:**
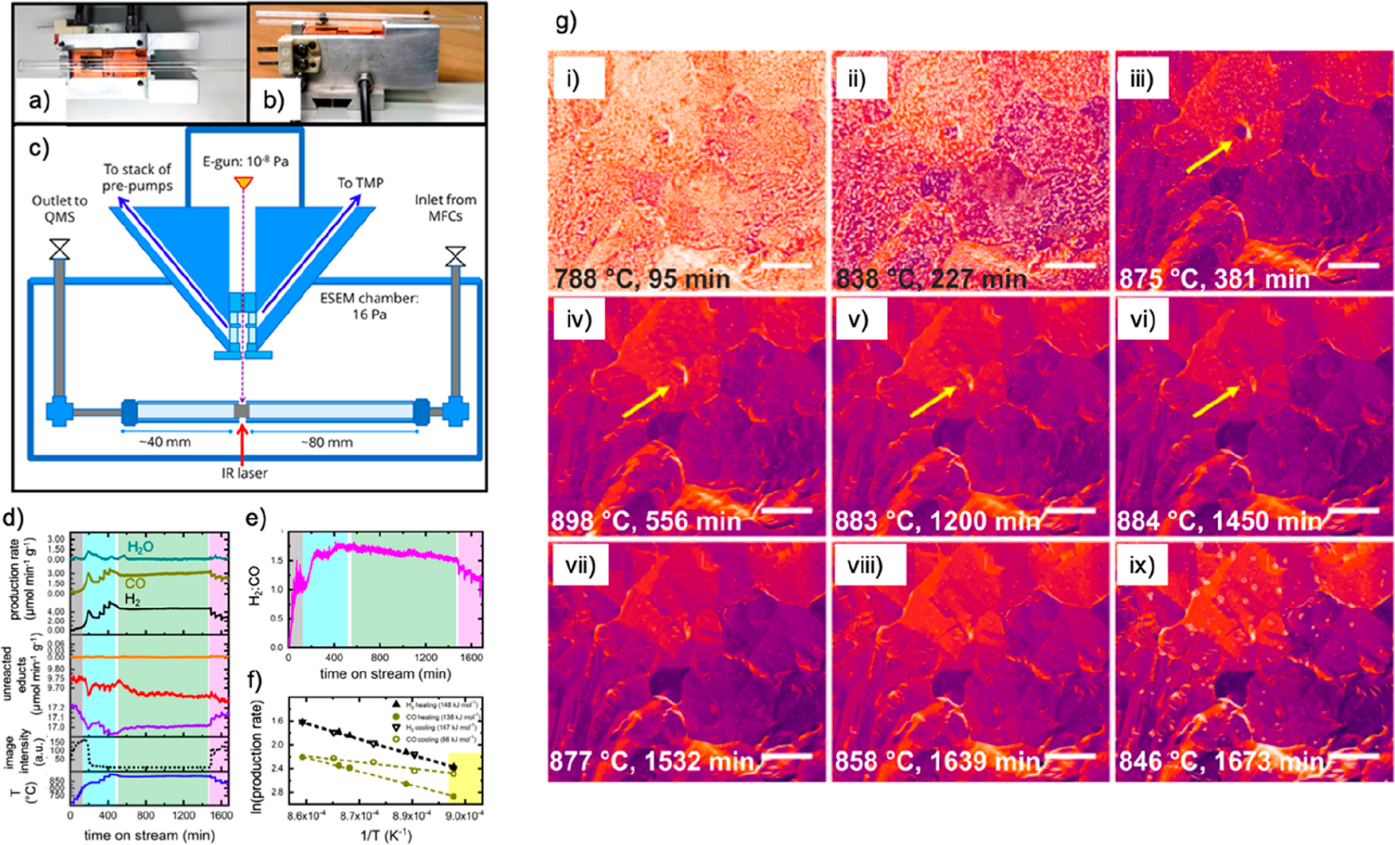
Catalytic reactions observed in an environmental SEM–DRM on Ni foam as a case study. (a)–(c) Reactor setup as incorporated into the chamber of an ESEM. (a) and (b) Photographs of a quartz tube reactor placed on the heating stage. (c) Schematics of the connection of the quartz tube reactor to the gas inlet and outlet, including MS. (d) Correlation of image intensities with catalytic data. (e) Change of the H_2_:CO ratio with time on stream and (f) Arrhenius plots for CO and H_2_ formation upon heating and cooling, showing differences in the CO production and implying different reaction mechanisms. Reproduced with permission from ref (54). Copyright 2020 Elsevier.

The use of an ESEM is promising in research related to heterogeneous catalysis because it allows for the observation of multiscale phenomena and thus gives an integral description of the changes of the catalysts as a function of the gradient of the chemical potential along the interface. Thus, there is no need to extrapolate the observed chemical dynamics from a few catalyst particles to the entire ensemble. In particular, after inserting a reactor tube into the chamber, gas flow conditions can be mimicked similar to a flow reactor^54^ (Figure 17a–c). Using the example of dry reforming of methane, it was shown that the phase transition between oxide and metal initiates the reaction and that reversible and irreversible surface changes as part of chemical dynamics can be extracted (Figure 17d–f).^54^ Furthermore, a hysteresis between heating and cooling the catalyst in the online MS data and images was observed. The associated hysteresis during heating and cooling is also reflected in the changes of the apparent activation energies for CO, while the one for H_2_ prevailed. It shows that the formation of CO and H_2_ occurs at different active sites. The hysteresis and changes of the surface states during the prevailing time of the catalytic reaction under isothermal conditions is also corroborated by the different shapes of the surface oxides after cooling. The massive morphological changes that occur during dry reforming of methane on Ni surfaces are highlighted in Figure 17g. They are related to surface oxide consumption (Figure 17g, i–iii) upon heating, transformations of the metallic surfaces (Figure 17g, iii–vi, yellow arrow), and the reappearance of differently shaped oxide structures upon cooling (Figure 17g, vii–ix).

### 3.4 *Operando* Studies of Electrocatalysts with Liquid-Phase Electron Microscopy

Liquid-phase EM occupies a unique niche in the study of electrocatalysts with its combination of high spatial and temporal resolution compared to other microscopic techniques. Optical microscopies, for example, can achieve extremely high temporal resolution, but they are generally limited in spatial resolution. Conversely, scanning probe microscopies have exceptional resolution in liquids but are limited in terms of scan speed and can be challenging to deploy with corrugated samples, such as particles. *Operando* spectroscopy methods such as Raman spectroscopy and XAS, on the other hand, can probe in detail the average properties of a many-particle ensemble, but the presence of minority or spectator species is not easy to determine from such measurements. Therefore, EC–EM complements synergistically *operando* spectroscopy studies with the visualization of different catalyst motifs that are present during reaction. It is also important to emphasize again that the restructuring of electrocatalysts in liquid electrolytes and at near ambient temperatures can result in structural motifs that are not favored thermodynamically due to kinetic limitations and involve multiple concurrent processes, such as dissolution and redeposition,^172^ which thereby lead to complex evolutionary pathways.^51^

First, we mention that in general liquid-phase EM does not attain the atomic resolution possible with TEM due to electron scattering in liquid and the need to limit beam-induced artifacts.^173^ It should be clarified here that even though there are examples of liquid-phase EM work that show surprising high-resolution images of NPs using MEMS systems, a closer inspection of such data will reveal that the images were acquired while measuring through gas bubbles or dewetted TEM chips. On this issue of spatial resolution, we also find that we tend to encounter a somewhat prevalent misconception that the technique is limited currently in its utility due to these spatial resolution constraints, and the sole prerogative in the field should be to keep pushing toward atomic resolution imaging of catalytic interfaces. Contrary to this opinion, we believe that significant opportunities exist within the current capabilities to gain insight into the multiscale dynamics of catalysts, and the possibilities for studies employing wide field-of-view, low-magnification imaging, or electron diffraction at much lower electron dose demands should not be neglected. In particular, such multiparticle data sets can complement the ensemble-averaged information obtained from *operando* spectroscopy, thereby providing a more complete picture of the catalyst behavior.^174^ Second, we reiterate here the caveat that it is debatable, namely, whether much of the current literature in the field can be considered, strictly speaking, *operando* because they only focused on the structural evolution of the electrocatalysts as a function of applied potential. The main contention is if the quality of the electrochemical measurements is sufficient for the observed changes to be correlated with their impact on the catalytic activity/selectivity of the electrocatalysts. In the cases where catalytic activity/selectivity changes were reported, it is usual that those metrics were determined using parallel benchtop setups. With this point in mind, we only discuss work that reports either parallel product analysis or concurrent product detection in detail in this section.

#### 3.4.1 Electrochemical Cell Transmission Electron Microscopy

As mentioned earlier, while there had been various attempts to incorporate a liquid environment into the TEM, the holder solutions using MEMS cells are ubiquitous for liquid-phase studies right now, and so we focus our discussion on these systems. To enable electrochemical experiments, the MEMS cells are lithographically patterned with electrodes and connected to a potentiostat outside the TEM via wiring that is integrated within the sample holder. In terms of time scales, the scan rates used in common electrochemistry protocols, such as potentiometry or amperometry, are largely compatible with the temporal resolution of EC–EM measurements. Due to the small currents associated with these microelectrodes, choosing the right potentiostat is critical to reliable measurements. The potentiostat needs to be able to measure low currents^175^ and be configurable with a floating ground.^175^ The latter is required to establish a common ground between the holder, microscope, and potentiostat and avoid electronic issues such as ground loops. These technical considerations are discussed in detail in ref (175). Similarly, electrolyte flow is achieved via fluid tubing integrated within the holder and is typically connected to a syringe pump^176^ or a pressure-based pump,^177^ and the flow geometry usually consists of one inlet and one outlet. The liquid layer thicknesses are usually controlled by the addition of spacers (typically between 50 and 500 nm) between the chips, but it should be noted that the actual thickness will be more than the spacer thickness due to membrane bulging.^178^ This results in electrolyte layers with thickness that can be as much as a couple of micrometers, which leads to a typical degraded spatial resolution of a few nanometers.^173,179,180^

**Figure 18:**
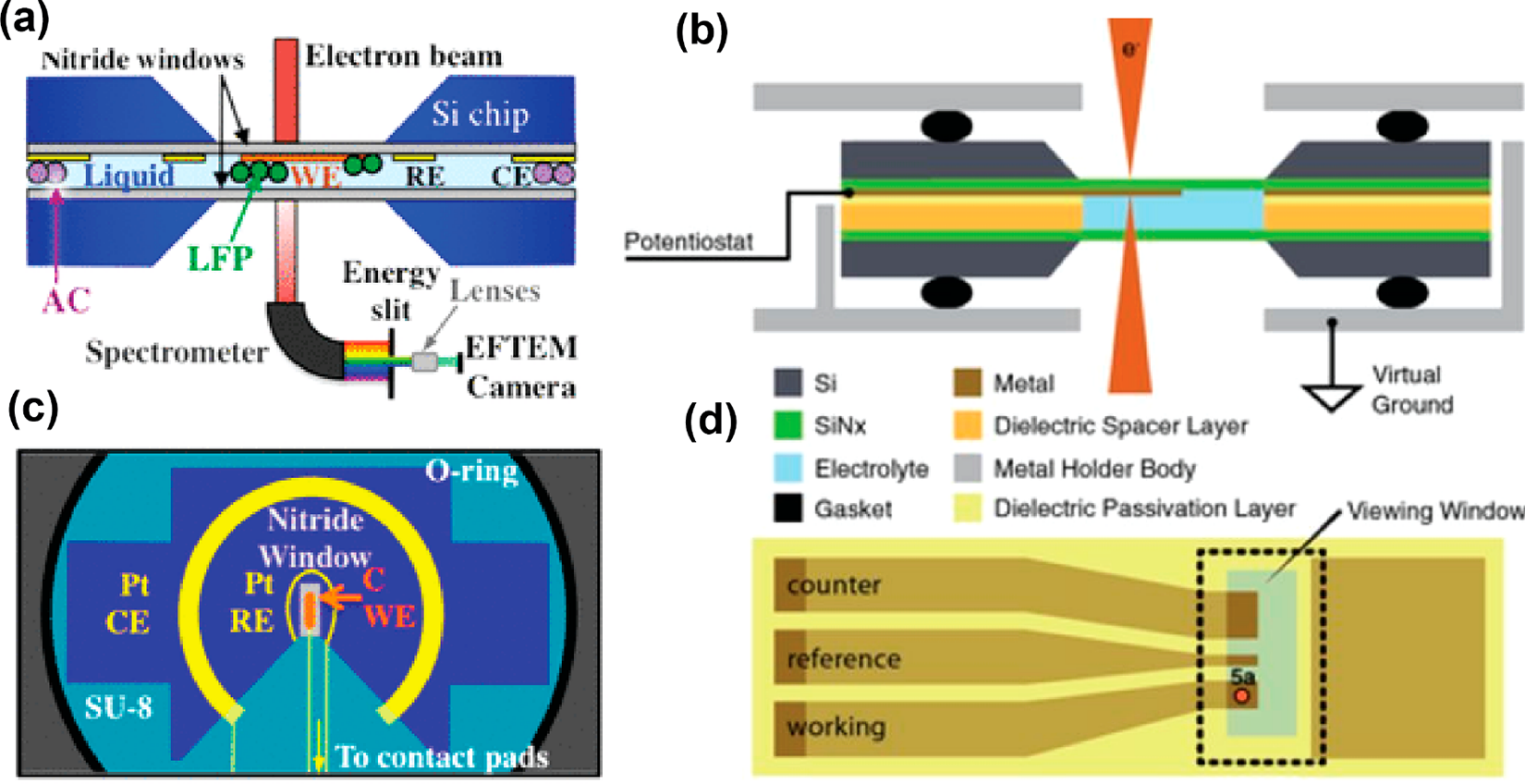
Examples of commercial electrochemistry chips. Side-view cross-section (a,b) and top-view (c,d) schematics of two electrochemical cell TEM chips with three microfabricated electrodes currently on the market. Reproduced from ref (133). Copyright 2014 American Chemical Society. Reproduced from ref (175). Copyright 2017 IOP Publishing. CC BY.

Nowadays, most EC-TEM studies are performed using electrochemical cells with a three-electrode configuration consisting of a working electrode, a reference electrode, and a counter electrode imprinted on the chips^134^ (Figure 18). Common electrode materials are Au, Pt, or C. For electrocatalysis studies, a C working electrode is preferred for comparison to catalysts used in real-world reactors that are usually dispersed on carbon supports. The reference potential in these cells is also commonly determined using a pseudo-Pt reference in the form of a Pt thin film strip on the chip.^133,181^ The pseudo-Pt reference, however, can be a source of uncertainty because unlike standard reference electrodes it is exposed to the electrolyte environment. In a regular reference electrode such as the standard hydrogen electrode, the reference is isolated from the reaction half-cell and bubbled with hydrogen gas to maintain the partial pressure of H_2_. Therefore, a drift from the reference potential in a pseudo-Pt reference electrode can occur over time^182^ due to changes in the partial pressure of H_2_^183^ in the cell or in the local reaction environment^184,185^ (such as pH of the electrolyte) that arise from the reactions on the working or counter electrode. This means that these electrodes need to be precalibrated under the same reaction conditions (applied potential, electrolyte, etc.) against a standard electrode prior to *in situ* experiments to obtain a reliable reference. We refer the reader to ref (184) for a detailed discussion on the validation of pseudoreferences with a caution that even with such validations some discrepancy may still arise from the confined geometry of these MEMS cells as compared to that found in benchtop setups.

An additional point with regard to cell design is that the working and reference electrodes should not be too close to the counter electrode. The exposed nature of these electrodes in the cell means that they can be susceptible to reactive species generated on their neighboring electrodes. In particular, the counter electrode with its higher applied overpotential can be a source of reductive or oxidative species that may interact with the samples in the imaged area. In gas-evolving reactions, bubble formation on the working and counter electrodes can also cause electrolyte dewetting in these cells and limit the overpotentials that can be applied.

To mitigate these issues, electrochemical cell holders with miniaturized Ag/AgCl references and Pt counter electrodes integrated within the holder bodies for both SEM^142^ and TEM^51^ had recently appeared on the market. With these holders we avoid the use of a pseudoreference electrode and limit the undesirable influences of the counter electrode. Note that the electrodes need to be arranged in the following order: 1. Reference, 2. Working, and then 3. Counter along the electrolyte flow path to ensure that the species generated at the customized counter electrode are pushed out of the holder and away from the working electrode. This setup was first utilized to study model Cu-based catalysts for CO_2_RR^51,186^ as we discuss below.

#### 3.4.2 Research Applications of Electrochemical Cell Electron Microscopy

In general, EC-TEM can be applied to a reaction as long as the catalyst and the electrolyte are stable under the electron beam. Reactions involving corrosive or reactive electrolytes should, nonetheless, be approached with utmost care where one needs to consider the susceptibility of all the components in the fluid path and the chance of introducing impurity species from the holder. The most common topic of study with EC-TEM is the structural changes that take place in electrocatalysts due to redox transitions during the application of an external potential. These transformations can easily elude conventional microscopy due to the restructuring or degradation that can occur when the applied potential is removed and when the catalysts are removed from the electrolyte. Another important aspect that EC-TEM studies can reveal is the aggregation or dissolution kinetics of electrocatalysts under extended operation. Examples of both applications have been exemplified in the recent work involving catalysts for electrochemical CO_2_RR.

Electrochemical CO_2_RR is a potential way to recycle CO_2_ back into valuable chemicals and fuels needed by our modern society and industries. Here, the focus has been largely focused on one electrocatalyst material, Cu, because it is the only metal that can convert CO_2_ into valuable products like ethylene and alcohols due to its optimal adsorption energies for both CO and H_2_.^187^ Despite extensive research, the selectivity of Cu-based electrocatalysts toward these desired products has remained poor. Understanding the parameters controlling catalytic activity and selectivity is difficult because of the multiple reaction pathways and reaction products in CO_2_RR^188^ and because the catalytic properties in Cu-based catalysts are highly sensitive to the catalyst surface structure and treatment.^189^ Generally, the catalysts have been found to restructure under applied potential,^172^ where the restructuring behavior can further be affected by the nature of the electrolyte.^190^ Therefore, insights into the evolution or even degradation of catalyst structures under reaction conditions^172,189^ are key for enabling the rational design of optimal catalysts, and so the CO_2_RR system has become a relatively popular system for EC-TEM studies.^51,186,191−194^

**Figure 19:**
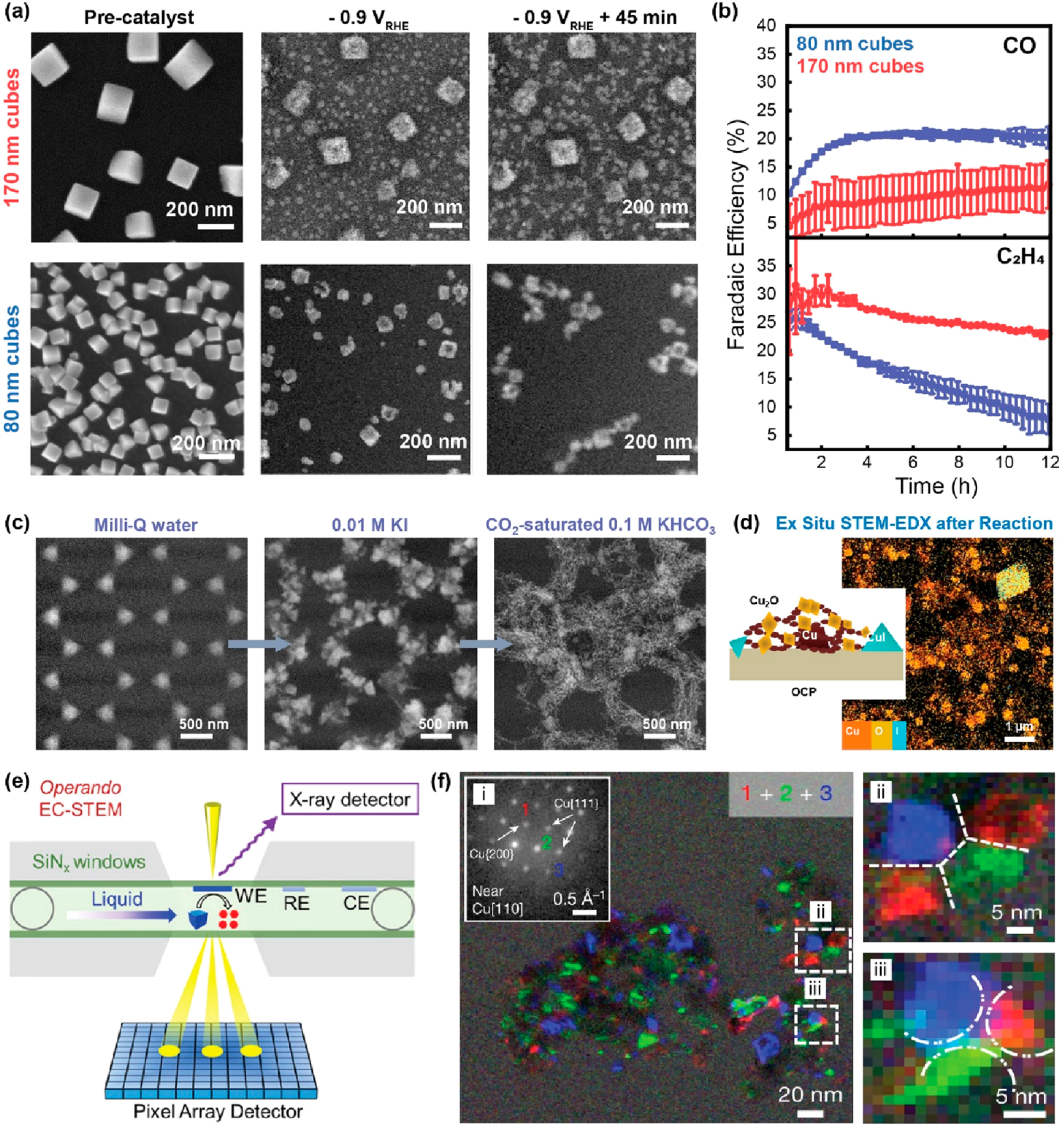
Restructuring of Cu-based electrocatalysts during CO_2_RR. (a) EC-STEM image sequence showing the evolution of two sets of Cu_2_O cubes synthesized with different size and loading in CO_2_-saturated 0.1 M KHCO_3_. The larger 170 nm cubes exhibit predominantly fragmentation and redeposition under sustained applied potential of −0.9 V_RHE_, whereas smaller 80 nm cubes undergo severe catalyst detachment in the electrolyte together with catalyst aggregation during reaction. (b) A comparison of the Faradaic efficiency of similarly synthesized sample sets toward CO and C_2_H_4_ measured using only gas chromatography at −1.1 V_RHE_. Adapted in part from ref (51). Copyright 2021 Springer Nature. CC BY 4.0. (c) EC-STEM image sequence showing the evolution of lithographically patterned Cu islands following anodization in 0.01 M KI and then in iodide-free CO_2_-saturated 0.1 M KHCO_3_ at −1.0 V_RHE_. (d) Post-mortem STEM-EDX map taken after reaction, indicating the reprecipitation of Cu_2_O and CuI after returning to the open-circuit potential. The inset depicts a schematic of the after-reaction morphology. Adapted in part from ref (186). Copyright 2022 Royal Society of Chemistry. CC BY 3.0. (e) Schematic describing the application of 4D-STEM to EC experiments. Adapted from ref (195). Copyright 2022 American Chemical Society. (f) False-colored dark-field 4D STEM maps depicting Cu nanograins with individual diffraction patterns that can be matched to the diffraction spots indicated in inset (i), where 1 (red) corresponds to metallic Cu {200} (with 1.8 Å) and 2 (green) and 3 (blue) correspond to different Cu {111} (2.1 Å) spots that are close to the [110] zone axis. (ii) and (iii) show enlarged images as indicated by the dashed boxes and indicate (ii) loosely connected Cu nanograins and (iii) overlapping nanograin boundaries, respectively. Reproduced in part from ref (194). Copyright 2023 Springer Nature.

Here, linear sweep^51,186^ or cyclic voltammetry^51,186,194^ can be used to ensure that the potentials applied to the catalysts during the EC-TEM experiments are the ones required to achieve CO_2_RR. Linking the EC-TEM results with their corresponding impact on catalytic selectivity, while crucial for obtaining more meaningful insights from these studies, is not straightforward as we will elaborate later. We first discuss two studies that reported selectivity data, albeit from benchtop online gas chromatography and time-resolved liquid gas chromatography measurements of similar catalysts. In the first work,^51^ electrodeposition was used to synthesize reproducibly well-controlled model catalysts on both the EC-TEM chip and the bulk glassy carbon supports used in benchtop experiments. *Ex situ* SEM was used to verify that Cu_2_O cubes of similar sizes and loading were electrodeposited, and then, the evolution of catalyst size and loading as extracted from the movies recorded during EC-TEM experiments were compared with the time-resolved trends of the different gaseous products measured with gas chromatography. The *in situ* recorded movies revealed that the cubes undergo a range of restructuring processes, which include catalyst detachment, redeposition, fragmentation, and aggregation, during the initial ramp toward cathodic potentials of CO_2_RR and during sustained applied potential (as shown in Figure 19(a)), thereby increasing the variety of structural motifs present during the reaction. Moreover, the motifs evolved over time, where isolated Cu NPs gradually aggregated into short nanoparticle chains. A comparison of the resultant combined catalyst loading (both motifs) obtained from the movies (Figure 19(a)) against the time-resolved CO and C_2_H_2_ production over extended reaction times obtained from parallel measurements (Figure 19(b)) suggests that the hydrocarbon selectivity was correlated with the ensemble loading of both catalyst types, where the 170 nm sample with more redeposition and less catalyst detachment sustained its ethylene selectivity. More importantly, this study established that despite restructuring the starting catalyst characteristics still determine the properties of the electrocatalyst ensemble with each sample set having a distinct selectivity character toward hydrocarbon formation.

In the second work, the same group used a different synthesis approach to understand the impact of iodide species on the catalytic selectivity of Cu.^186^ Here, inverse sphere lithography and physical vapor deposition were used to deposit hexagonal arrays of metallic Cu islands. Product analysis from these islands confirmed that the selectivity improvements from iodide treatments reported in bulk Cu foils also transferred to these samples. Then, the islands were tracked sequentially using EC-TEM through the treatment with KI and then CO_2_RR, which showed first the formation of CuI pyramids followed by their transformation into fragmented filaments in CO_2_-saturated 0.1 M KHCO_3_ and under applied potential (Figure 19(c)). Interestingly, continued *in situ* observations after removing the applied potential and going back to open-circuit potential showed that particulate precipitates reappeared under open-circuit conditions, which were later confirmed to be a mix of Cu_2_O and CuI particles with *ex situ* TEM (Figure 19(d)). These results imply that Cu^+^ and I^–^ species rather than Cu_2_O/CuI particles are present under cathodic reaction conditions, where Cu^+^ is commonly associated with hydrocarbon selectivity during CO_2_RR.

Recently, Yang et al. reported the use of 4-dimensional STEM (4D-STEM, 2 dimensions in real space, 2 dimensions in diffraction space) to track the reorganization of Cu NPs of different sizes (7–18 nm) during CO_2_RR (Figure 19(e),(f)).^194^ The authors were able to show that the NPs reorganized in multidomain granular structures made up of metallic Cu nanograins under reaction, albeit after a gas bubble was created by the electrolysis. More importantly, these measurements were complemented with resonant soft X-ray absorption spectroscopy data to identify the chemical state of the working catalysts and offline differential electrochemical mass spectrometry^196^ (DEMS) measurements to track the evolved products and correlate the product evolution with the structural evolution. Based on these measurements, the authors concluded that the nanograins were active toward CO_2_RR. The potential of imaging strategies based on computational methods demonstrated by this work may pave the way for the future application of scanning-diffraction methods to attain higher spatial resolution in liquid-phase experiments.

Another popular reaction studied via EC-TEM studies is OER.^197−200^ OER is a simpler reaction that does not have a selectivity distribution since O_2_ is the only reaction product, and four electrons are transferred. It is also generally accepted to be the limiting half-cell reaction in water splitting due to the sluggish kinetics of the multiple electron transfer processes. Using water splitting to generate green hydrogen as a fuel is, nonetheless, a key part of our efforts to move away from a fossil-fuel-based economy, and thus intense research activity exists focusing on developing optimal catalysts for OER. In the related application of photocatalysis, there has been progress made using liquid cell holders to study water-splitting catalysts where the electron beam is used as a substitute energy source^201^ or with an integrated light source,^202^ where the presence of H_2_ has been shown by EELS measurements in an evolved gas bubble.

**Figure 20:**
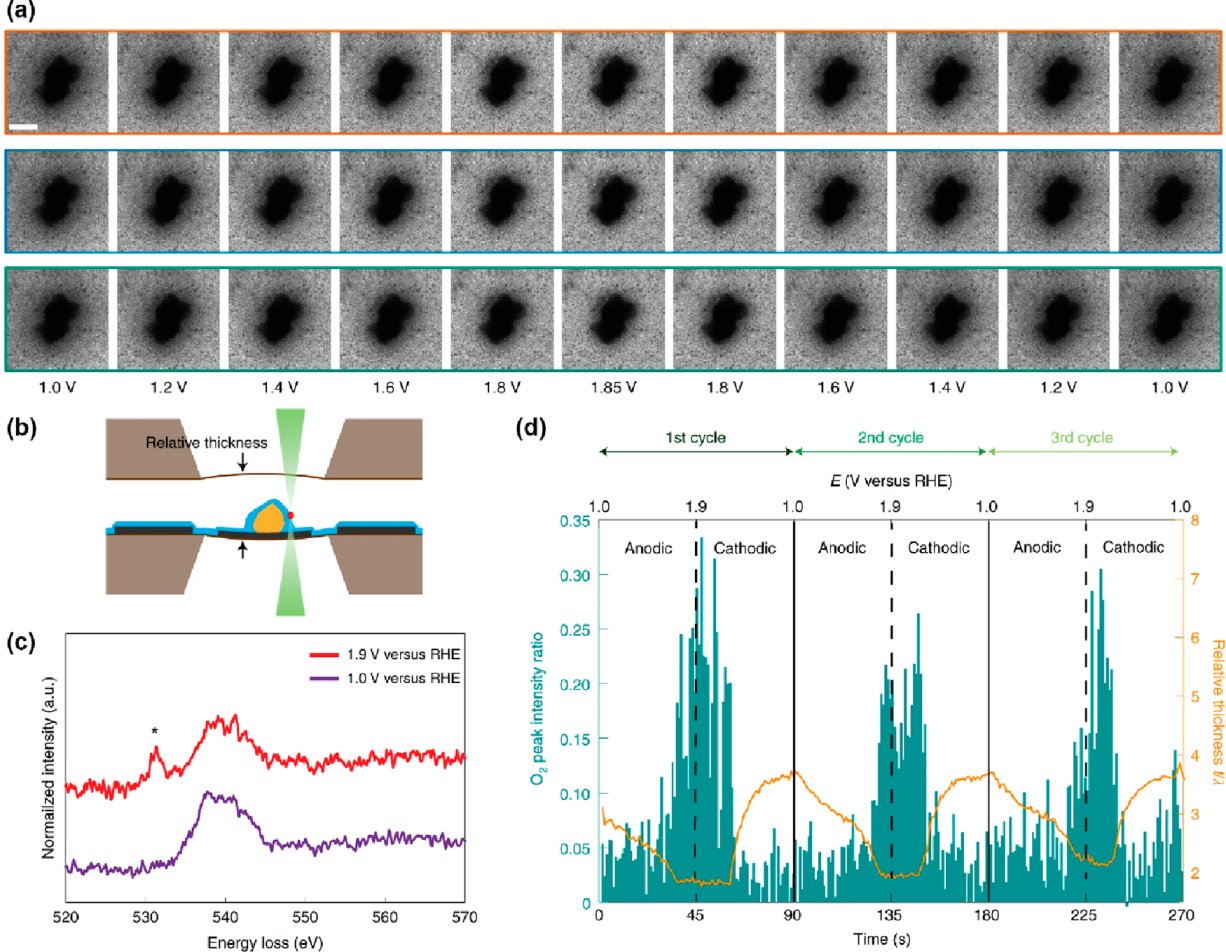
Real-time imaging of a BSCF particle and concurrent EELS measurement of oxygen evolution during cyclic voltammetry. (a) Bright-field TEM images at different potential stages for the first, second, and third cycles. Scale bar is 400 nm. (b) Schematic of STEM-EELS probing near BSCF particles in the EC-TEM cell. (c) Oxygen K EEL spectra acquired at 1.0 and 1.9 V_RHE_. The asterisk at 531 eV indicates the peak feature from molecular oxygen at higher potential. (d) Plot of O_2_ peak intensity ratio (green) and relative thickness (orange curve) as a function of elapsed time (bottom axis) and applied potential (top axis) corresponding to CV measurements. Adapted in part from ref (200). Copyright 2021 Springer Nature. CC BY.

For OER, we highlight recent work using EC-TEM to investigate the behavior of the complex perovskite catalysts that we mentioned earlier where EELS was also used to track O_2_ evolution under different applied potential regimes.^200^ It was found that BSCF catalyst particles exhibited potential-dependent fluctuations in the bright-field TEM image contrast (Figure 20), which indicated the movement of the surrounding alkaline solution. Note that in these experiments the catalyst particles were not fully immersed in liquid (i.e., the cell was not fully filled), and so the contrast variations could be associated with changes in the wettability of the catalysts in response to switching the surface hydrophobicity/hydrophilicity at different potential regimes. Specifically, at low applied potential (1.0 V_RHE_ < V < 1.2 V_RHE_), the particles exhibited reducing hydrophobicity due to electrowetting, which also changed the interfacial capacitance. At 1.2 V_RHE_, the formation of a surface oxyhydroxide phase then led to hydrophilic wetting at intermediate potentials. This hydrophilic phase was attributed to the Co^2+^/Co^3+^ redox reaction. For potentials higher than 1.65 V_RHE_, the surface oxyhydroxide further catalyzed the conversion of adsorbed hydroxide ions into O_2_ at the solid–liquid interface, which was verified by correlated changes in the O peak intensity in the EELS spectra and further thinned the liquid layer. Even though the work was performed under the restrictive conditions of a thin liquid layer, it demonstrates the potential of using EELS to probe product formation under electrochemical conditions.

Catalysts for proton exchange membrane fuel cells (PEMFCs)^183,203−205^ have also been studied with EC-TEM, and so they will be briefly discussed here with an eye on *operando* work in the future. Fuel cells are the conceptual opposite of the catalytic processes we have described so far where gas or liquid fuels, such as hydrogen, methane, propane, or gasoline, are recombined with an oxidant, typically oxygen, to transform chemical energy back into electrical energy. PEMFCs consist of a proton-conducting polymer (ionomer) membrane sandwiched between the catalysts and the electrodes (cathode and anode). Like OER in water splitting, oxygen reduction (ORR) is the rate-limiting reaction that determines the conversion efficiency of these fuel cells. Currently, the catalysts for ORR still mainly consist of expensive Pt-based (Pt or Pt-transition metal alloys) NPs that are supported on a carbon support. Some of the key issues affecting the long-term stability of PEMFCs are catalyst dissolution and support degradation. In this aspect, TEM, especially identical location TEM, where the same sample was followed at different reaction durations, had been crucial in identifying the dominant catalyst degradation mechanisms^206,207^ in fuel cells.

It should, however, be noted that the microfluidic cell geometry used in EC-TEM studies differs significantly from the working geometry of currently deployed commercial fuel cells. In addition, the reported experiments had been performed under simulated reaction conditions where the catalysts reacted in aqueous perchloric acid^208^ and mostly in the absence of any ionomer (with the exception of ref (205)). We also mention here that whether the conclusions drawn from studies performed in aqueous electrolytes can really be extended to real fuel cell behavior remains an ongoing general debate in the fuel cell community.^209,210^ Broadly, these differences make EC-TEM results harder to extrapolate toward real systems, but cells that more accurately replicate the geometry of a working fuel cell should be conceivable for the ESEM and will enable probing of ORR catalysts under operating conditions.

#### 3.4.3 Function Determination in Liquid-Phase Experiments

As mentioned earlier, we can in principle use the cyclic voltammetry, chronovoltammetry, or chronoamperometry etc. to determine changes in the ensemble properties of the electrocatalysts. With electrochemical measurements, it is generally simpler to interpret transformations that are irreversible at fixed potential, such as the oxidation and reduction of the electrocatalysts, whereas relating the changes in currents (activity) measured during cyclic voltammetry or chronoamperometry experiments to specific structural features is less straightforward. The main reason is because the electrochemical response of individual catalysts is not distinguishable from the overall signal. Detailed information regarding the catalyst loading on the entire working electrode, the size of the working electrode, total catalyst active surface area, and how those parameters evolved over the course of the reaction is hence required for quantitative or qualitative correlation of the structural changes to the changes in measured signals, which also places a high demand for the stability of the measurement setup. Getting reliable electrochemical data can be challenging especially when the small amounts of sample that can be deposited on the EC-TEM chips also means that we usually have low currents.

We further mention here that some electrochemical characterization methods used routinely on bulk single crystals and foils do not translate easily to EC-TEM experiments. For example, the electrochemical surface area is an important parameter for benchmarking the activity of a catalyst, which can be derived using methods such as measurements of the double-layer capacitance or underpotential deposition. However, as we have alluded to in ref (51), we should not assume that these supposedly benign measurements that involve applying potential sweeps for instance during electrochemical double-layer measurements or adsorbing/depositing of gaseous/atomic species do not alter the morphology of the electrocatalysts. For example, it was reported that the CO_2_RR selectivity of Cu electrocatalysts can shift toward methane production after electrochemical double-layer capacitance measurements.^211^

Unlike gas-phase experiments, there has been no demonstration of online reaction product detection for EC–EM experiments so far. Real-time product analysis in EC–EM experiments is, unfortunately, not easily achievable in our opinion. Product detection and quantification with standard laboratory-scale methods, such as gas and liquid chromatography, usually require product accumulation times in the tens of minutes and slow response rates, which are much longer than the time scales we are probing with EM experiments. This makes it difficult to correlate the observed structural changes with their impact on catalytic selectivity if those changes occur on shorter time scales. The relatively scant number of catalysts in an EC–EM setup is also not conducive for measuring products even if they are accumulated over longer durations, which highlights a need for fast but highly sensitive methods.

The case for an effort to enable rapid product analysis that matches structural characterization lies in the need to rationalize the chemical dynamics and catalyst evolution that occur under the start–stop conditions found in conversion technologies relying on intermittent renewable energy and understand how they determine the ultimate performance of the catalysts during long-term operation.^46^ Among the various techniques available, DEMS, especially systems based on microfluidic electrochemical cells,^212^ have potentially the time resolution required to keep up with the changes in the reaction productions. Its integration into EC-TEM systems is, however, not without its own challenges. Conceptually, one can think about attaching a fluid line to a mass spectrometer modified to perform DEMS^196^ in an approach similar to the gas systems, but this idea falls apart upon closer scrutiny. On the one hand, within these holders, there are reactions taking place on both the working and counter electrodes, and their products are not differentiated in the electrolyte stream, which contrasts with the case of gas-phase experiments where the catalysts inside the reaction cell are the only source of products. In all setups for electrocatalytic product analysis including DEMS, the counter and working electrodes are separated into individual compartments precisely to prevent interference due to products generated at the counter electrode. Such a separation is, however, difficult to implement in existing EC-TEM solutions due to the space constraints within TEM. Moreover, there is a relatively long fluid path from the working electrode to the outside of the holder, and the chemical composition of the electrolyte can change when the product-carrying electrolyte passes by a neighboring electrode. This mixing of the reaction products coupled with the small number of product-producing catalysts available on the micrometer-sized working electrodes and the product dilution due to the long fluidic path makes any quantitative detection of the product distribution changes very challenging. Here, a path forward might reside in a major modification of the cell and holder design to make it compatible with DEMS measurements, although that is not straightforward due to the above-mentioned space restrictions.

### 3.5 The Ubiquitous Electron Beam: Identification and Mitigation of Beam-Induced Artifacts

**Figure 21:**
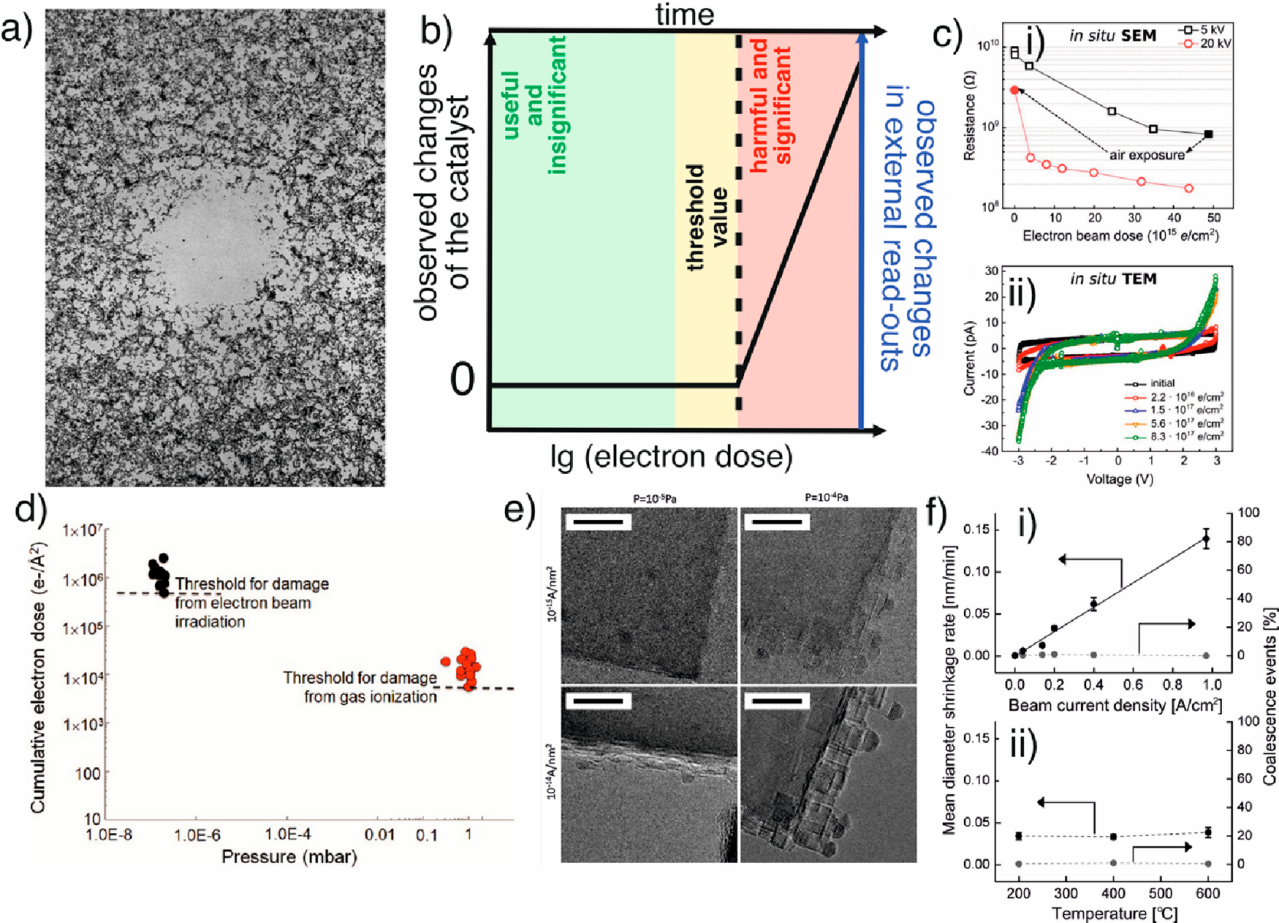
Manifestations of beam damage during *in situ* TEM experiments in the gas phase. (a) The image shows soot particles. The empty spot in the center (diameter: approximately 5 μm) was exposed to 0.2 A cm^–2^ for 1 min at 800 mbar Ar. Image scale 2600:1. Reproduced with permission from ref (161). Copyright 1969 Springer Nature. (b) Schematic for thinking about the impact of the electron beam. The regions are grouped as suggested by the review published by Rivzi et al.^221^ Green regions denote when the electron impact is insignificant on the observation, and the obtained results can be considered useful. Yellow describes conditions where there is already noticeable beam-induced artifacts but where the thermal or chemical stimuli still have a stronger effect on the catalyst behavior. Under such conditions, the results can be useful only under conditions where the beam effect is quantifiable. Red areas indicate significant electron beam interaction which are harmful to the catalysts. The presented charts show experimental observables, such as morphological (perimeter effects, particle shape, dynamic behavior, etc.) and structural changes (uncovered by electron diffraction, left, *y*-axis), that can be influenced significantly if the electron beam is not controlled precisely or over time. Furthermore, the ion currents of the MS, heating power of the MEMS chip, or current changes can be affected by the electron beam and, thus, require thorough inspection before the true *operando* experiment can be conducted. This is reflected by the right *y*-axis labeled “observed changes in external read-outs. (c) Variation of the resistance of barium titanate as a function of the electron dose and acceleration voltages (i) implying the formation of oxygen vacancies. (ii) Dose-dependent transition of barium titanate from insulating to semiconducting. Reproduced with permission from ref (214). Copyright 2020 Wiley. (d) Threshold electron doses to damage multiwall carbon nanotubes in vacuum and in gas environments at room temperature showing that gas ionization is more severe then electron beam irradiation. Reproduced from ref (223). Copyright 2016 American Chemical Society. (e) ETEM investigation of Au/MgO cubes exposed to different electron doses and water vapor pressures. Water vapor is a common byproduct in catalytic reactions. The scale bars are 5 nm. Reproduced with permission from ref (154). Copyright 2012 Elsevier. (f) Particle shrinkage rate and coalescence of Pt/Al_2_O_3_ catalyst in 10 mbar air at 400 °C as a function of beam current density (i). For comparison, the same parameters are plotted as a function of temperature at constant beam current density (ii). Reproduced from ref (219). Copyright 2016 American Chemical Society.

The effect the electron beam has on these experiments is a commonly raised concern and so we addressed it here meticulously. As early as the 1960s, Heide has reported enormous beam damage when a sample in a gas phase comes into contact with the electron beam at 1 bar.^161^ They impressively showed that at room temperature in an argon atmosphere carbon can be removed in the illuminated area by the electron beam from the electron-transparent membranes of the cells. Carbon removal in argon is chemically impossible even at high temperatures (Figure 21a). It can only be explained by the presence of reactive radical species formed when the electron beam interacts with the gaseous atmosphere. Thus, the gas–electron interaction can lead to additional imaging artifacts that are chemical in nature: the interaction of radical species with the catalyst. Similarly, the electron beam can affect heterogeneous catalysts in their reactive environments.

It should be noted here that the electron beam can change not only the geometric and electronic structure of solids^213^ but also the performance behavior of functional solids.^214^ Hence, many strategies have been developed to reduce the effect of radiation damage on TEM observations, including low-temperature imaging.^215^

For illustration purposes, a heterogeneously catalyzed oxidation of hydrocarbons is used. The composition of the reactants usually consists of oxygen and hydrocarbons, such as propane or propylene. In addition, water can be added to increase selectivity.^118^ The product mixture contains the total combustion products CO_2_ and H_2_O, functionalized hydrocarbons, and unreacted educts which are likely to be found in the atmosphere around a catalyst particle. These components are intermediates or ingredients of most of the catalytic reactions, and their interaction with the electron beam is part of the following discussion.

Water ionizes when interacting with fast electrons, and additional excitation can lead to the formation of radicals which can form a variety of reactive intermediates over consecutive reaction mechanisms.^216,217^ The following considerations reflect the extreme case of a condensed phase. Note, the average diffusion length of radicals in the gas phase is larger as compared to liquids. The generated radicals from water can react with each other and can form six reactive species with different reduction potentials in addition to the electron beam. These species involve H*, H^–^, OH^–^, HO_2_*, H_2_O_2_, and HO*.^216,217^ From these species, complex reaction networks would allow the formation of 97 different species with varying stabilities, including H_2_, H_3_O^+^, or the Zündel cation. Chemically, some of these species exhibit a larger oxidation potential than hydrogen peroxide (e.g., the hydroxyl radical) or exhibit similar reductive capabilities as the electron beam (e.g., via the formation of hydrogen radicals). These active species can directly react with other species which would in extreme cases falsify the product distribution, interact with the catalysts, and change their chemistry, structure, and morphology or the local chemical potential and pH values around the catalyst and, thus, induce morpho-dynamical alterations. The electron-induced modification of the environment can occur on the same temporal scale as the catalytic cycle, and so it can directly influence it.^217^ Electron-induced radicals of hydrocarbons can be stabilized by intramolecular inductive or resonance effects, which would lower their reactivity, inhibit their catalytic reactions, and enhance their lifetimes.^217^ Furthermore, O_2_ is stepwise reduced to O^2–^ by the uptake of electrons. These superperoxide and peroxide intermediates, also known as electrophilic oxygen species, are generally believed to be oxidative. However, the superoxide (O_2_^–^) can be reductive and can induce structural phenomena similar to hydrogen.^29^ Moreover, since our reactions are never performed in pure water, the ions in solution can further modify the radical chemistry.^218^ Our fundamental understanding of this aspect is particularly poor.

Since all gases or liquids are prone to radical formation, it should be clear that identifying and limiting the beam-induced artifacts on the solid catalyst and our observations are key to acquiring results that are reflective of processes in real-world systems. This is an issue that can only be mitigated and not eliminated since electrons are needed to form the images. We also highlight here that in all cases of *operando* investigations using energetic beams (i.e., electrons, X-rays, lasers) beam-induced changes in the samples are an ever-present concern but with the nanoscale microscopic methods such as EM, we can visualize the effect of the electron irradiation and, in turn, rationalize the impact on the observed dynamics and structures with appropriate control experiments. This is not a trivial task and involves time, chemical preknowledge on the reactivity of the system, and the patience to conduct additional complementary and ideally correlative measurements. The usual approaches for identifying beam-induced effects include looking at areas irradiated and not irradiated by the electron beam before and after reaction, repeating the experiments in the absence of the electron imaging and comparing data collected at different dose rates. Although these steps may be seemingly straightforward, it is not difficult to find examples in existing literature where these control experiments are incorrectly implemented. For example, the electron flux used is not always reported in papers. We also mention here that while dose rate dependence studies are important for rationalizing the effects of the electron beam in terms of its dose rate dependence or determining if there exists a threshold dose, it is, on its own, insufficient for establishing the absence of beam-induced changes because the threshold for beam-induced processes is sample dependent and can be very low in some cases.^24^ Therefore, these threshold values have to be tested in a systematic and rigorous manner (Figure 21b), and this testing has to be repeated once the sample and the reaction environment are altered.

It is also important to note here that the manifestation of the artifacts induced can depend on both the employed electron flux and the accumulated electron dose.^24,214^ Commonly seen effects are presented in Figure 21c–f. In the gas phase, beam-induced artifacts include particle sintering and growth,^219^ structural alterations,^154,170,220^ or the formation of new nanostructures.^154^ Furthermore, these studies show that significant beam-induced artifacts can already set in for gas-phase experiments at electron fluxes of a couple hundred e^–^ Å^–2^ s^–1^, while we emphasize again that these threshold fluxes can change with the system. In the liquid phase, the electron beam can drive both nucleation and dissolution of solid phases^211,215^ and bubble formation due to the accumulation of radiolytic products.^211,216^ The latter was commonly used in the past to reduce the liquid layer and obtain higher-imaging resolution, but nowadays, it is more routine to mitigate the issue with low dose imaging and a sustained liquid flow. Recent work in the field of electrocatalysis is converging toward the use of electron fluxes of a few electrons e^–^ Å^–2^ s^–1^ and below for these experiments.^51,179,187,193^

In this aspect, we need also to recognize that there are different degrees to the effects induced by the electron beam since it is unlikely that we fully eliminate all influence of the electron beam even at the lowest electron fluxes. Specifically, the concept of classifying electron–sample interactions in terms of useful/harmful and significant/insignificant that was posited recently by Rivzi et al.^221^ for liquid cell molecular assembly work is similarly relevant for studies of catalyst behavior (Figure 21(b)). The salient point here is that the impact of electron–sample interactions needs to be considered within the context of a study’s goals. In the case of heterogeneous catalysis studies, it means that the imaging strategy and approach can change depending on whether the goal of the study is to identify the prevalent catalyst morphology or to track catalyst restructuring dynamics. In the former, it may be enough to only show that electron irradiation does not alter the structural characteristics of the investigated catalysts, and so, one can use intermittent imaging strategies to further reduce the electron dose on the sample. On the other hand, if our aim is to extract the catalyst restructuring behavior, continuous imaging is then unavoidable. In this case, low electron flux imaging will be necessary with additional control experiments performed to demonstrate that beam-induced effects have a weaker impact on the dynamics compared to the chemical driving forces (e.g., temperature or applied potential) and that we can qualify how the electron beam irradiation shifts the observed behavior (e.g., accelerated structural change or offsets in effective temperature/applied potential). An example of this idea was put forward by Bugnet et al.^222^ where they showed that the beam-induced reduction of ceria can be compensated by the presence of oxygen in the gas environment during *in situ* observations.

We also mention that there are ongoing efforts to limit the electron dose deposited by altering how the images are acquired, particularly in STEM mode, and reduce the dose deposited per frame. Some of these ideas include sparse sampling and more efficient scan geometries that are different from the standard raster scans, such as spiral or serpentine scans.^224−226^

Before we conclude this section, we just briefly mention here that these control experiments sometimes also reveal interesting causes behind discrepancies between *in situ* and *ex situ* observations. While the possibility of beam-induced artifacts is probably the most common comment on any novel discovery made with EM, we should bear in mind that all measurements come with an associated possibility of artifacts, including *ex situ* comparisons. For example, in ref (51), Grosse et al. demonstrated that the simple act of rinsing a reacted sample with water can be enough to lead to the detachment of loosely attached catalyst particles and, hence, catalyst loss, which results in an undercounting of the actual catalyst loading. Another important consideration here is the sample and environmental homogeneity. For example, samples deposited using drop-casting may not always be distributed reproducibily on the chips, and thermal gradients exist on the heating chips^227^ where the heater center is hotter than the outside. These gradients need to be considered when we evaluate data from different areas of the MEMS reactors or electrochemistry chips. The samples are also typically not entirely homogeneous and monodisperse even when made with the best colloidal synthesis methods. Teasing out these variations will require repeated experiments under identical conditions but is critical for ensuring our experiment reflects the real-world processes.

## 4 Imaging and Spectroscopy in *Operando* EM Studies

An understanding of the capabilities and limitations of a technique is key to using it effectively and to avoid common pitfalls and artifacts. Here, we focus on the technical aspects of *operando* EM especially on the spatiotemporal resolution in imaging and spectroscopy.

### 4.1 Spatial Resolution

**Figure 22:**
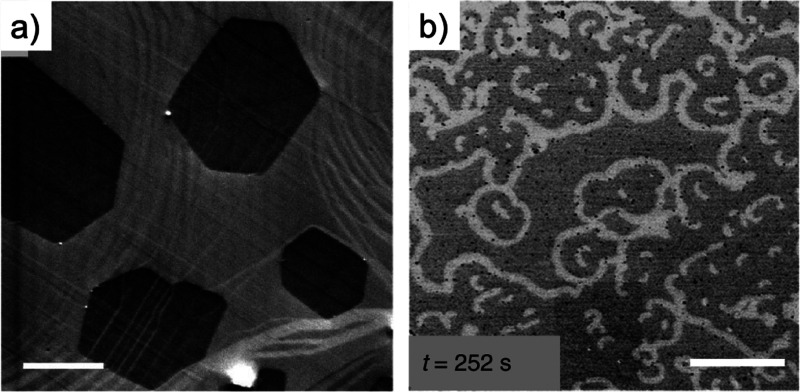
Surface sensitivity of the ESEM. (a) Observation of graphene growth on Cu and (b) spatiotemporal pattern during NO_2_ reduction at 13 Pa. (a) Reproduced from ref (232). Copyright 2015 American Chemical Society. (b) Reproduced in part with permission from ref (230). Copyright 2020 Nature Springer.

The achievable spatial resolution in the *in situ* and *operando* EM studies is determined by the approach, imaging mode, sample, and reaction environment. For gas-phase experiments, lattice resolution is generally retained except at the higher pressures or when using thicker membranes.^228,229^ In SEM, the classical SE signal detected by Everhart–Thornley detectors cannot be used at higher gas pressures (above 2 × 10^–2^ Pa).^230^ Instead, gaseous secondary electron detectors (GSEDs) have been developed that are exclusively available in dedicated ESEMs. The detector is positioned at the bottom of the pole piece directly above the sample. A positive potential of a few hundred volts is applied that accelerates electrons emitted from the samples toward the detector. On their way to the detector, the emitted electrons collide with gas molecules. The ionization process, which is repeated many times, creates environmental secondary electrons and positive ions and results in a proportional cascade amplification of the original secondary electron signal. This signal is detected by the GSED and forwarded to an electronic multiplier. Thus, the detected signal is directly affected by the gas in the chamber.^231^ The easier it is ionized, the better the amplification process. It has been shown that similar to photoemission electron microscopy (PEEM) the detected signal can be sensitive to work function changes of the surface, leading to monolayer sensitivity despite its lateral resolution limitation of a few nanometers (Figure 22).^230,232^

For liquids, imaging in TEM or STEM mode generally preserves nanometer resolution for samples that have high mass or atomic number (*Z*) contrast if the liquid layer can be kept to no more than several hundred nanometers thick.^173^ Thicker samples or liquid layers reduce the spatial resolution due to multiple scattering of the electrons as they propagate through cells, which also leads to energy loss and reduced coherence in the transmitted electrons. STEM, in particular, is better suited for imaging high *Z* elements and in thicker liquids.^173^ It is also important to note that the location of the sample (i.e., on the top or bottom membrane) matters significantly in terms of imaging performance (also referred to as the top–bottom effect^173^). With STEM, samples on the top membrane are imaged at better resolution compared to samples on the bottom because the focused electron probe broadens as it propagates through the liquid, resulting in blurred images for samples on the bottom membrane. Conversely, the samples on the bottom membrane are captured with better resolution with TEM. This phenomenon can be understood by considering that electrons scattered by the samples on the top membrane experience further scattering and lose energy as they move through the liquid, which results in a loss of spatial and temporal coherence. This loss negatively impacts contrast mechanisms that rely on coherency of the electrons (e.g., phase or diffraction contrast) and lead to blurred images for objects on the top membrane. Similarly, it is also more difficult to obtain diffraction patterns from samples on the top membrane compared to samples at the bottom once the liquid layer is more than a few hundred nanometers thick because of plural scattering of the diffracted electrons and a significant background contributed by diffuse electron scattering in the liquid. Since EC–EM cells have fixed electrodes on one side of the cell, the sample location is fixed by the position of the working electrode, and so which optimal imaging mode to use depends critically on the design of the holder.

In the SEM, the presence of a top membrane with thickness in the tens of nanometers largely precludes the use of SEs as an imaging signal, and so, the primary imaging modes are BSE in setups with a single top window^140,141^ and SEM-STEM in setups with top and bottom windows.^142^ Here, the liquid layer thickness also plays a crucial role in determining image resolution. Like TEM, thicker liquid layers increase the noise reaching the detectors, making it more difficult to identify objects in the images. In addition, at the lower kV of SEM-STEM, the transmitted electron images undergo a contrast inversion in the range of liquid thicknesses commonly found in these electrochemical cells as the liquid layer thickness increases, which, in combination with Monte Carlo simulations, can be used to estimate the liquid thickness.^142^

An important point to note here is that while having a thin liquid layer is beneficial for obtaining images with good resolution and contrast we must be careful that this is not achieved at the expense of maintaining realistic reaction conditions. For electrochemical experiments, mass transport limitations and deviations from real-world behavior can arise from a liquid layer that is too thin. There has also been recent work proposing the use of electrochemically generated bubbles^195,233^ to deliberately reduce the thickness of the liquid layer in order to enable higher-resolution imaging and further analysis via spectroscopy or diffraction. While this approach does allow for more advanced EM techniques, there is a trade-off in our opinion because the catalysts are no longer experiencing realistic reaction conditions. The bubble generally dewets the entire working electrode and drastically alters the reaction environment. Furthermore, the thin liquid conditions that exist after bubble formation can result in a lower threshold for observing beam-induced effects due to the limited transport of radiolytic species away from the illuminated area^234^ or an increase in the local temperature during imaging.^235^ Another way to improve the spatial resolution is to make window materials as thin as possible, while maintaining the structural integrity of the membranes. In this instance, graphene is another common encapsulating membrane in liquid cell TEM studies^137^ but has not been used much for *operando* studies because incorporating external stimuli, such as heat or electrical pulses, into these so-called graphene liquid cells remains a significant technological challenge. In an alternative approach, Nagashima et al.^183^ showed that by overlaying the entire window of a MEMS chip with a Pt working electrode that has an array of holes it can provide needed rigidity and reduce bulging, thereby allowing them to obtain liquid layers that are ∼100 nm thick in the window corners. By combining this unique cell geometry with energy filtering, they were able to achieve lattice resolution during electrochemistry experiments at a relatively high dose of a few hundred electrons per Å^2^ as compared to the tens of electrons per Å^2^ or less, used typically in recent work, but this dose was sufficient to avoid Pt redeposition.

Resolving the behavior of a single atom or small cluster catalysts during reaction is most likely only possible for gas-phase catalysis studies, assuming that we can avoid beam-induced artifacts at those high magnifications. To improve the spatial resolution and contrast for imaging these nanoscale objects, the thickness of the membrane window needs to be drastically reduced while still being able to sustain atmospheric pressures within the cell without failure. Here, the most likely approaches will be smaller but thinner windows supported on thicker frames or hybrid membranes that incorporate 2D material membranes on holes in the silicon nitride or multimembrane stacks.^138^

### 4.2 Temporal Resolution

The temporal resolution of *operando* EM is largely determined by the technical specifications of the detectors, the minimal signal required to produce an interpretable image in the detector and the tolerance to the sample or its associated dynamics to beam-induced damage or artifacts. Current electron cameras can achieve image acquisition rates up to several thousand frames per second, but such high frame rates are rarely used in *in situ* experiments studying heterogeneous catalysts due to much higher electron flux required to generate images with reasonable signal-to-noise ratios at these rates.^236−238^ This high electron flux makes it difficult to avoid beam-induced artifacts under high-resolution imaging conditions, and typical image acquisition rates for TEM tend to be in the tens of frames/s. In STEM and SEM, the scanning mode of image formation largely limits image acquisitions to no more than 30 frames/s.^239^ Here, we anticipate general improvements in terms of temporal resolution in the future with the increasing adoption of high performance electron detection cameras that have better quantum efficiency^240^ for image acquisition.

As we mentioned early in Section 2, another key consideration related to the temporal resolution is the inherent dynamics of the catalytic processes that we are trying to observe. Figure 4 extracted from ref (46) summarizes the key dynamic processes that can occur during a catalytic reaction with their length and time scales, where the boxes denominate the length of time scales accessible by conventional TEM and conventional mass spectrometry, respectively. In general, most solid-state structural transformations found in catalysis are accessible with a modern EM, but capturing the dynamics of evolving objects comes with its own challenges especially for high-resolution TEM imaging, where the catalyst particles need to be optimally oriented and stable during the time frame of acquisition. This difficulty is also clearly illustrated in Figure 5 from work by Vincent and Crozier as we had discussed previously in Section 2.2. It should further be realized that such high spatial and temporal resolution imaging is often also associated with stronger beam-induced artifacts due to the higher dose requirement for good signal-to-noise ratios in the images.

Similarly, motion blurring can impact liquid-phase TEM studies especially if the catalyst particles are loosely bound to the membrane/electrode surface. It is largely accepted now that the particles captured by *in situ* TEM images are not free particles that are moving via Brownian motion but are particles that are either directly bound or adsorbed on the membrane surface,^237,238,241−243^ leading to additional metal–support interactions. These surface-bound NPs can still exhibit rotational motion,^237,238,243^ and the blurring induced by transient NP motion will impose a limit on the morphological information we can derive from studies where particles are mobile during the experiment. For example, images collected of a rotating particle at frame rates slower than its characteristic rotation rate will be a convolution of the particle’s orientation at different time points^238^ and may result in the loss of detailed shape information (i.e., a faceted particle can look round). The combined effects of motion blurring and the degraded resolution in a liquid generally make it harder to determine the orientation and facet exposure of small NPs from the *in situ* images.

### 4.3 Probing Chemical Changes Using Concurrent Spectroscopy

Another important capability of EM is the ability to provide local chemical information via spectroscopic techniques enabled by electron–sample interactions such as EDX or EELS. In combination with STEM imaging, these spectroscopic techniques can provide highly detailed information about the distribution of elements and the oxidation state of the constituents at atomic resolution. However, extending these techniques toward the *in situ* mapping of chemical changes in catalysts under reaction conditions is not straightforward. For one, the acquisition of a single elemental map with either technique still takes several minutes, which means that they cannot keep up with the changes in catalyst morphology that likely take place faster than the time needed to acquire a reasonable map. Nonetheless, recent improvements in spectrometer technology, such as the use of direct electron detection cameras for EELS,^244^ array detectors for EDX,^245,246^ and new mapping strategies such as multiframe acquisitions^247^ can pave the way forward toward chemical mapping at the higher refresh rates that will make spatially resolved spectroscopy relevant for catalytic studies.

Generally, EDX can allow us to track the chemical composition of catalysts during reaction, but the application of EDX in *operando* studies in liquids and gases is very limited due to the long acquisition time required with conventional spectrometers. This is, nonetheless, only a limitation imposed by the collection efficiency of current detectors. EELS, on the other hand, has seen wider application, particularly for its use in monitoring gas composition during thermal catalytic reactions through the identification of absorption edges of the gas species.^155,156,248,249^ In liquids, EELS is commonly used to estimate the liquid layer thickness using the log-normal method,^178,250^ but measuring absorption edges and performing core-loss EELS with a fully filled liquid cell is extremely difficult due to significant background in the spectra contributed by the liquid. As mentioned earlier, one way to improve the spectroscopic collection that is being increasingly applied is the formation of a bubble within the cell. Recently, there has also been increasing efforts based on advanced data analysis to improve the data we extract.^251−254^ The latter is especially valuable for the particularly noisy data from short integral acquisition-time EDS mapping^254^ or for low electron-dose sparsely sampled data sets.^255^

### 4.4 Electron Diffraction

Electron diffraction^256−259^ is an often underutilized technique for investigating catalysts inside the TEM under reaction conditions, even though it comes with several benefits. For one, the technique requires very low electron doses. Furthermore, acquisition times are fast because of the short wavelength of the electrons, which means that the Ewald sphere is almost flat and intersects with many reciprocal lattice points simultaneously. Due to the localized electron probe, the spatial resolution is higher compared to XRD, and we can obtain structural information from small crystals, which means that it has a higher sensitivity to impurity phases. The reflection intensity is also higher for high index reflections, giving rise to a higher structural sensitivity. The reflection intensity is, however, strongly dependent on the orientation of the NPs relative to the electron beam and can be affected by dynamic scattering processes. Note that the scattering power of an element also depends on the type of radiation that is used. For XRD, the atomic scattering factor (*f*^XRD^(*s*)) is proportional to the Fourier transform of the electron density (ρ(*r*)) as shown in eq 1:
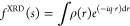
1while the atomic scattering factor for electrons (*f*^electron^(*s*)) is based on the atomic Coulomb potential Φ(*r*) as follows from eq 2:
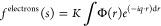
2with
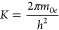
3
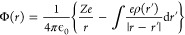
4where *m*_0_ and *e* are the mass and charge of the electron; *h* represents the Planck constant; *Z* is the atomic number; ε_0_ denotes the permittivity; *q* depicts the scattering vector (*q* = 4π*s*); *s* = sin(θ_2_)/λ; θ is the scattering angle; and λ denotes the corresponding wavelength.

**Figure 23:**
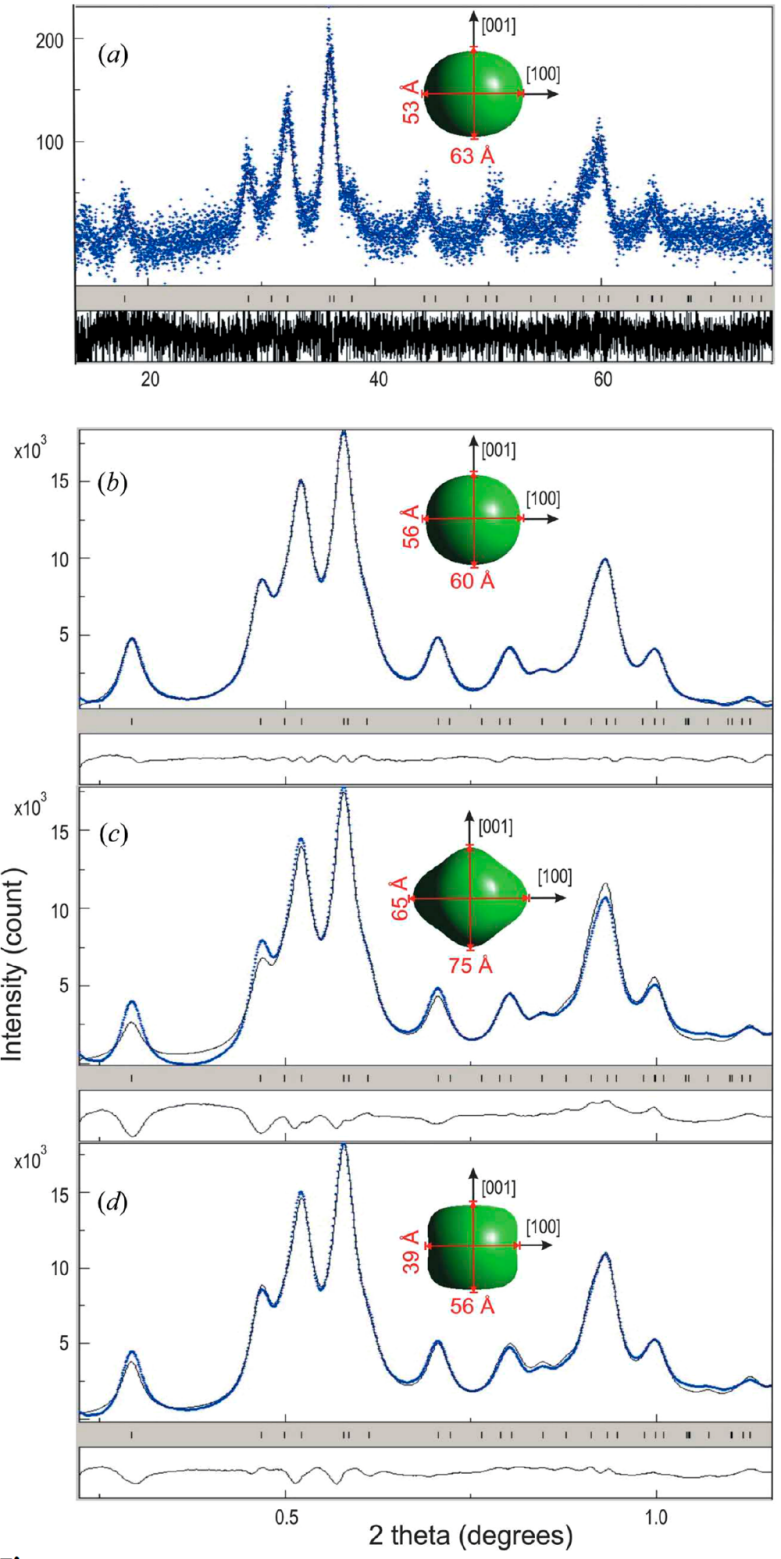
Rietveld refinement of ED data. Results of the combined analysis of Mn_3_O_4_ nanopowders for (a) XRD and ED patterns treated (b) using a pattern-matching mode (Le Bail), (c) using kinematical approximation, and (d) using a kinematical approximation with Blackman two-wave dynamic correction. The average size and shape estimated from the refinement of Popa coefficients (up to R3) are given. Reproduced from ref (268) with permission from the International Union of Crystallography.

This renders electron diffraction sensitive to light elements. Electron diffraction under reaction conditions can allow us to qualitatively conclude on the formation phases, peak asymmetries, or peak broadening. An example of how the technique can be beneficial in *operando* TEM research can be found in ref (53). A quantitative evaluation, however, remains challenging due to the presence of dynamic scattering processes. It has been shown that even for nanoscale particles with isotropic ring patterns dynamic electron scattering cannot be fully ruled out, and quantification using Rietveld refinement requires dynamical corrections such as the two-beam approximation (Figure 23).^260,261^ Analysis software, such as MAUD,^262^ is freely available for this. Conversely, precession electron diffraction, where the electron beam is tilted and rotated in the form of a hollow cone, is an empirical way to mitigate the effect of dynamical scattering.^263^ In this case, the diffraction is the average of the diffraction patterns taken at each incident beam direction in the precession cone. It means that the influence of any diffraction pattern exhibiting strong dynamical effects is reduced by averaging in the final pattern.

A prerequisite for harvesting quantitative information from electron diffraction, such as phase fraction, lattice parameter, etc., is the accurate measurement of diffraction data. This is however not always trivial due to the bulging of the chips and the related variation of the *z*-position of the sample. It requires precise alignment and a highly parallel electron beam.^264^ Furthermore, adequate compensation of elliptical distortions is essential, which can nowadays be achieved by effective machine learning algorithms.^265^ Those high-quality electron diffraction patterns can then also be used for pair distribution function (PDF) analysis.^260,263,266^ This total scattering approach allows us to conclude on the nearest neighbors (local structures), domain, and particle sizes. This technique has been powerful enough to conclude on local structures in amorphous IrO_*x*_ or AuAg NPs.^263,267^

Another technique associated with electron diffraction that is increasingly being used as an imaging method to provide structural and chemical information about a sample is 4D-STEM^240^ using pixelated detectors. For example, it has been shown that one can capture the orientation of individual NPs in a liquid cell with this method under conditions where the liquid layer was reduced due to H_2_ bubble formation,^194,195^ as highlighted in Figure 18e. While examples of 4D-STEM in *operando* EM experiments are still uncommon, we envision an increase in the adoption of this technique for studies in the near future.

### 4.5 Balancing Magnification, Frame Rate, and Signal-to-Noise Ratios

Based on the earlier discussions in this section, it should be clear that there is often a need to make certain compromises in terms of achievable spatial and temporal resolution if we are to avoid beam-induced alterations of the sample and record phenomena that are representative of their real-world counterparts. Here, we will outline a general framework for rationalizing this balance based on the safe assumption that we will, in most cases, work under a dose-limited imaging regime since the primary motivation of any *operando* EM experiment is to derive meaningful insight on a catalyst’s structure/composition during a catalytic reaction. Conversely, it means that an experiment is only feasible if we can obtain image sequences with images that have enough signal-to-noise ratios (SNRs) to be interpretable at the spatial and temporal resolution needed without introducing artifacts with the electron beam.

In EM imaging, the conventional standard for a reliable image quality (also known as the Rose criterion) is that the corresponding pixels of an object need to have a SNR of at least 3 over its background.^173,269^ In low-dose routine imaging and gas cell TEM, this background is usually the camera shot noise due to the absence of electron scattering in vacuum or the limited scattering of electrons by the gas molecules. In liquid cell TEM, the background is typically the diffuse signal contributed by electrons scattered by the liquid. This idea of a minimum SNR needed for interpretation combined with the maximum dose that a sample or experiment will tolerate can then be summarized into a dose-limited resolution^173,269^ for a specific material, a specific contrast mechanism, and a given camera performance to inform how we set up the imaging conditions in an *in situ* EM experiment. The optimal imaging condition is one where we do not over- or undersample the spatiotemporal resolution. For example, the pixel resolution should not be more than half the feature size that needs to be resolved (Nyquist limit) but not much less than dose-limited resolution; otherwise, the electron dose required to generate images with good signal-to-noise may exceed the threshold for significant beam damage. For liquid cell EM, where the dose limits are much lower, it usually means that we will be working at a magnification that is significantly lower than what a modern TEM is capable of. Note that these conditions become more stringent if we include chemical/compositional mapping or analysis.

## 5 Perspectives on the Further Directions of the Field

Despite the critical tone of some of the aspects described in this review, we firmly believe that *operando* EM will play a decisive role in the elucidation of the principles that govern heterogeneous catalysis, but not by imaging the active site per se. In our opinion, the true strengths of *operando* EM experiments lie in the detection of frustrated phase transitions, metastable states, and changes in the bulk structure or morphology of particle assemblies. It is also ideally suited for long-term or deactivation experiments if there are no logistical limitations (note that the time scales that are considered long-term can be different for thermal catalysis and electrocatalysis).

As we have described so far, the field of *operando* EM is still immature with plenty of room for further improvements in instrumentation, especially in terms of time resolution for both imaging and function determination and more realistic reactor designs to minimize undesired confinement effects. Further efforts to exploit the low dose imaging methods and imaging strategies that have been developed for the cryo-EM of biological specimens and in-depth electron diffraction data analysis can help with minimizing and mitigating beam-induced artifacts in these studies and increase knowledge about the catalysts.

### 5.1 Instrumentation Improvements and Method Development

We expect future developments in terms of instrumentations and methods to be focused in two areas: (1) holder and reaction cell designs that attain more realistic reaction conditions or are equipped with more analytical capabilities to provide information about the reaction process, and (2) computational efforts into automated data acquisition and data-science/artificial intelligence-assisted data analysis of images and spectra and also into the theory of such diverse operating catalyst particles using insights from the *operando* studies.

In terms of reactor design for thermal catalysis, a way to read the pressure and temperature directly at the catalyst particles is crucial, but such measurements are not trivial to perform. A solution could come from a piezoelectric sensor on the MEMS chip. Furthermore, in order to improve the flow conditions inside the nanoreactor, a movable hybrid between the nonwoven silica approach of Crozier and co-workers combined with the closed-cell design seems promising and may enhance the interpretative depth of *operando* TEM investigations. Similar development of integrated sensors is also expected for electrocatalysis, where in this case the local pH is the key read out. In addition, reaction designs can also be improved to replicate more accurately real reaction conditions (such as high-pressure cells for thermal catalysis) or have the flow channels optimized for faster online product detection at higher sensitivity.

We also expect that the research activity studying the behavior of electrocatalysts under reaction conditions will increase as EC-TEM gets more established. Although the imaging resolution in liquid is somewhat limited, EC-TEM does provide unique structural information that cannot be attained easily using other means. In particular, we anticipate that future work will go beyond imaging structural changes and head toward tracking the catalyst chemical composition or oxidation state to extract complementary data about the processes that control the structural transformations. Thus, the behavior of electrocatalyst ensembles will be followed via wide field-of-view observations and using *in situ* acquired electrochemical data for further quantification of the catalyst properties.

The next aspect pertains to how to keep track of the various key experimental parameters during *operando* EM experiments and how to handle the wealth of data we obtain from these studies. Simply put, neither is amendable to manual handling, and so extensive development of automated and analytical approaches to both survey and characterize in-depth the ensemble of samples during *operando* experiments is required. In this aspect, we expect that the following data acquisition and analysis approaches will become increasingly integrated into the workflow of future *operando* experiments: (i) automated sample acquisition routines for large area imaging,^270^ (ii) on-the-fly image analysis enabled by machine learning enhanced image segmentation,^271,272^ and (iii) special software that enables better tracking of metadata such as the imaging conditions, electron dose,^273^ and catalysis parameters. More importantly, as we mentioned earlier, it is very difficult to determine the catalytic impact of the structural changes we observe simply from the image, and we expect a significant role of theory to rationalize the underlying processes and the complicated convolution of the different parameters.^57,274^ Image-free representations of EM data are thus required to feed computers and to allow for improved causal correlations. The conversion of 2D TEM image stacks into meaningful observables and their representation as multidimensional plots also offer opportunities for novel denoising and reconstruction algorithms,^275^ data management, and visualization strategies. In particular, it will not be practical in the long-term to store tera-bytes of raw EM data due to storage costs without some form of data reduction or compression.^276^ This will be particularly important when *operando* EM are likely to generate sparse data sets with a large percentage of background and noise information in the images, due to the low electron dose imaging protocols.

### 5.2 Impact of Machine Learning and Artificial Intelligence on the Conduct of *Operando* EM Experiments

Beyond the benefits of machine learning for data processing and analysis, we also expect that artificial intelligence methods will change how we work and the types of samples/reactions we can investigate in experiments in the future. Our interpretations are often limited by what we can easily visualize, which in EM means objects showing strong mass or crystalline contrast. However, one should not simply associate the dominance of these features with their relevance in the catalytic process, and so having sufficient statistical support is essential. Nonetheless, the limitations of current segmentation methods mean that we still cannot decipher more subtle features in the samples. The continued development of better computer vision routines will eventually allow us to start interrogating more complex samples such as industrial catalysts, where the more complicated interplay between the various sample and experimental parameters is not easy for a human scientist to rationalize directly. Looking further ahead, the next step of this evolution will be in autonomous *operando* EM experiments where the routines for microscope control, data acquisition, on-the-fly image processing and analysis, cross-correlation with electrochemical data or product analysis, and physical model comparisons are connected in a continuous workflow.^277^ These approaches can help us to arrive faster at meaningful conclusions by moving away from the current linear experimental designs (e.g., temperature ramps, partial pressure variation, or time dependence) and toward partially or fully self-driven studies. Here, the human scientist or an artificial intelligence algorithm can actively intervene in the ongoing experiment to avoid unnecessary sampling as hypotheses are eliminated by results from the emerging data sets. Finally, we expect the cumulation of these efforts to both make these currently challenging *operando* EM experiments easier to perform and at the same time allow us to tackle complex research questions that are closer to real catalysts and real reactor conditions and hopefully allow us to finally unravel the mystery that is catalysis.

Here, we also envision the role of the electron microscopist in these experiments changing, as automated and autonomous microscopy becomes closer to reality.^277^ Automated sampling and object classification routines^278,279^ will free the microscopist from the more mundane jobs of image acquisition and analysis, thus opening the door to rapid and effective acquisition of large data sets. The statistics that can be extracted from such data sets will in turn enable a robust differentiation of relevant catalytic processes and irrelevant solid-state transformations and thus provide deeper understanding into the actual nanoscale structures that are responsible for catalyst activity and selectivity. Analysis using computer vision may also reveal the importance of minority species and subtle structural features. It means that the microscopist will become more connected to the research and the design of experiments.

### 5.3 Potential of Complementary Integral *Operando* Techniques

Another aspect that we expect will gain significant traction is the use of several techniques in combination with *operando* EM to investigate a catalytic process. As the relevant length and time scales of catalytic processes can span several orders of magnitude, no single instrument or experimental method is capable of providing complete information about a catalyst. Thus, a multimethod approach will have to be implemented, and such an approach might involve the complementary use of X-ray beams using the same *in situ* reactor holders.

While *operando* EM can contribute to a better understanding of the catalytic processes by providing insight into the local structural motifs that exist under reaction conditions, we must always bear in mind that EM, especially TEM with its high spatial resolution, can be a very self-selecting method, since the amount of sample that we can realistically probe is very small and makes up only a small section of the sample. Moreover, we are only looking at the sample surface with SEM or samples that are thin enough to be electron transparent with TEM, while larger agglomerates or bulkier structures cannot be investigated with either method and are often ignored in the sample surveys. This concern with statistical sampling is similarly applicable to *operando* EM studies. Here, the inherent conflict lies in the desire to work at high enough magnification such that high-resolution dynamics can be revealed versus working at a broader field of view to ensure that the observed dynamics are consistent across all the particles or that the behavioral variations across different particles are captured. The latter is extremely important for establishing catalyst function analysis since the catalytic properties we measure using different techniques are averaged over the entire ensemble.

However, unlike conventional TEM work where it is possible to measure a few hundred particles from one sample in a single sitting to build up important particle statistics, such as particle size distribution, the dynamic nature of *operando* studies limits how many catalyst particles or how many sets of catalyst particles can be imaged in an individual experiment. Another related concern is the relevance of the observed dynamics or transformations as we had discussed earlier. While one can reasonably argue that a transformation that occurs together with a change in catalytic behavior is correlated with the change in catalytic function, assigning the role of the structure is less straightforward. We need to be particularly careful when we interpret our observations, especially in terms of claiming how the observed stability of a certain crystallographic facet can be used to infer its dominance in the catalytic reaction because the stable imaged structure may in fact be the inactive state, and the real active structure is found in the subtle or transient fluctuations that are beyond our ability to capture. Moreover, investigations from one local perspective are usually by far insufficient to fully describe any catalytic system.

Due to these considerations, complementary investigations must be made to unravel the importance of the observed structures for the catalytic reaction. The combined assembly of data obtained from different complementary techniques has been gaining in importance and has been used to extend our view on the working principles of heterogeneous catalysts.^15,38,96,280−282^ The results have been brought together and have been jointly interpreted to obtain a solid level of our current understanding. However, the concept of complementarity should be restricted not only to analysis and characterization of such functional materials but also to synthesis and testing. In terms of synthesis, complementarity refers to the preparation of sample families in sufficient amounts for which one parameter is systematically varied, and all tests and analysis can be conducted from the same batch. Ideally, this parameter variation would proceed fully automated, reducing the sources of errors. Complementarity in terms of catalytic testing utilizes different setups, reactions, and conditions. The obtained results can be subsequently correlated to spot the differences. An illustrative example could be the hydrogenation of CO_2_ where different CO_2_ to H_2_ ratios can tune the reaction mechanism ranging from reverse water gas shift (RWGS), over methanol synthesis, to methanation. The reaction would be, thus, an ideal candidate to study the influence of the reaction mechanism and to probe the product selectivity of one specific catalyst.

In general, complementary investigations can be grouped into local–local or local–integral techniques and involve comparative studies judging the relevance of the local study for the entire system. Local–local techniques combine, for instance, *operando* (S)TEM with *quasi in situ* (S)TEM, which allow insights into the presence of beam damage or correlative microscopy techniques of identical samples in the same environment. Common groupings of correlative techniques include SEM and 2D Raman spectroscopy or TEM and XAS.^283^

Local–integral complementary techniques combine spatial resolution with structural averaging of different parts of the functional material. Examples of integral techniques that are complementary to the different local information that can be obtained from modern TEMs are listed in Table 3.

**Table 3:** Examples of Complementary Local-Integral Techniques to the Most Commonly Applied TEM Investigations

Local	Integral	Information
High-resolution (S)TEM	XRD; Rietveld analysis, XRR	Crystalline, amorphous bulk phase
High-resolution (S)TEM	XRD; whole powder pattern modulation	Defects, strain
High-resolution (S)TEM	XPS; LEIS; FTIR, XRR, XRDS	Surface structure
TEM	XRD; Rietveld analysis; Raman spectroscopy	Particle size distribution/coherent scattering domain (number weighted)
TEM	—	Particle distribution
TEM	SEM	Geometric information
TEM	Physisorption	Porosity, specific surface area
EDX, EELS	X-ray fluorescence, ICP-OES/XPS	(Bulk) composition/surface composition
EELS	NEXAFS, UV/vis spectroscopy	Electronic structure
Electron diffraction/CBED	XRD	Phase
Pair distribution derived from electron diffraction	PDF, EXAFS	Next neighbors

All complementary techniques can also be applied as *operando* or *in situ* techniques. Most of the integral *in situ* techniques were developed for gas-phase reactions. To account for liquid-phase reactions, several dedicated cells have been developed including TEM, XPS,^141,284,285^ XRD,^286^ XAS,^286,287^ and Raman spectroscopy.^288,289^ Recently, a grazing incidence (GI)XRD cell has been presented that allows for the simultaneous acquisition of surface-sensitive XRD and electrochemistry data.^290^ Surface oxidation and passivation of metallic Cu was studied to prove the concept. The setup allows us to obtain realistic cyclovoltammetry (CV) and chronoamperometry (CA) curves. Simultaneously, GIXRD measurements showed that cuprite is formed prior to the formation of the superficial tenorite phase that passivates the sample. The quality of the GIXRD data further allows for Rietveld refinement to assess the phases present. The combination of subsecond time solution XAS and XRD was also demonstrated in another study,^286^ which enabled the tracking of the Cu phase formed from Cu_2_O during pulsed electrochemistry. By mapping the phases present against the reaction products generated, it was shown that an optimized dynamic balance between oxidized and reduced copper surface species created within a narrow range of cathodic and anodic pulse durations led to a 2-fold increase in ethanol production when compared against static CO_2_RR conditions. Transmission X-ray microscopy (TXM) studies using electrochemical cells like those used for TEM are also attracting attention. Recently, it was demonstrated that scanning TXM enables the spatially resolved tracking of a β-Co(OH)_2_ catalyst’s oxidation state at different potentials during OER.^291^ Here, because of the weaker attenuation of X-rays, spectro-microscopy measurements of the absorption edges within a liquid environment are easier than in TEM. Combining TXM with TEM will also be particularly interesting for *operando* work, where the spatially resolved electronic information can be complemented with geometric information on the identical sample.

A reliable comparison of catalytic results and mechanisms requires not only the investigation of structurally and compositionally identical samples but also the use of identical operation conditions in gas and liquid environments (composition and quality), temperature, and pressure or applied potential as well as reactor design. Here, a significant burden of proof also lies in establishing that the results from different instruments are comparable and that the different techniques are probing the identical reaction environments. This challenge can be addressed in two ways: (i) a portable microreactor that can be moved from one system to another^292^ (i.e., consistent reactor design) or (ii) having a reactor chamber that integrates multiple detectors, which is more likely to be possible on an ESEM. These experiments are starting to produce very valuable information on working catalysts and will provide a more complete picture of the catalytic processes by providing concurrently acquired complementary data, albeit still in a subset of the full complexity of a real-world system.

## 6 Concluding Remarks

The recent advances in *operando* EM have dovetailed fortuitously with the pressing urgency to develop novel functional heterogeneous catalysts for energy applications and has greatly accelerated interest in the technique. Being able to exploit and augment the potential of *operando* EM studies for heterogeneous catalysis research, despite their current limitations, is a critical element in our endeavor to arrive at a causal functional description of catalysis. To this end, the kinetics and dynamics of catalyst materials need analytical descriptions on several scales of time and space, ranging from molecular to reactor dimensions and from charge carrier dynamics to transport characteristics of mass and energy. Having access to information on the real structure and morphology of a material is key to describing the kinetics and dynamics of catalytic interfaces responding to the local chemical potential and its variation.

A combination of microscopic techniques ranging from TEM to SEM, then on to optical, together with scanning probe microscopy and observations of elastic to inelastic interactions of electrons (and photons) with the working catalyst in a correlative manner is required. Such studies are still ahead of us. Any of these methods are of equal relevance as they probe a different section of the space–time diagram presented here. A debate about which microscopy method is best suited is thus inadequate. It would be useful to focus each of the observation techniques on those aspects for which they are best suited from their physical characteristics and not try to answer all aspects with a single approach. This is our request to those developing *operando* microscopy centers to have and plan for multi-instrument facilities if the main purpose of the work is to understand materials during chemical reactions.

At present, we barely master the challenges involved in using any one of the methods to study catalysts during short episodes of operation. Representative data collection in space and time and systematic variation of reaction conditions are still the exception in present studies. What we rarely see is the direct correlation of microscopic with kinetic observations requiring the transformation of image information in numerical descriptors. Even more rarely do we find correlations of *operando* kinetic data with data obtained in a dedicated kinetic experiment. Such correlative observations would serve to verify the quantitative connection between structure and function across the dimensional gap between catalytic reactors and *operando* reactors.

*Operando* microscopy carries further the barely exploited potential to study the initiation and growth of deactivation phenomena. As shown in numerous examples in this work, one can understand them as the completion of phase transitions in the catalyst induced by the reaction conditions and started by the nucleation of active sites. If we are able to shed quantitative light on these transition processes, we may make catalysts more sustainable (longer life and stable selectivity). Present high-performance catalysts with their complex chemistry are to a substantial extent the result of empirical optimization of their sustainability that occurred in the absence of knowledge of kinetic and dynamics of the chemical-phase inventory of the material. By reducing their chemical complexity, we may contribute to a clearer understanding of the chemical role of each component relevant for a mechanistic description and likewise to a better recyclability.

Rigorous digitalization of microscope hardware and data analysis and the simultaneous use of several observation techniques with the same specimen will be future requirements to approximate the scientific target of *operando* microscopy. Cooperation with spectroscopy in various energy ranges allows coupling between *operando* microscopy and *operando* spectroscopy into a comprehensive representative description of a working catalyst in its target reaction. To this end, the infrastructure of a “chemical observatory” will be needed that gives the infrastructural frame of correlative analysis. *Operando* microscopy with its wide range of techniques is likely at the core of such an observation. In this way, we answer the title question by stating that *operando* microscopy is in fact a cornerstone of catalysis science.

Nonetheless, the path forward requires a collective awareness by both the catalysis and the electron microscopy communities that we need to standardize the reporting protocols and the workflows for the conduct/documentation of the actual and control experiments for the acquired data to be useful for inspiring future catalyst discovery. These technical issues can be and should be addressed by mindful action on the part of researchers working in the field. Further growth of the field also requires the transition from a phenomenological description of structural changes in a catalyst to the more fundamental science of catalyst structure–property correlation. It is important that we do not only reproduce what has already been discovered by other techniques but move toward providing unique insight into the complexity and diversity of morphologies that can exist under reaction conditions. This means a systematic and massive use of our existing methodologies as described in the text. Their data-centric and broad-based use is more important than searching for additional techniques. We will be missing out on significant discoveries if we reduce everything into a naïve picture of time-independent uniform working catalyst particles. To quote Albert Einstein, “Everything should be made as simple as possible, but not simpler”.
